# Auxin Influx Carriers Control Vascular Patterning and Xylem Differentiation in *Arabidopsis thaliana*

**DOI:** 10.1371/journal.pgen.1005183

**Published:** 2015-04-29

**Authors:** Norma Fàbregas, Pau Formosa-Jordan, Ana Confraria, Riccardo Siligato, Jose M. Alonso, Ranjan Swarup, Malcolm J. Bennett, Ari Pekka Mähönen, Ana I. Caño-Delgado, Marta Ibañes

**Affiliations:** 1 Department of Molecular Genetics, Centre for Research in Agricultural Genomics (CRAG) CSIC-IRTA-UAB-UB, Barcelona, Spain; 2 Department of Structure and Constituents of Matter, Faculty of Physics, University of Barcelona, Barcelona, Spain; 3 Institute of Biotechnology, University of Helsinki, Helsinki, Finland; 4 Department of Biosciences, University of Helsinki, Helsinki, Finland; 5 Department of Plant and Microbial Biology, North Carolina State University, Raleigh, North Carolina, United States of America; 6 School of Biosciences and Centre for Plant Integrative Biology, University of Nottingham, Nottingham, United Kingdom; National University of Singapore and Temasek Life Sciences Laboratory, SINGAPORE

## Abstract

Auxin is an essential hormone for plant growth and development. Auxin influx carriers AUX1/LAX transport auxin into the cell, while auxin efflux carriers PIN pump it out of the cell. It is well established that efflux carriers play an important role in the shoot vascular patterning, yet the contribution of influx carriers to the shoot vasculature remains unknown. Here, we combined theoretical and experimental approaches to decipher the role of auxin influx carriers in the patterning and differentiation of vascular tissues in the Arabidopsis inflorescence stem. Our theoretical analysis predicts that influx carriers facilitate periodic patterning and modulate the periodicity of auxin maxima. In agreement, we observed fewer and more spaced vascular bundles in quadruple mutants plants of the auxin influx carriers *aux1lax1lax2lax3*. Furthermore, we show AUX1/LAX carriers promote xylem differentiation in both the shoot and the root tissues. Influx carriers increase cytoplasmic auxin signaling, and thereby differentiation. In addition to this cytoplasmic role of auxin, our computational simulations propose a role for extracellular auxin as an inhibitor of xylem differentiation. Altogether, our study shows that auxin influx carriers AUX1/LAX regulate vascular patterning and differentiation in plants.

## Author Summary

The vascular tissues in the shoot of *Arabidopsis thaliana* (Arabidopsis) plants are organized in vascular bundles, disposed in a conserved periodic radial pattern. It is known that this pattern emerges due to the accumulation of the phytohormone auxin, which is actively transported by the so-called efflux and the influx carriers. Efflux carriers facilitate polar transport of auxin from inside the cell to the extracellular space, while influx carriers pump auxin from outside the cell to its interior in a non-polar manner. Although a role for auxin efflux carriers in the emergence of this pattern has been recognized, the role of auxin influx carriers has remained hitherto neglected. In this study, we combine theoretical and experimental approaches to unravel the role of the auxin influx carriers in the formation of plant vasculature. Our analysis uncovers primary roles for the auxin influx carriers in vascular patterning, revealing that auxin influx carriers modulate both patterning and the differentiation of the water transporting vascular cells, known as xylem cells.

## Introduction

Auxin is an essential phytohormone for the control of plant growth and development. Its transport and distribution throughout the plant create numerous organized patterns in plant tissues, such as leaf venation [1], the wide variety of phyllotactic patterns [2–5], and the periodic shoot vascular patterning [6,7]. Auxin is also involved in the emergence of new organ primordia [4,8], root apical meristem maintenance [9,10], root gravitropism [11–13], lateral root development [8,14], and xylem differentiation [15,16] amongst other developmental processes.

A proportion of auxin is synthesized in the shoot apex and polarly transported in a cell-to-cell manner to the root and to other plant tissues [17]. The chemiosmotic model explains how auxin is polarly transported throughout the plant [18,19]. According to this model, once auxin enters the cell where the pH is less acidic (cytosol pH≈7) than in the apoplast (pH≈5.5), it becomes deprotonated; this hydrophilic form remains then trapped inside the cell. In order to exit the cell, auxin needs active protein transporters that can pump it out. The asymmetric localization within the cell membrane of a subset of these transporters or auxin efflux carriers named PIN (PIN-FORMED) [6] results into one of the main characteristics of auxin transport: its polarity. Depending on the positioning of the PINs, directional fluxes and auxin gradients are created, driving the accumulation of auxin maxima in specific groups of cells [3–5]. Disruption of auxin polar transport significantly alters auxin maxima distribution, resulting in aberrant development [2,6–8,12]. In addition to the PIN efflux carriers, the PGP (P-glycoprotein) ABC-like transporters also export auxin from the cytoplasm to the apoplast [20]. PGP transporters do not localize asymmetrically, but they have been proposed to interact with PINs, subsequently affecting auxin polar transport [21–23].

Unlike auxin efflux from cells, auxin enters the cells either by passive diffusion or by the action of auxin influx carriers. These comprise a multi-gene family in Arabidopsis containing four highly conserved genes: *AUX1* and the *AUX1*-like genes *LAX1*, *LAX2*, and *LAX3* [24–26]. In contrast with efflux carriers, influx carriers do not show a polar distribution within most cells with exception of the root protophloem cells [27,28]. The absence of strong phenotypes in influx mutants, especially under long day conditions, as well as their non-polar distribution has prevented extended studies on the role of influx carrier mutants on patterning in the past. So far, all the AUX1/LAX family members have been associated with changes in vascular transport [29], leaf positioning [30,31] and root stem cell patterning [32]. Despite the reported redundancy, *AUX1/LAX* family members not only have distinct tissue-specific expression patterns, but also exert different functions, suggesting that these genes underwent sub-functionalization and likely provided additional mechanisms of regulation to the plant [25,31]. For instance, within Arabidopsis primary root, AUX1 has been reported to localize in the columella, the lateral root cap, the epidermis and the stele [25,33,34], LAX1 is localized within the mature vascular tissue, with weak expression in the root tip immature vasculature [25], LAX2 is localized within the root stem cell niche, the provascular cells and the stele [25] and LAX3 is localized in the columella and the stele cells [25,35]. In addition, AUX1, LAX1 and LAX2 are differently localized at multiple developmental stages in the lateral root primordia whereas LAX3 is localized in the outer tissues in front of the primordia [25,35,36]. AUX1, the most studied influx carrier, has been attributed roles in root gravitropism, petal initiation and lateral root development [36–38]. Furthermore, LAX2 influx carrier has been recently reported to confer continuity to the vascular strands in cotyledons [25]. In addition, LAX3 has been shown to promote lateral root emergence and apical hook development [35,39]. Theoretical studies have proposed a stabilizing role for influx carriers on periodic patterning rather than major roles in pattern emergence [40–42]. Experimentally, the analysis of *aux1lax* mutants confirmed the stabilizing role in shoot phyllotactic patterning [31]. Phenotypes were visible under short day conditions, suggesting that AUX1/LAX transporters may be particularly relevant under certain environmental conditions. Nevertheless, the functional relevance of AUX1/LAX proteins in the periodic vascular patterning of the shoot remains unknown.

Here we provide theoretical and experimental evidence for a yet uncharacterized role of auxin influx carriers controlling periodic vascular patterning and the differentiation of xylem cells in plants. The vascular tissues in the shoot of Arabidopsis plants are organized in vascular bundles (VB), disposed in periodic repetitions along a circular vascular ring. Each VB is composed of meristematic procambial cells and of differentiated vascular cells termed xylem and phloem (S1 Fig, shown as grey, blue and green cells, respectively), which arise each from centripetal and centrifugal divisions of the procambial cells [43,44]. In between the VBs, interfascicular fibers (IF) differentiate, supporting the inflorescence stem [43,45] (S1 Fig, light blue cells). The analysis of quadruple knockout mutants of AUX1/LAX transporters disclosed fewer and more spaced VBs in the shoot of Arabidopsis. This phenotype is in agreement with our mathematical and computational modeling predictions, which show that auxin influx carriers facilitate periodic patterning and increase its periodicity. Furthermore, a reduced differentiation of the vascular cells is observed in both shoot and root tissues. Our data support that influx carriers promote cytoplasmic auxin signaling, which has been previously shown to drive xylem differentiation [15,16,46,47]. In addition, our modeling analysis predicts a novel role for apoplastic auxin as inhibitor of xylem differentiation.

## Results

### Auxin influx carriers increase the number of auxin maxima and facilitate periodic auxin patterning

We have previously shown that periodic auxin distribution is relevant for VB pattern formation [7]. In order to investigate the role of influx carriers on periodic distributions of auxin, we first performed a theoretical and computational analysis (Materials and Methods). A minimal modeling approach was selected by assuming that auxin polar transport sets auxin maxima along a ring of provascular cells, which ultimately drive VB emergence [7]. Albeit this approach with its simplified geometry cannot drive quantitative predictions, it is expected to provide key features underlying the role of influx carriers for periodic auxin patterns (S2 Fig). A previous model on auxin polar transport known to drive periodic auxin maxima [3,41] was considered and further elaborated by including auxin apoplastic diffusion [7] (Materials and Methods, S2 Fig). In the model, auxin uptake into the cells occurs actively through influx carriers as well as passively, while auxin exits the cells through polarly localized efflux carriers as described in [3,41]. The model also takes into account that the synthesis of both types of carriers, as well as the polar localization of efflux carriers, depends on auxin concentration [3,14,35,41,48,49]. Parameter values for auxin polar transport were chosen according to the literature (S1 Table) [34,50–54]. Both theoretical and computational analyses were performed through linear stability analysis of the homogeneous state and numerical integration of the dynamics, respectively (Material and Methods and S1 Text). The model drives periodic maxima of auxin concentration as expected [3,4,55] with influx and efflux carriers being more abundant in those cells harboring auxin maxima (S3 Fig). This localization of carriers arises from the auxin-induced synthesis of influx and efflux carriers, which was set in the model to embrace experimental evidences on the auxin-induced expression of carriers [14,35,48,49]. Yet, according to the modeling of auxin dynamics, the auxin-induced synthesis of carriers is not essential for pattern formation [3,4,55] (S4 Fig). We defined *I* as intensity of the active influx transport (S1 Text and S1 Table), which is proportional to the maximal amount of influx carriers a cell in the ring array can have. For simplicity, hereafter we use the term 'amount of influx carriers' for *I*.

**Fig 1:**
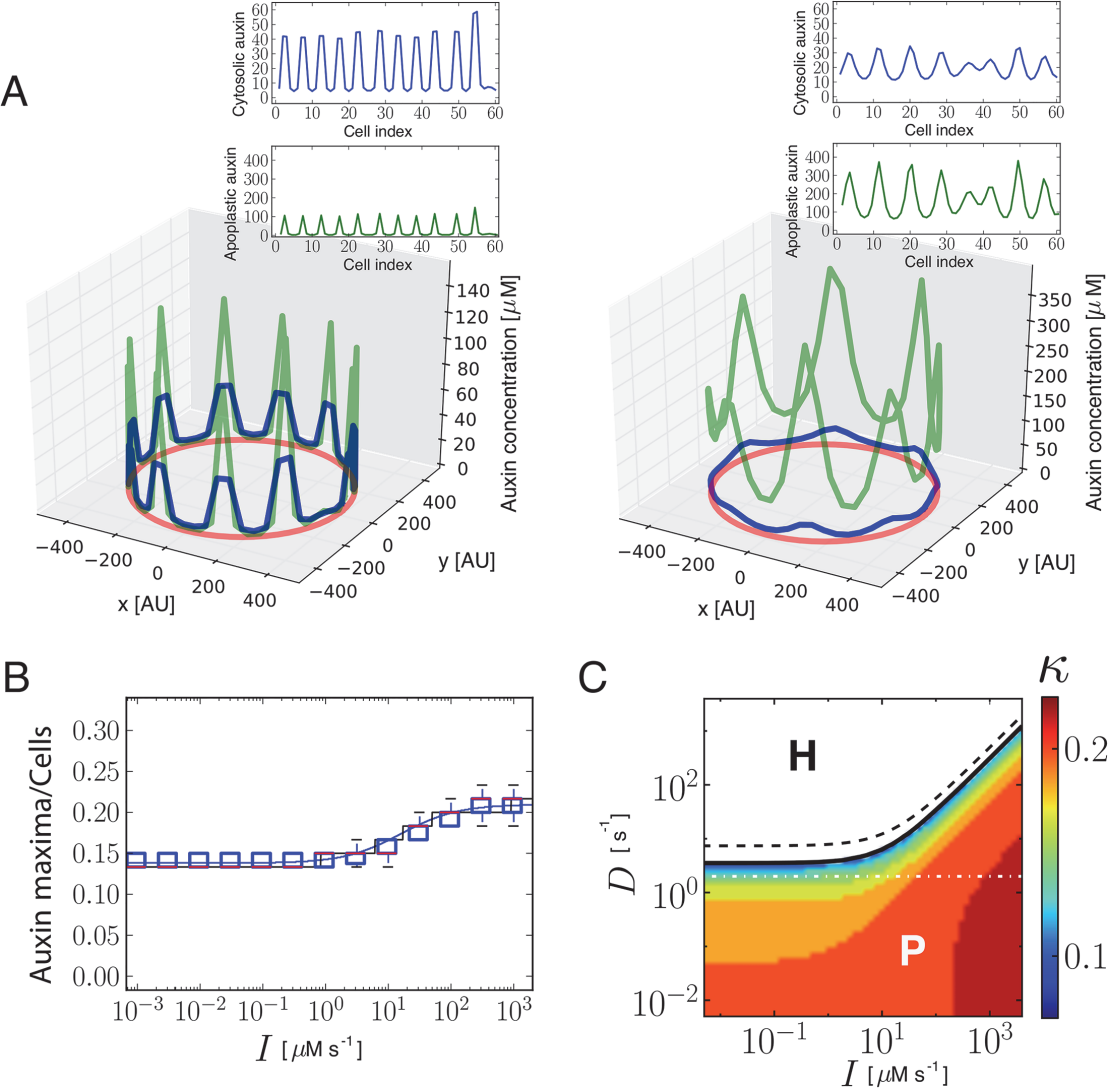
Theoretical and simulation results predict that auxin influx carriers facilitate periodic patterning and promote auxin maxima. (A) Snapshots of simulation results showing periodic distribution of auxin inside and outside cells for higher (left, *I* = 100 μM s^-1^) and lower (right, *I* = 0.001 μM s^-1^) influx carriers levels along a ring of vascular tissue composed of 60 cells surrounded by the apoplast. Cytosolic (blue) and apoplastic (green) auxin concentrations at time t = 17.5 are shown. The red circular line represents the ring of cells in the tissue. Insets depict the same results projected into a 2D plane. Space is represented in arbitrary units [AU]. Influx and efflux carriers distributions are described in S1 Text. (B) Simulation (boxplot) and theoretical estimation (*κ*, depicted by solid lines; see S1 Text) results of the inverse value of the number of cells between cytosolic auxin maxima at different influx carriers levels (*I*). Each boxplot depicts the results for 30 simulations with different initial auxin distributions (Materials and Methods). Simulations were done for rings of 60 cells. The bottom and the top of the boxes represent the first and third quartile, enclosing the 25%-75% data range, the red line within the box stands for the median, and the low and high whiskers enclose the 1.5×(25%-75%) data range. The theoretical estimation is performed through linear stability analysis for a ring of 60 and 1200 cells (black and blue lines, respectively). Theory and simulations show that influx impairment enlarges the periodicity of the pattern, increasing the distance between consecutive auxin maxima. (C) Phase diagram obtained from theoretical linear stability analysis on a ring of 60 cells on the parameters space of amount of influx carriers (*I*) and apoplastic diffusion coefficient (*D*). The solid line divides the space in two regions (Methods): in the H region (white, above the solid line) the homogeneous state is linearly stable and no periodic pattern can be formed from small perturbations of it. In the P region (colored, below the solid line) the homogeneous state is linearly unstable and a periodic pattern can arise. The dashed black line corresponds to an analytical approximation to the solid black line (S1 Text, Eq S34). The color scale shows the theoretical estimation of the inverse value of the average number of cells between cytosolic auxin maxima (*κ*). The horizontal dashed-dotted white line depicts the line along which simulations are presented in panels A and B. For low apoplastic diffusion *D*, periodic patterning can still occur at low influx parameter values *I*, while high influx values are necessary for patterning at higher apoplastic diffusion coefficients. Main parameter values: *E* = 105 μM s^-1^, *D* = 2 s^-1^, *D*_*ca*_ = 15 s^-1^ and auxin threshold for transporters activation *θ*_*I*_ = *θ*_*P*_ = 10 μM. Other parameter values can be found in S1 Text.

Our analysis predicts that the amount of influx carriers can control the periodicity of the pattern, driving changes in the number of auxin maxima (Fig 1A and S1 Video). When the amount of influx carriers is decreased, less auxin maxima arise in a ring with a fixed number of cells (Fig 1A right panel). Hence, influx carriers promote auxin maxima to be closer together in terms of number of cells, up to a limit (Fig 1B and 1C). While pattern periodicity modulation was previously associated only to efflux carriers [3,4], our modeling results unveil a novel role for influx carriers in this process. Auxin entrance into the cells is essential for periodic pattern formation, by enabling the polar transport of auxin to take place. We confirmed that passive entrance into the cells, independently from influx carriers, can be enough to sustain periodic patterning, as expected (Fig 1C). Yet, we found that influx carriers become essential for patterning in high apoplastic diffusion conditions, in which passive entrance of auxin into the cell is not enough to enable the periodic patterning (Fig 1C and S1 Text). Therefore, our results show that influx carriers promote pattern formation as well.

### Auxin influx carrier mutants have fewer vascular bundles in the shoot

To study the role of auxin influx carriers in the shoot vascular patterning, we first evaluated the expression pattern of the influx carriers’ proteins in the shoot. Radial sections at the basal region of the shoot inflorescence stem revealed that AUX1, LAX1, LAX2 and LAX3 fluorescent protein fusions show expression in the shoot vascular tissues (S5 Fig). This expression is localized at the VB (S5 Fig), where both auxin response and efflux carriers expression are known to occur [6,7,56] in agreement with the distribution predicted by modeling (S3 Fig).

**Fig 2:**
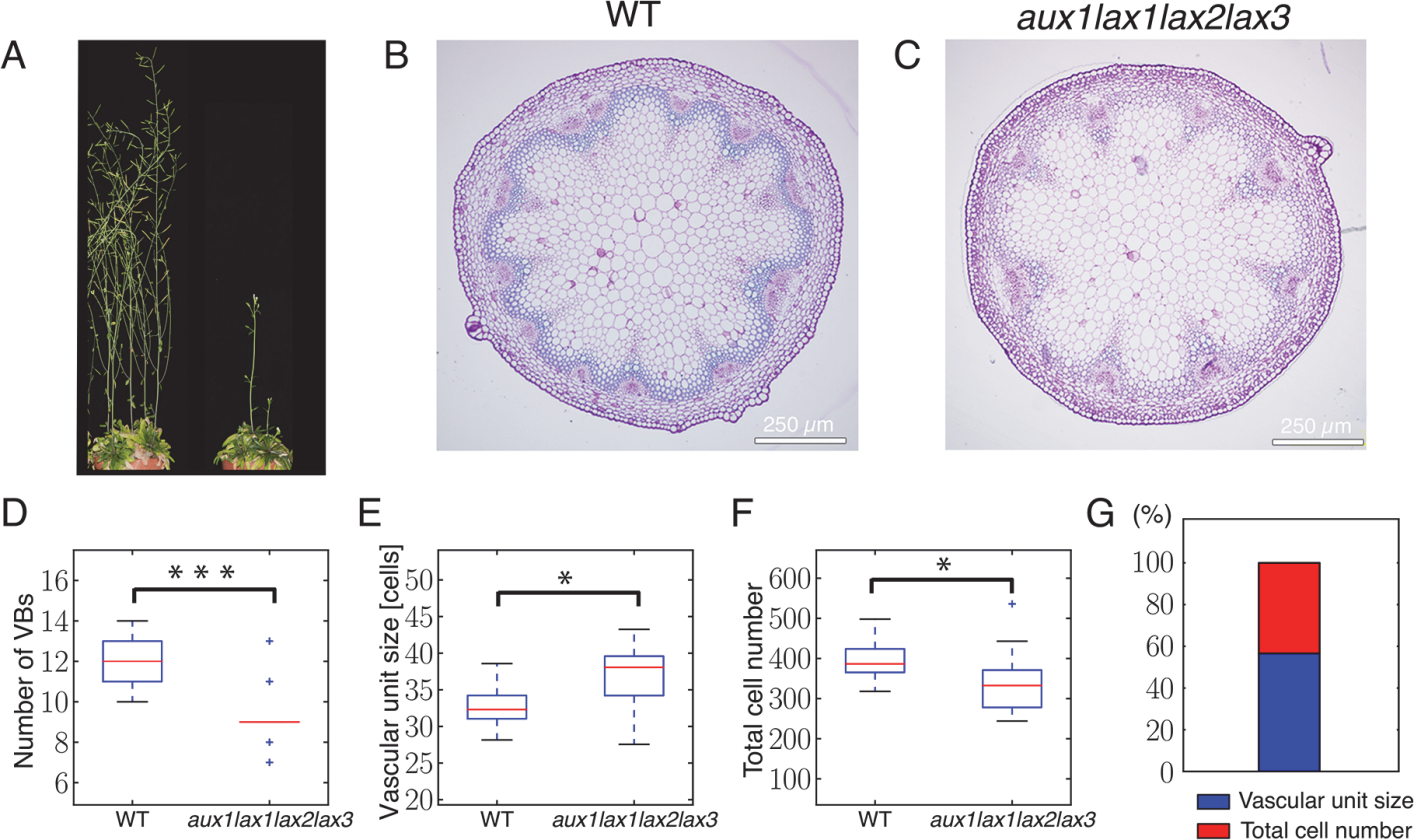
Auxin influx carrier quadruple mutants show fewer vascular bundles in the inflorescence stem due to an increased spacing of vascular bundles and to a decreased number of cells in the provascular ring. (A) WT 14-week-old plant (left) and *aux1lax1lax2lax3* quadruple mutant 14-week-old plant. (B) Basal shoot cross section of WT shoot inflorescence stem. (C) Basal shoot cross section of *aux1lax1lax2lax3* quadruple mutant shoot inflorescence stem. (D-F) Boxplots of VB number (D), vascular unit size (E), and total cell number across the provascular ring (F) for WT and *aux1lax1lax2lax3* mutant vascular rings. For the total cell number quantification along the shoot stem section, the ring of cells formed by the interfascicular fiber cells and the procambial cells within the vascular bundle were taken into account. The vascular unit size measures the spacing of VBs position and was defined as the number of procambial cells along the ring within a VB plus the number of interfascicular fiber cells up to the next VB in this ring. Note that the vascular unit size is enlarged in influx mutant plants, being consistent with the theoretical predictions shown in Fig 1. (G) Percentage of expected contribution of VB spacing (red) and total cell number (blue) to the change in VB number in the *aux1lax1lax2lax3* mutant, computed by using Eq 2. All plants were grown under short day conditions. In panels D-G, n = 24 for Col-0 and n = 18 for quadruple mutant plants. Scale bars: 250 μm. *: p-value≤ 0.01; ***: p-value≤ 0.0001.

We then analyzed radial sections at the basal region of the shoot inflorescence stem of mutants for auxin influx carriers [31] grown in short days. The depletion of all auxin influx carriers resulted in a significant reduction in the number of VBs as compared to the WT (Fig 2A–2D). Single *aux1* mutant showed no VB number phenotype whereas *aux1lax1lax2* triple mutant exhibited a similar phenotype than the quadruple (S6 and S7 Figs). These results are consistent with all AUX1/LAX family members being expressed in the shoot vasculature where they could play redundant functions. As previously reported in the context of phyllotaxis [31], the quadruple mutant exhibited higher phenotypic variability than the wild type (S8 Fig), supporting a stabilizing role for auxin influx carriers.

We next evaluated the number of cells involved in this pattern. To this end, we decomposed the vascular pattern of shoot cross-sections into vascular units (S1 Fig), each one of them constituted by the cells along a cell file in a VB and by the cells within the immediately adjacent interfascicular fibers [7]. The spacing of VBs was defined as the number of cells in the vascular unit, i.e. the vascular unit size. Our results show a significant decrease in the number of VBs and the total cell number accompanied by a larger spacing of VBs in terms of number of cells (Fig 2E and 2F). This enlargement of the spacing is in agreement with the role of influx carriers predicted by the mathematical model (Fig 1).

Since the reduction of VB number could be influenced by both the increase in VB spacing and the reduction of provascular cell number, we quantified the expected contribution of each of these two elements to this phenotype (Materials and Methods). Our results show that the increase in VB spacing in *aux1lax1lax2lax3* mutants can account for 57% of the change in VB number, while the decrease in total cell number explains the remaining 43% of the change in VB number (Fig 2G). In the triple *aux1lax1lax2* mutant, a similar trend is found (S7 Fig).

### Auxin influx mutants show impaired xylem differentiation

**Fig 3:**
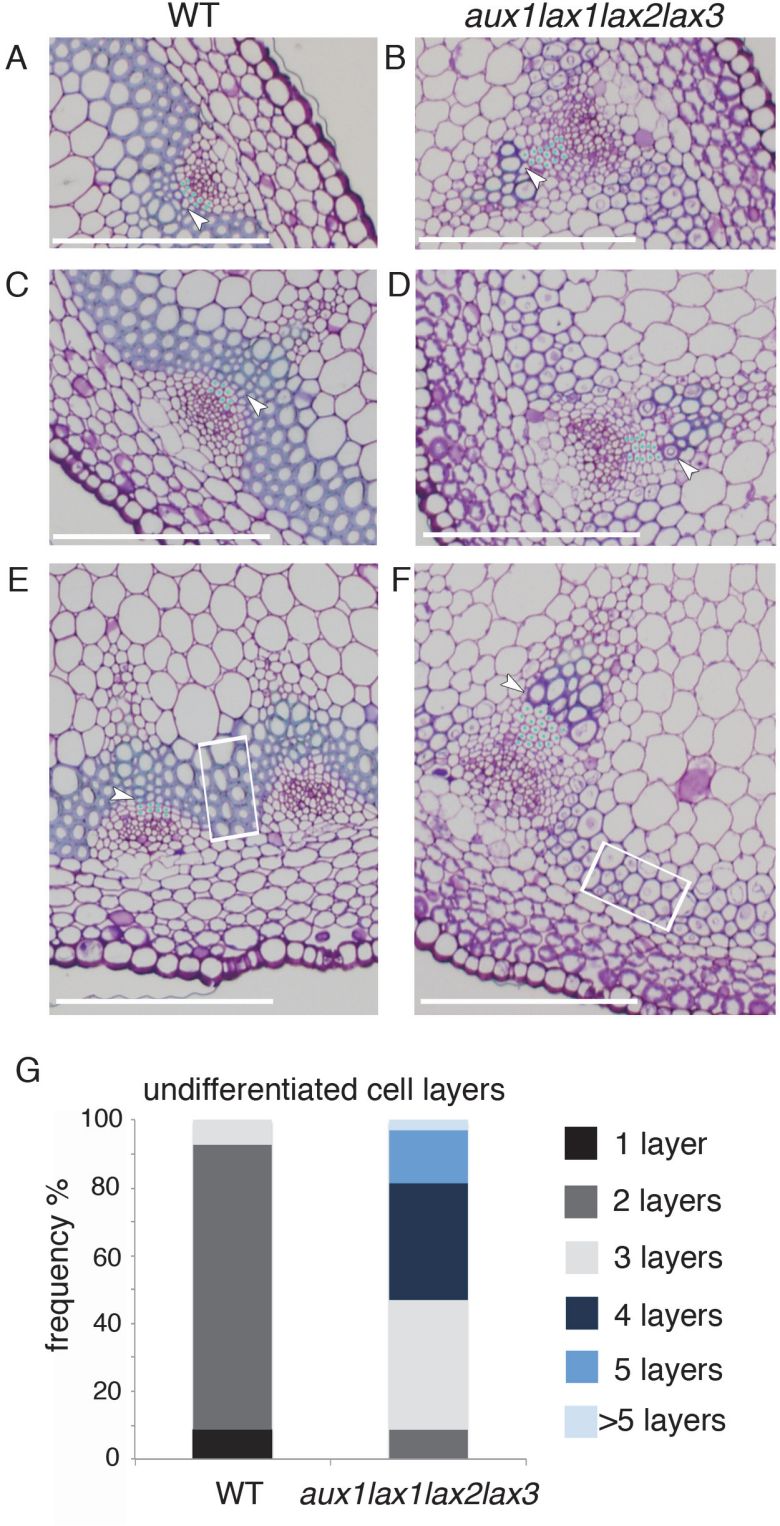
Influx carrier mutants show impaired xylem differentiation in Arabidopsis shoot stem. (A-B) VB magnification of a shoot basal cross section for WT (A, C and E) and *aux1lax1lax2lax3* quadruple mutant (B, D and F) 14-weeks old plants grown in short day conditions. Light blue dots indicate undifferentiated cell layers in procambium tissue between phloem and xylem differentiated cells. First differentiated xylem cell is indicated by white arrow. The undifferentiated cell layers comprise both the procambial cells and the meristematic xylem cells (round cells with undifferentiated walls between the procambium and the xylem). Note that above the procambial cells appear some undifferentiated cells with different shape than the procambial cells. This round shape is more characteristic from xylem cells while the cell walls are not differentiated. Therefore, we quantify them as undifferentiated cells, which can comprise both, procambial and meristematic xylem cells. White squares highlight interfascicular fiber cells. Scale bar: 100 μm. (G) Frequency distribution of the number of undifferentiated cell layers, for WT and *aux1lax1lax2lax3* mutants (n = 24 VB).

Besides the phenotype in the periodic patterning, the vascular differentiation was also impaired in the influx mutants. Compared to the WT, *aux1lax1lax2lax3* and *aux1lax1lax2* mutants clearly show a reduced differentiation of both the interfascicular fiber cells and of the xylem cells within the shoot VB (Fig 3A–3F). This impairment was accompanied by a significant increase and a higher variability in the number of undifferentiated cell layers within the VB of *aux1lax1lax2lax3* mutants, when compared to the WT (Fig 3A–3G). Together, these results uncover a role for auxin influx carriers in promoting xylem differentiation in the plant shoot.

To address whether the roles of influx carriers in periodic patterning and differentiation are independent, we investigated the vascular differentiation phenotype in tissues where vascular patterning is not periodic, such as the primary root. Histological analysis on the primary roots of *aux1lax1lax2lax3* mutants showed impaired xylem vessel differentiation (S9 Fig), and AUX1/LAX-VENUS lines revealed root cambium/xylem-specific expression (see Materials and Methods and S10 Fig), confirming that AUX1/LAX promote xylem differentiation independently of modulating periodic patterning.

**Fig 4:**
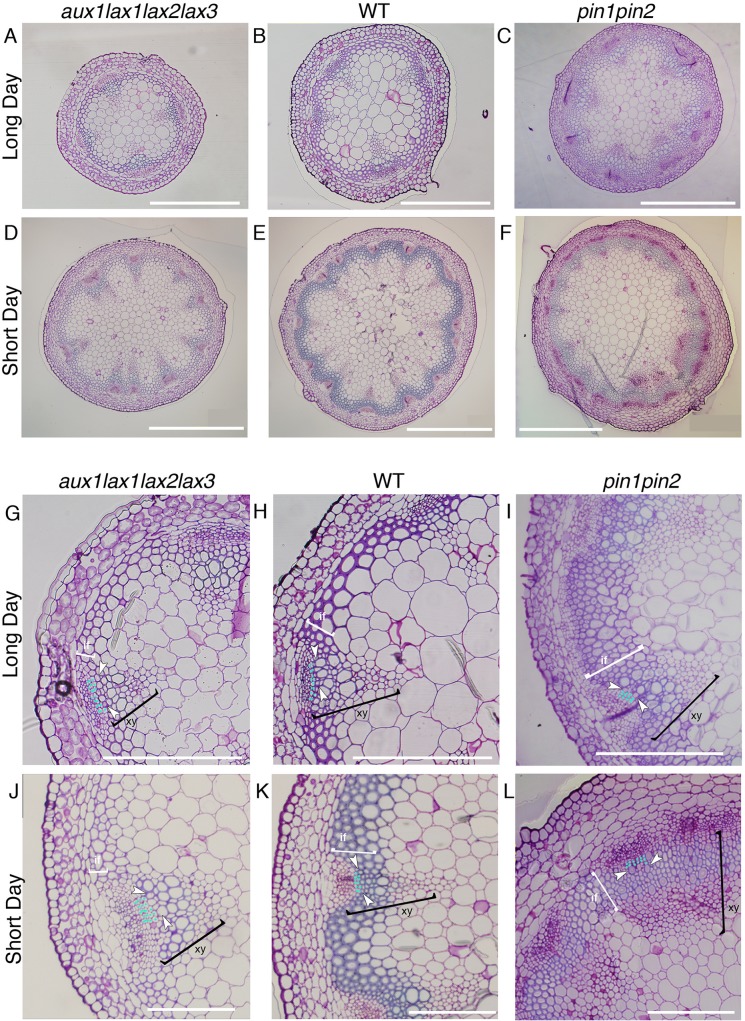
Vascular pattern and xylem differentiation phenotypes in long day and short day conditions for influx *aux1lax1lax2lax3* and efflux *pin1pin2* mutants. Basal shoot cross section of *aux1lax1lax2lax3* (A, D), WT (B, E) and *pin1pin2* (C, F) plants grown in long day conditions (A-C) and in short day conditions (D-E). WT and the *aux1lax1lax2lax3* plants grown in long day conditions showed no statistical significant differences in number of VBs, total cell number, nor average vascular unit size (S11 Fig, n = 18 for WT, n = 15 for *aux1lax1lax2lax3*). Scale bars: 500μm. VB detail of *aux1lax1lax2lax3* (G, J), WT (H, K) and *pin1pin2* (I, L) shoot inflorescence stem grown in long day conditions (G-I) and in short day conditions (J-L). Light blue dots indicate undifferentiated cell layers in procambium tissue between phloem and xylem differentiated cells. First differentiated xylem cell is indicated by white arrow. White brackets highlight the interfascicular fiber cells (if). Black brackets highlight the xylem cells (xy). Scale bars: 200 μm. Frequency distribution of the number of undifferentiated cell layers in long day conditions for the three genotypes is shown in S11 Fig.

Next, the vascular phenotype in the shoot stem of influx mutants grown in long day was analyzed, since in these light conditions no apparent vascular bundle number phenotype is seen in *aux1lax1lax2lax3* mutants (Figs 4A, 4B, 4D and 4E and S11), in agreement with the phyllotactic phenotypes described previously for these mutants in these conditions [31]. We found that the vascular differentiation was impaired in the quadruple mutants, albeit the phenotype was milder than in short day conditions (Figs 4G, 4H, 4J and 4K and S11). These results support that the role of AUX1/LAX in vascular differentiation is more prevalent than their role on modulating the vascular bundle number.

The role uncovered herein for the auxin influx carriers on xylem differentiation is opposed to that already described for efflux carriers [6] (Fig 4G–4L). In agreement, the efflux carrier mutant *pin1pin2* grown in short days shows increased xylem and interfascicular fiber differentiation similarly to what was shown in long days [7] (Fig 4C, 4F, 4I and 4L). Therefore, our results disclose that influx and efflux carriers have opposite roles in xylem cell differentiation.

### The effect of auxin influx carriers on extracellular auxin accumulation emulates the xylem differentiation phenotype

Since auxin signaling mediates vascular differentiation processes [16,46,57–59], we reasoned that alterations of auxin concentration mediated by influx carriers could result into impairment of auxin signaling, which in turn would drive defects in xylem differentiation and vascular patterning. To evaluate whether auxin signaling is impaired in influx mutants we analyzed the expression of the auxin response reporter DR5:GFP [60] in the *aux1lax1lax2* mutant backgrounds. The triple influx carrier mutant DR5:GFP *aux1lax1lax2* exhibited diminished auxin response, specifically at the VB, when compared to the DR5:GFP WT (S7 Fig). Since DR5:GFP reports the level of TIR1/AFB-mediated auxin signaling [61], these results point at a reduction of cytoplasmic auxin in the absence of influx carriers. We then turned into our modeling approach to unveil which of the multiple effects of influx carriers on auxin transport and distribution could underlie the control of xylem differentiation. To this end, we searched for those effects of influx carriers on auxin distribution that satisfy the restrictions we find in the xylem differentiation phenotype: the effect of influx carriers on xylem differentiation is (i) independent of the role on modulating the periodic pattern, (ii) is more pervasive than the modulation of the periodic pattern and (iii) is opposed to that of efflux carriers. Note that our mathematical analysis revealed that influx and efflux carriers do not necessarily drive antagonistic effects. For instance, both influx and efflux carriers promote periodic pattern formation (Figs 1 and S12) [2,3], faster patterning processes (S1 Video) [7], and stabilization of the pattern [40–42]. Therefore, the third condition (iii) also imposes a restriction on which effect of influx carriers controls xylem differentiation.

**Fig 5:**
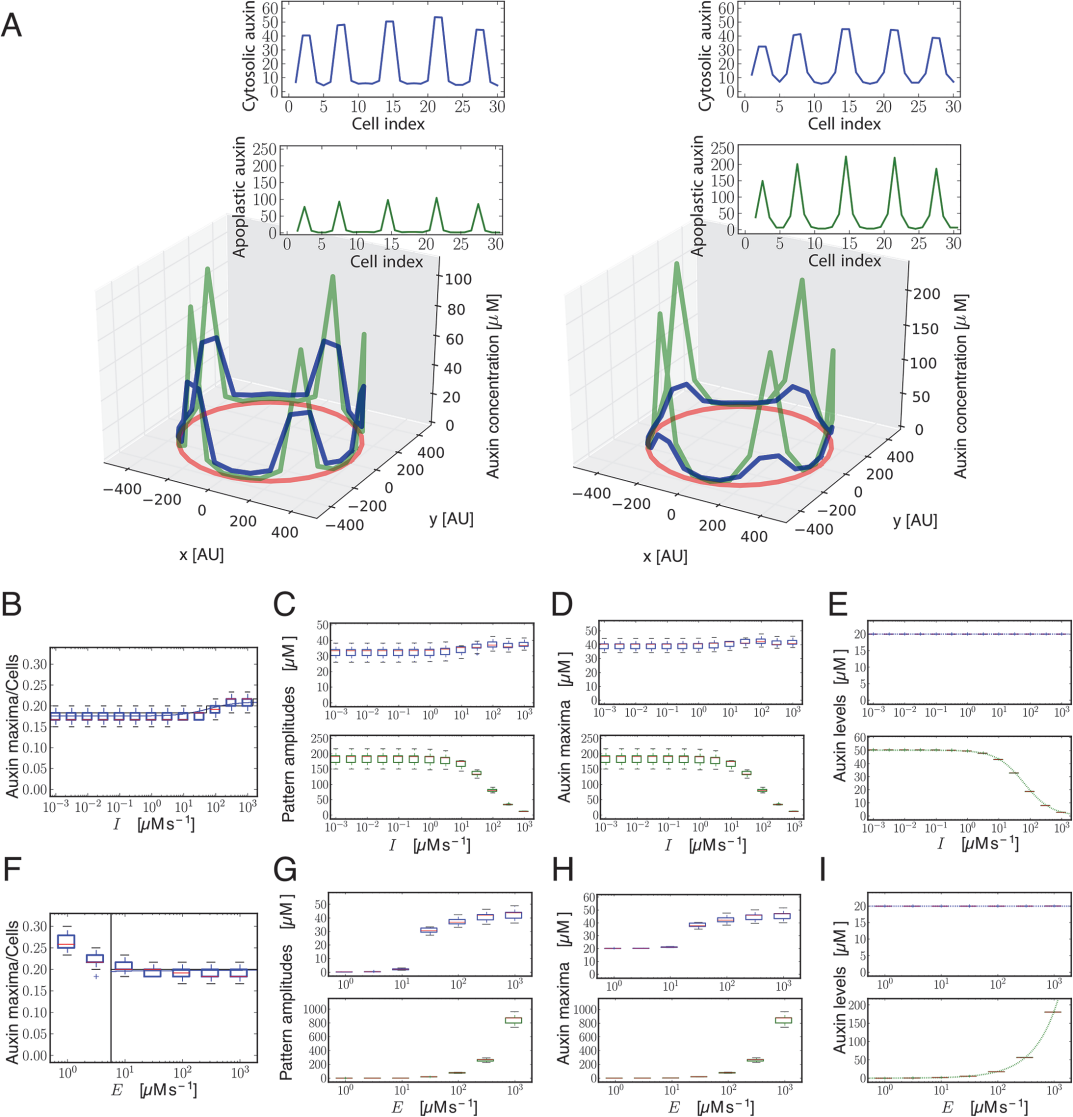
Modeling shows that influx carriers diminish differences in the concentration of auxin in the apoplast. (A) Snapshots of simulation results showing periodic distribution of auxin inside and outside cells for higher (left, *I* = 100 μM s^-1^) and lower (right, *I* = 0.001 μM s^-1^) influx carriers levels along a ring of vascular tissue composed of 30 cells surrounded by the apoplast. Cytosolic (blue) and apoplastic (green) auxin concentrations at time t = 17.5 are shown. The red circular line represents the ring of cells in the tissue. Insets depict the same results projected into a 2D plane. Space is represented in arbitrary units [AU]. The number of auxin maxima is the same in both cases. (B-E, F-I) Simulation results showing the number of cytosolic auxin maxima over the total number of cells (B,F), the amplitude of the pattern of auxin (C,G), the averaged auxin maxima levels (D,H) and the averaged auxin values along the vascular ring (E,I) in the cytosol (top panels, blue boxplots) and in the apoplast (bottom panels, green boxplots) as a function of the amount of influx carriers *I* (B-E) and of efflux carriers *E* (F-I). Each boxplot depicts the results for 30 simulations with different initial auxin distributions (Methods). Simulations in B-I were done for rings of 60 cells until time *t* = 17.5. Depicted boxplot components are the same as in Fig 1B. Other details of panels (B, F) are the same as in Fig 1B. Vertical line in (F) indicates the critical parameter value (in this case, the efflux carriers levels *E*) above which the pattern can not emerge, derived from linear stability analysis. Dotted lines in (E,I) panels correspond to the theoretical auxin homogeneous steady states given by Eqs S9 and S35. See Materials and Methods and S13 Fig for more details on the computation of the amplitude and average levels. All parameter values as in Fig 1 except for passive influx *D*_*ca*_ = 50 s^-1^. *E* = 105 μM s^-1^ for A-E panels, while *I* = 100 μM s^-1^ for F-I panels.

**Fig 6:**
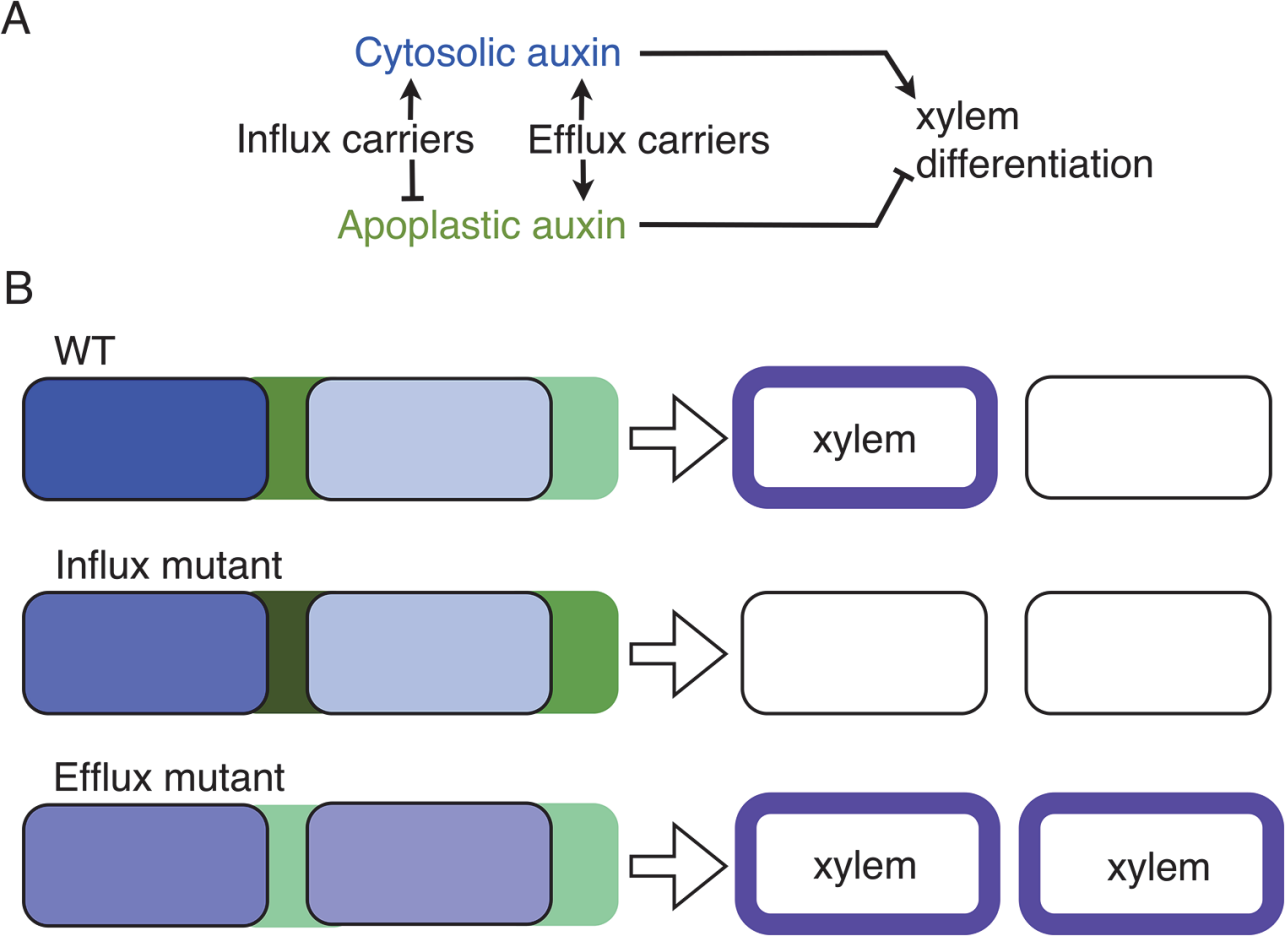
A model of apoplastic and cytoplasmic auxin control of xylem differentiation. (A) Auxin cytoplasmic signaling is required for xylem differentiation [16,46,57–59]. Our computational analysis predicts apoplastic (extracellular) auxin as an inhibitor of this differentiation. Both efflux and influx carriers increase cytoplasmic auxin concentration at auxin maxima, while they antagonistically regulate apoplastic auxin concentration. Arrows stand for activation, while blunt arrows for inhibition. (B) Cytoplasmic (blue) and apoplastic (green) auxin concentrations in two cells (rectangles with rounded corners). Lighter blue and green account for decreased cytoplasmic and apoplastic concentrations respectively. The predicted differentiation phenotypes of WT, influx and efflux mutants are depicted on the right. Xylem differentiated cells are depicted with blue borders.

The analysis showed that influx carriers increase cytosolic auxin in those cells harboring the auxin maxima (Figs 1A and S13), in agreement with the reduced response exhibited by DR5:GFP in the auxin maxima of the triple *aux1lax1lax2* mutant background (S7 Fig). In addition, influx carriers tend to reduce spatial differences in the concentration of apoplastic auxin (what we call pattern amplitude of the apoplastic auxin) as well as to diminish, as expected [51], the apoplastic auxin concentration (S13 Fig). Importantly, this role influx carriers have on apoplastic auxin concentration satisfies the three conditions described above (i-iii). This role is more pervasive than that on modulating the pattern periodicity (Figs 5 and S14). Moreover, it is independent of the modulation of the periodicity, since changes of the periodicity of the pattern do not require changes in the concentration of extracellular auxin (S15 Fig). In addition, it is opposed to the effect driven by efflux carriers, which tend to increase the differences of apoplastic auxin across the ring of cells and to increase the apoplastic auxin concentration (Figs 5 and S13 and S14, Materials and Methods). While extracellular auxin is dependent on influx carriers such that the three conditions (i-iii) above hold, cytosolic auxin is not. Cytosolic auxin is promoted both by influx and efflux carriers at the cells harboring auxin maxima (Figs 5 and S13 and S14). Taken together, our computational analysis supports that influx carriers promote cytoplasmic auxin signaling and thereby xylem differentiation and proposes that auxin signaling may respond as well to changes in extracellular apoplastic auxin concentration driven by influx carriers to control vascular differentiation (Fig 6).

## Discussion

Mathematical modeling has recently emerged as an effective discipline to characterize auxin patterns and the processes driven by them, like vascular patterning in the root [1–4,9,10,40,42,55,62]. From these studies it is known that auxin-dependent polarization of efflux carriers can drive periodic patterning [3,4,55]. Our mathematical model predicts that auxin influx carriers, despite not being polarized in our model, can modulate the periodicity of the auxin pattern. The observed changes in the vascular pattern periodicity in the shoots of influx mutants are in agreement with the model prediction. Moreover, our model shows that influx carriers can facilitate periodic patterning. Intuitively, the roles of auxin influx carriers in patterning disclosed in this study can be understood from the competition between polar transport and apoplastic diffusion. Polar transport is at its most effective mode when the auxin expelled from the cytosol is able to reach only the adjacent cells and not cells located further away [51]. Efficient uptake of auxin by influx carriers facilitates that this happens.

Early mathematical models proposed that influx carriers stabilize phyllotactic patterning [40–42]. Recently, it has been shown that influx carriers can have additional roles in patterning, such as setting which root cells have high levels of auxin [63]. The role of influx carriers on promoting auxin maxima and facilitating periodic patterning may have previously remained unnoticed in theoretical analyses because either apoplastic diffusion and/or auxin-induced synthesis of carriers were not taken into account. These novel roles are uncovered through modeling only when either one or both elements are included (S1 Text). The inclusion of apoplastic diffusion in the model revealed another interesting aspect of auxin periodic patterning: efflux carriers do not modulate the periodicity of patterns as influx carriers do (S12 Fig). Therefore, this suggests that the distorted vascular bundle phenotypes in efflux mutants are not because of modulation of the periodicity, and may arise from the strong slowing down of the auxin transport dynamics as previously proposed [7]. In addition, future 3D modeling approaches that take into account the connectivity of the vascular strands with the phyllotactic pattern [64] will help in a better understanding of the phyllotactic and vascular pattern formation.

Based on our modeling results, it is tempting to speculate what in auxin transport is distinct between short day and long day conditions that can explain the differences we found in phenotypes (Fig 4), namely, that quadruple influx carrier mutants in long day only show reduced xylem differentiation and not a vascular bundle phenotype. Assuming the xylem differentiation scheme of Fig 6, the model indicates that long day conditions could be mimicked by (1) the absence of auxin-induced synthesis of carriers together with lower auxin apoplastic diffusion coefficients than in short day (S14 Fig), or by (2) an increase of the ratio of passive influx transport across the cell membrane over the active one when compared to short day conditions (Fig 5). We found that carriers are localized at auxin maxima in long day conditions (S16 Fig), supporting auxin-induced synthesis for this photoperiodic condition and discarding the first scenario. Regarding the second scenario, the model shows that the concentration of apoplastic auxin is lower when the passive influx transport across cell membranes increases (Figs 5 and S13 and S1 Text). This predicts that the influx carriers mutant plants should display a milder differentiation phenotype in long days than in short days. This prediction is in agreement with the phenotypes exhibited by *aux1lax1lax2lax3* mutant shoots inflorescence stems (Figs 3 and 4 and S11). It is worth stressing that predicting these differences between the differentiation phenotypes in long day and short day conditions of influx carriers mutants is restrictive. For instance, no difference of differentiation phenotype is expected if long day conditions corresponded just to lower active influx transport than in short days, while the opposite difference is predicted by a photoperiod-dependent change of apoplastic auxin diffusion (S14 Fig). Based on this analysis, we may hypothesize that the photoperiod could change the balance between passive and active influx transport across the cell membrane, being passive influx more relevant at long day conditions than at short days. Yet, in both conditions, active influx transport is expected to be more important than passive entrance into the cells. Passive auxin entrance into the cells could increase in long days by increasing the cell membrane permeability, for instance. In addition, the amount of active influx carriers may decrease in long days as well. Potentially, active influx transport could be modified by the photoperiod through light-modulation of intracellular trafficking [65,66].

In summary, by combining experimental and theoretical approaches, we propose novel roles for auxin influx carriers in vascular patterning and differentiation during plant development. By assuming that auxin maxima position VBs [7], we evaluated the role of auxin influx carriers in the periodic patterning of auxin maxima in the Arabidopsis shoot inflorescence stem. *auxlax1lax2lax3* mutants showed a reduction in VB number in the shoot stem involving both an increase in the spacing of the pattern and a reduction in the total number of cells along the provascular ring. This increase in the spacing can be explained by the role of influx carriers predicted by our modeling approach. Moreover, the quadruple *auxlax1lax2lax3* mutants also depicted decreased xylem differentiation. Analysis of shoot and root phenotypes indicates a pervasive role of auxin influx carriers in promoting differentiation of xylem cells that is independent on their role on periodic vascular patterning. Our data support the established idea that TIR1/AFB-mediated auxin signaling, operating in cytoplasm and nucleus, is required for xylem differentiation [15,16,46,47]. In addition, our computational analysis predicts that extracellular auxin can be sensed from the apoplastic space and inhibit xylem cell differentiation (Fig 6). While no direct empirical evidence for apoplastic auxin signalling controlling xylem differentiation is described, it has been reported that apoplastic auxin can be sensed by the cell surface receptor ABP1-TMK and drive the downstream auxin signaling [67]. Furthermore, the potential connection between apoplastic and cytosolic auxin signaling pathways has been also shown [67]. However, very recent evidence has challenged the role of ABP1 as auxin signalling component [68]. Further work will contribute to unravel the molecular mechanism that drives these phenotypes as well as to understand the impact of environmental conditions on auxin-driven patterning.

## Materials and Methods

### Mathematical modeling

We used the mathematical model of polar auxin transport by [3] in the form it is presented in [41] with the inclusion of apoplastic auxin transport as in [7] (S2 Fig). Given that auxin diffuses fast in the cytosol [69], we considered for simplification auxin concentration homogeneously distributed inside the cell, what would be consistent with instantaneous diffusion of auxin across the cytosol. In the model, auxin is pumped into the cell through the influx carriers, which are homogeneously distributed in the cell membrane. Moreover, auxin is pumped out of the cell to the apoplast through the efflux carriers, which can be polarly distributed in the cells. We considered efflux carriers’ localization to depend on the concentration of auxin in neighboring cells and, for simplicity, to be at equilibrium, as done in [3,40,41]. The model takes into account that a constant fraction of the auxin (set by the pH condition) is protonated and is passively transported into the cells as in [3]. Auxin production and degradation is set to occur inside cells. In the model, we refer to cytosolic and apoplastic auxin as the auxin inside and outside the cell, respectively.

The dimensional model equations for cytosolic auxin concentration in cell *i* and apoplastic auxin concentration in the apoplastic compartment *i* (see scheme in S2 Fig) read

$$\begin{array}{c} \frac{dA_{i}}{d\tau }=-\sum_{j\in n(i)}W_{ij}J_{ij}-\nu_{c}A_{i}+\sigma_{c}\\ \frac{da_{i}}{d\tau }=\sum_{j\in N(i)}W_{ji}\frac{V_{cell}}{V_{ap}}J_{ji}+D_{w}\nabla_{i}^{2}a_{i} \end{array}\;,$$ (1)

with τ being time, *D*_*w*_ the apoplastic diffusion coefficient, σ_*c*_ and v_*c*_ the auxin production and degradation rates, *V*_*cell*_ the cell volume, *V*_*ap*_ the apoplast volume, *W*_*ij*_ the ratio between the contact area of cell *i* with apoplast *j* and the cell volume. ▽_i_^2^ is the discrete Laplacian. *J*_*ij*_ stands for the auxin flux from the cell *i* to the apoplast *j* and contains the protonated passive auxin transport, and both active transports due to the influx and efflux carriers as described in [41] (see S1 Text for details). Active influx transport was simplified to drive only entrance of anionic auxin into the cell. Analogously, active efflux transport was set to mediate only the exit of anionic auxin from the cell. For simplicity and with the aim of focusing in the linear regime of the dynamics, we considered linear auxin fluxes for both passive and active transports (S1 Text).

For convenience, we analyzed the model in nondimensionalized time units (S1 Text). The resulting model parameters can be related to physico-chemical magnitudes that have been measured or can be estimated, even though we expect having robust behaviors that are not very dependent on parameter values. In our study, we chose to mainly vary the following effective dimensional parameters: the influx parameter *I*, the apoplastic diffusion coefficient *D* and the efflux parameter *E*. Our simplified geometry corresponds to a line of cells with periodic boundary conditions and no cell division was included.

### Simulation details

We integrated the dynamical model for auxin transport (Eqs S7-S8) through a Runge-Kutta method of 4^th^ order [70] with time step d*t* = 0.0001 being *t* the non-dimensional time. Most of the parameter values were set according to already published work, some of it based on experimental data (S1 Table). Unless otherwise stated, we set as initial conditions the homogeneous solution with small variability. We integrated the dynamics until a fixed time point (*t* = 17.5). This time point was chosen such that a periodic pattern of auxin maxima was established for the parameter values of the normal conditions (Fig 1A) but was still incipient at efflux mutant conditions, yielding distorted patterns for this mutant (S12 Fig and S1 Text).

For each pattern at time t = 17.5, we quantified the ratio of the number of cytosolic auxin maxima over the number N of cells in the simulated provascular ring. For a periodic pattern, this provides a measurement of the characteristic wavenumber. Note that the inverse of this quantity is the average number of cells between two auxin maxima (i.e. the characteristic wavelength or spacing). In the simulations, we observed very incipient maxima much smaller than the rest. We omitted such incipient maxima for the quantification of maxima spacing, the cytosolic and apoplastic auxin average maxima and pattern amplitudes. We determined that a cytosolic (or apoplastic) auxin maxima was incipient at the end of any given simulation when the difference of its cytosolic (or apoplastic) auxin value with the average cytosolic (or apoplastic) minima in such single simulation was less than 0.15 times the cytosolic (or apoplastic) auxin pattern amplitude in such simulation. By omitting these maxima, which arise especially at high influx levels (see incipient maxima near cell 60 in left panel of Fig 1A), we have seen that the numerical simulations are in better agreement with the theoretical prediction for the periodicity of the pattern, especially at higher influx levels.

Simulations were performed with custom-made programs written in Fortran77 and in C++. We provide a code written in Mathematica [version 9.0, Wolfram Research; code provided in pdf and Mathematical notebook format (.nb)] that can be used by the reader to test how influx carriers and other model parameters affect to the pattern formation process (see S1 Code).

### Theoretical prediction of the emerging pattern

In pattern formation studies, linear stability analysis enables the prediction of the characteristic wavelength of the emerging patterns [71], and it has already been used for mathematical auxin transport models (see for instance [3,40,41]). We performed linear stability analysis over the stationary homogeneous state [71] (details are found in S1 Text). This analysis provides theoretical predictions on which parameter values can drive periodic pattern formation and on how the periodic pattern that starts to be formed depends on the parameter values. Accordingly, through this analysis we extracted predictions on how the characteristic wavenumber (*κ*) (i.e. the number of periodic maxima over the total number of cells) depends on the model parameters (see S1 Text for details).

### Plant material and growth conditions

All the mutant plants analyzed here were in *Arabidopsis thaliana* Columbia-0 (Col-0) ecotype background. Seeds for DR5:GFP in *aux1lax1lax2* and Col-0 WT backgrounds and *auxlax1lax2lax3* were described elsewhere [31]. Seeds were surface-sterilized in 35% sodium hypochlorite, vernalized at 4°C for 48h, and germinated on plates containing 1x Murashige and Skoog (MS) medium. Seedlings were grown for 10 days on plates under short day photoperiod (8h light / 16h dark; 8470 lux; 20–23°C) and then transplanted to soil and grown under the same conditions for 14 weeks. The main shoot inflorescence stem was cut at approximately 1 cm above the rosette for sectioning and further histological analysis. Short day conditions can occasionally drive the emergence of aerial rosettes in both WT and *aux1lax1lax2lax3* adult plants. Plants that showed these aerial rosettes development were not considered for our analysis.

The VENUS fluorescent protein [72] fusions of AUX1/LAX proteins were generated by a recombineering approach [73] and have been described before for AUX1 [63]; LAX1 and LAX2 [25]. In brief, VENUS was fused in frame after the codon 116 for AUX1; 122 for LAX1; 110 for LAX2 and 114 for LAX3 to create respective AUX1/LAX fluorescent protein constructs (P*roAUX1*:*AUX1-VENUS*; *ProLAX1*:*LAX1-VENUS*; *ProLAX2*:*LAX2-VENUS* and *ProLAX3*:*LAX3-VENUS*). Transformation of Agrobacterium (C58) and Arabidopsis was done as described before [74]. Transgene-specific cDNA sequences of these lines were PCR-amplified and sequenced to ensure against rearrangements of the transgenes.

### Histology and microscopy

Inflorescence stem sections from both WT and mutant Arabidopsis plants were fixed at 4°C overnight in 1.25% glutaraldehyde in 0.1 M sodium cacodylate buffer (pH 7.4). Samples were dehydrated through a graded series of ethanol (30%, 50%, 70%, 90% and 100%; 45 min each one) and then infiltrated in 1:1 Historesin-I (Technkovit):ethanol for 30 min at room temperature, followed by 100% Historesin-I 100% at 4°C overnight. Blocks were prepared by placing samples into plastic molds, which were filled with 100% Historesin-II (Technkovit). Each mold was covered with parafilm and kept overnight at 4°C to accelerate their solidification. Historesin-I and II were prepared following the manufacturer’s instructions. Transverse stem sections (3 μm) were obtained with a Leica Microtome (Microtome RM2265, Leica). Sections were stained with 0.1% Toluidine blue in 0.1M NaPO_4_ pH7.0, rinsed and mounted in water for microscopical visualization in an Axiophot Microscope (Zeiss). GFP-fluorescence was observed in hand-made sections from the same part of the stem in a stereomicroscope (SZX16, Olympus). VENUS fluorescence lines (same part of the stem as described above) grown in short day conditions were incubated for 1–2 hours in 4% para-formaldehyde in PBS under vacuum conditions. After three washes with PBS, the samples were mounted in a hand-made block of 4% agarose and 0.01% Triton X-100 in PBS (pH 7.2–7.4). 150 μm sections were cut in a Micron HM650V vibratome and analyzed in a Leica TCS SP5II HCS A confocal microscope (Leica). Kr/Ar 488 laser was used with an excitation wavelength of 514 nm and detected an emission window of 525–569 nm for the VENUS/YFP. An excitation wavelength of 405 nm and an emission window of 434–483 nm were used for the blue autofluorescence of the xylem.

For the root histological studies, seedlings were grown on soil for 5 weeks on long day photoperiods (16h light/ 8h dark; 23°C). The root samples were embedded in Historesin (Leica) as described in [75], and 5–10 μm sections were cut approximately 5 mm below the hypocotyl. The sections were stained with toluidine blue and only the vessel elements in the primary phase of secondary xylem development were quantified with ImageJ. Vessels formed during the secondary phase were not quantified, since fibers with thick cell walls are formed then [76], thus making it difficult to distinguish the vessel elements from fibers.

### Quantitative vascular analysis

Quantification of all the vascular parameters (stem diameters, number of cells, interfascicular fiber length and number of undifferentiated cell layers) was performed manually or using ImageJ software (http://rsb.info.nih.gov/ij/). Vascular bundles and cells were manually counted from microscope images. To determine whether the WT samples were statistically significant with respect to mutant samples, we performed the Wilcoxon rank sum test with Matlab. When we performed the test on the vascular unit sizes of WT samples against those of mutant samples, we chose the average vascular unit size per plant as the tested variable, but we have confirmed that the test results were very similar if we took the median of the vascular unit size per plants. Plots were performed with Python 2.7 by means of the Matplotlib package and with Excel. Quantification of the contribution of VB spacing (characteristic wavelength, *λ*) and total cell number (*N*) to the change in VB number (*V*) was computed through

$$\frac{\Delta V}{V_{WT}}=\frac{\Delta \lambda }{\lambda_{WT}}+\frac{\Delta N}{N_{WT}}\;,$$ (2)

where Δ*X* = *X*_*mu*tan *t*_−*X*_*WT*_ with *X* being the median value found for each variable. This relation stems from *V* = *N* / *λ*. The percentage of contribution of VB spacing is then 100Δ*λV*_*WT*_ / (Δ*V λ*_*WT*_).

## Supporting Information

S1 Fig Vascular tissue organization in the Arabidopsis shoot inflorescence stem. (A) Magnification of a shoot basal cross section for a 5-week-old Arabidopsis WT plant. Grey arrowheads indicate the beginning and the end of the procambial cells layers within a VB. Procambial cells are depicted in grey. Green arrowheads indicate phloem cells. Dark blue arrowheads show the xylem cells in the VB. Light blue arrowheads show the IF cells (B) Cartoon of the WT plant represented in (A) where procambial cells are depicted in grey, phloem cells are depicted in green, VB xylem cells in dark blue and IF cells in light blue. Red line indicates the length of one vascular unit formed by one VB and their immediate IF cells.
(TIF)

S2 Fig Polar auxin transport and modeling scheme. (A) Chemiosmotic model for auxin transport. Auxin can be in its protonated or anionic form, (IAAH and IAA^-^, respectively). Red arrows represent the auxin flux driven by PIN efflux carriers, which are asymmetrically localized on the membrane; yellow arrows represent the auxin flux driven by AUX1/LAX influx carriers. Orange arrows denote passive entrance of auxin into the cell. Being auxin a weak acid, once it enters the cells, where the pH is less acidic than in the apoplast, it gets deprotonated and, consequently, trapped inside. Therefore, auxin can only exit via the action of efflux carriers, such as PINs, which have a polarized localization on the membrane, conferring directionality to auxin transport. (B) Modeling scheme illustrating the cellular ("cell") and apoplastic ("ap.") spaces, and the cycling of the efflux carriers within cells. The labeling (*i*) of cells and apoplasts used in the mathematical equations is also indicated. We model the apoplast as a compartment between cells, and we set effective auxin apoplastic diffusion between the two apoplasts that are adjacent to a cell (e.g. the apoplasts adjacent to cell *i* are apoplasts *i*-1 and *i*; extracellular auxin can diffuse then from apoplast *i* to apoplast *i*-1 and vice versa). Efflux carriers are asymmetrically distributed in the cell membrane since their cycling rates to the different membrane segments in a cell are also asymmetric. Influx carriers are symmetrically distributed throughout the cell membrane.
(TIF)

S3 FigNumerical simulation results show the distribution of auxin carriers.For the parameter values of Fig 1A with higher (left, *I* = 100 μM s^-1^) and lower (right, *I* = 0.001 μM s^-1^) influx carriers levels, we show the distribution of influx (solid black line) and efflux (dashed gray line) carriers together with cytosolic auxin (blue line). The levels of carriers is normalized to 1/2 for the influx and 1 for the efflux, and corresponds to $I_{T}(A_{i})=\frac{1}{2}\frac{A_{i}}{\theta_{I}+A_{i}}$ and $P_{T}(A_{i})=\frac{A_{i}}{\theta_{P}+A_{i}}$ respectively (see S1 Text). Cytosolic auxin has been normalized to 1.(TIF)

S4 Fig Influx carriers facilitate and modulate periodic patterning in a simplified scenario with the synthesis of carriers being independent of auxin. The results correspond to a scenario with constant total amount of carriers per cell (no auxin-induced synthesis of carriers, *θ*_*I*_ = *θ*_*P*_ = *0* μM). Panels A-C as in Fig 1. (A) Snapshots of simulation results showing periodic distribution of auxin inside and outside cells for higher (left, *I* = 100 μM s^-1^) and lower (right, *I* = 0.001 μM s^-1^) influx carriers levels along a ring of vascular tissue composed of 60 cells surrounded by the apoplast. Cytosolic (blue) and apoplastic (green) auxin concentrations at time t = 17.5 are shown. The red circular line represents the ring of cells in the tissue. Insets depict the same results projected into a 2D plane. Space is represented in arbitrary units [AU]. (B) Simulation (boxplot) and theoretical estimation (*κ*, depicted by solid lines) results of the inverse value of the number of cells between cytosolic auxin maxima at different influx levels (*I*) for *D* = 2 s^-1^. Each boxplot depicts the results for 30 simulations with different initial auxin distributions (Materials and Methods). Simulations were done for rings of 60 cells. Depicted boxplot components are the same as in Fig 1B. Crosses represent outliers. The theoretical estimation is performed through linear stability analysis for a ring of 60 and 1200 cells (black and blue solid lines, respectively). The dashed light blue line is obtained from the analytical expression in S1 Text (Eqs S32-S33). (C) Phase diagram obtained from theoretical linear stability analysis on a ring of 60 cells in the parameter space of influx parameter (*I*) and apoplastic diffusion parameter (*D*). The solid line divides the space in two regions, as in Fig 1C. Above the solid line the homogeneous state is linearly stable and no periodic pattern can be formed from small perturbations of it. Below the solid line, the homogeneous state is linearly unstable and a periodic pattern can arise from it. The dashed black line is obtained from the analytical expression in S1 Text (Eq S34). The color scale shows the theoretical estimation of the inverse value of the average number of cells between cytosolic auxin maxima (*κ*). The results shown in A, B and C in this simplified scenario with constant total levels of carriers in the cell are qualitatively very similar to those shown in Fig 1 (which include auxin-induced synthesis of carriers). The main difference being that for constant levels of carriers the dependence of *κ* on influx carriers is less accentuated. In addition, the analytical expressions (Eqs S32-S34 in S1 Text, dashed lines in panels B and C) extracted for this simplified model are in very good agreement with the exact theoretical computations (solid lines in panels B and C) and hence are useful to predict the dependence of pattern formation features on parameter values (S1 Text). Parameter values as in Fig 1 except for the synthesis of carriers which is given by *θ*_*I*_ = *θ*_*P*_ = *0* μM.
(TIF)

S5 Fig Localization patterns of the auxin influx carrier proteins in the Arabidopsis shoot inflorescence stem in short day conditions. AUX1/LAX-VENUS reporters show localization in procambial, protoxylem and phloem cell files in the vascular bundles of Arabidopsis shoot stems. (A,B) *ProAUX1*:*AUX1*::*VENUS* fluorescence is present in procambial and protoxylem cell files. (C,D) *ProLAX1*:*LAX1*::*VENUS* fluorescence is present in procambial and protoxylem cells. (E,F) *ProLAX2*:*LAX2*::*VENUS* fluorescence is present in procambial cells. (G,H) *ProLAX3*:*LAX3*::*VENUS* fluorescence is present in procambial and in the phloem cell files. Blue autofluorescence highlights xylem cells and interfasciular fibers. Pink arrowheads indicate protoxylem cells within the VB. White arrows indicate undifferentiated procambial cells between phloem and xylem cells. Phloem cells are indicated by green arrowheads. All plants were grown for 7–11 weeks in short day conditions. Images were collected from cross sections at the basal part of the shoot inflorescence stem. Scale bars: 100 μm.
(TIF)

S6 Fig Phenotype of *aux1* single mutant. (A,C) Shoot inflorescence stems for WT 5-weeks-old plant (A) and *aux1* mutant 5-week-old plant (C), grown in long day conditions **(**B,D) Shoot inflorescence stems for WT 14-weeks-old plant (B) and *aux1* mutant 14-week-old plant (D), grown in short day conditions. Scale bars: 250 μm. p-values of the VB numbers of WT versus *aux1* mutants are 0.56 in short day conditions (n = 12 for WT and n = 12 for *aux1* mutants) and 1.0 in long day conditions (n = 6 for WT and n = 6 for *aux1* mutants), what shows no statistical difference.
(TIF)

S7 Fig Auxin influx carrier triple mutants show less vascular bundles and reduced auxin response when compared to WT DR5:GFP plants. (A) WT DR5:GFP 14-weeks-old plant (left) and *aux1lax1lax2* DR5:GFP triple mutant 14-week-old plant (right), grown in short day conditions. (B) Basal shoot cross section of DR5:GFP in Col-0 WT background. (C) Basal shoot cross section of DR5:GFP in *aux1lax1lax2* triple mutant background. (D-F) Boxplots of VB number (D), vascular unit (cells/VB) (E) and total cell number (F) for WT DR5:GFP and *aux1lax1lax2* DR5:GFP mutant. For the total cell number quantification along the shoot stem section, the ring of cells formed by the interfascicular fiber cells and the procambial cells within the vascular bundle were taken into account. (G) Percentage of contribution of VB spacing and total cell number on the change in VB number in the *aux1lax1lax2* DR5:GFP mutant. The VB spacing (p-value = 0.057) and the total cell number (p-value = 0.124) show the same trends as in the quadruple, but they are not statistically significantly altered. This suggests that despite neither of these two trends is statistically significant on its own, together they drive the significant change in VB number (p-value = 0.001). Moreover, the contribution of each trend to the reduction in VB number in the triple mutants is as marked as in the quadruple mutants (VB spacing can explain 61% of the change in VB number). (H, I) Cross section GFP fluorescence of (H) WT DR5:GFP and (I) *aux1lax1lax2* DR5:GFP plants. (Right panels) 3D density plots showing the GFP intensities in GFP fluorescence of (H) WT DR5:GFP and (I) *aux1lax1lax2* DR5:GFP plants. For facilitating the comparison between right panels in H and I, both 3D plots have been colored according the fluorescence levels in arbitrary units, following the same color scale. (J,K) Left panels show VB magnification of a shoot basal cross section for WT DR5:GFP (J) and *aux1lax1lax2* DR5:GFP mutant (K). (J, K) Right panels show VB magnification GFP fluorescence of (J) WT DR5:GFP and (K) *aux1lax1lax2* DR5:GFP plants. All plants were grown under short day conditions. Panels (D-G) show the analysis for n = 11 WT DR5:GFP plants and n = 9 for *aux1lax1lax2* DR5:GFP triple mutant plants. Scale bars: 250 μm. **: p-value ≤ 0.001.
(TIF)

S8 Fig Influx mutant plants exhibit higher variability of the shoot vascular pattern. (A) WT 14-weeks-old plants. (B) *aux1lax1lax2lax3* mutant 14-weeks-old plants. Note that *aux1lax1lax2lax3* quadruple mutants adult plants display shorter and more diverse stems than WT plants. (C) Basal shoot cross section of six independent WT plants. (D) Basal shoot cross section of six independent *aux1lax1lax2lax3* quadruple mutant plants. All the plants were grown in short day conditions.
(TIF)

S9 Fig Vessel element differentiation is impaired in the auxin influx carrier mutants roots. (A-E) Root cross sections for WT (A-B) and *aux1lax1lax2lax3* quadruple mutant (C-E). The quadruple mutant showed either defects in vessel differentiation (C, D) or a phenotype that resembled the WT (E). The frequencies of the observed phenotypes are indicated in the images (number of roots showing the phenotype/number of roots analyzed). Arrows: most recent vessel element. Square brackets: cambial region. Red rectangle: primary phase of secondary xylem development. Green rectangle: secondary phase of secondary xylem development. (F) Vessel element area in WT (left column; n = 559 from 25 plants) and *aux1lax1lax2lax3* quadruple mutant (right column; n = 640 from 18 plants) measured in the primary phase of secondary xylem development. p-value ≤ 0.001, Mann-Whitney test. Data represent the average ± 95% CI. Scale bars: 100 μm.
(TIF)

S10 Fig Auxin influx carriers are localized to the vascular tissues of the mature root. The AUX1/LAX reporter lines *proAUX1*:*AUX1-VENUS* (A,B), *proLAX1*:*LAX1-VENUS* (C,D), *proLAX2*:*LAX2-VENUS* (E,F), *proLAX3*:*LAX3-VENUS* (G,H) are localized to cambium and differentiating xylem in roots. (I,J) WT *DR5*::*GFP* plants show expression in differentiating xylem of the root cambium. The expression is not periodic, in agreement with the absence of periodicity of the vascular pattern in the root. The plants were grown for 5 weeks in long-day conditions Scale bars: 100 μm (A,C,E,G,I) or 25μm (B,D,F,H,J).
(TIF)

S11 Fig Quantification of the phenotype of *aux1lax1lax2lax3* grown in long day conditions. (A-C) Boxplots of VB number (A), vascular unit size (B), and total cell number across the provascular ring (C) for WT (n = 18) and *aux1lax1lax2lax3* (n = 15) mutant vascular rings in long day conditions. No significant statistical differences are found (all p-values obtained are larger than 0.04). (D) Frequency distribution of the number of undifferentiated cell layers in long day conditions for WT (n = 53 VBs), *aux1lax1lax2lax3* (n = 67 VBs) and *pin1pin2* (n = 40 VBs). *aux1lax1lax2lax3* mutant in long day shows increased number of undifferentiated cell layers, whereas the *pin1pin2* mutant is similar to WT. The phenotype of *aux1lax1lax2lax3* is milder than in short day conditions.
(TIF)

S12 Fig Efflux carriers are required for periodic patterning but do not change strongly the fastest growing mode that destabilizes the homogeneous state. (A) Snapshot of simulation results showing altered distribution of cytosolic (blue) and apoplastic (green) auxin along a ring of cells at time *t* = 17.5 as in Fig 1 but for reduced amount of efflux carriers (*E* = 10 μM s^-1^). (B) Phase diagram obtained from theoretical linear stability analysis on a ring of 60 cells on the parameter space of influx (*I*) and efflux (*E*) carriers levels. The solid line divides the space in two regions (Material and Methods): in the H region (white, below the solid line) the homogeneous state is linearly stable and no periodic pattern can be formed from small perturbations of it. In the P region (colored, above the solid line) the homogeneous state is linearly unstable and a periodic pattern can arise from it. According to this phase diagram, efflux but not influx carriers are essential to drive a pattern. The color scale shows the theoretical estimation of the inverse value of the number of cells between cytosolic auxin maxima (*κ*). The number of cells changes as the influx carriers *I* is increased and it is almost unmodified when the efflux carriers *E* change. Other parameter values are the same as in Fig 1.
(TIF)

S13 Fig Influx carriers reduce the differences and concentrations of auxin in the apoplast. Boxplots extracted from simulation results of Fig 1 that evaluate auxin concentration in the cytosol (top panels, blue boxplots) and in the apoplast (bottom panels, green boxplots) showing the amplitude of the pattern of auxin (A), the averaged auxin maxima (B) and minima (C) levels and the averaged auxin values along the vascular ring (D) as a function of the influx carriers *I*. The pattern amplitude is computed as the difference from the average auxin concentration at maximums (B) and the average auxin concentration at minimums (C) in each simulated ring of 60 cells and apoplast compartments. The values represented in the boxplots correspond to the averages performed within a ring. Dotted lines in panel (D) correspond to the theoretical auxin homogeneous steady states given by Eqs S9 and S35. Each boxplot depicts the results for 30 simulations with different initial auxin distributions (Material and Methods). Details of the depicted boxplot components can be found in Fig 1B. Crosses represent outliers. Simulations were done until time t = 17.5. Other parameter values are the same as in Fig 1.
(TIF)

S14 Fig Another example of the pervasive role of influx carriers on the concentration of auxin in the apoplast. Modeling results for a scenario with non auxin-induced carriers and low apoplastic diffusion. (A) Snapshots of simulation results showing periodic distribution of auxin inside and outside cells for higher (left, *I* = 100 μM s^-1^) and lower (right, *I* = 0.01 μM s^-1^) influx carriers levels along a ring of vascular tissue composed of 30 cells surrounded by the apoplast. Cytosolic (blue) and aploplastic (green) auxin concentrations at time t = 17.5 are shown. The red circular line represents the ring of cells in the tissue. Insets depict the same results projected into a 2D plane. Space is represented in arbitrary units [AU]. The number of auxin maxima is the same in both cases. (B-E, F-I) Simulation results showing the number of cytosolic auxin maxima over the total number of cells (B,F), the amplitude of the pattern of auxin (C,G), the averaged auxin maxima levels (D,H) and the averaged auxin values along the vascular ring (E,I) in the cytosol (top panels, blue boxplots) and in the apoplast (bottom panels, green boxplots) as a function of the influx carriers *I* (B-E) and the efflux carriers *E* (F-I). Each boxplot depicts the results for 30 simulations with different initial auxin distributions (Methods). Simulations in B-I were done for rings of 60 cells until time *t* = 17.5. Depicted boxplot components are the same as in Fig 1B. Crosses represent outliers. Other details of panels (B, F) are the same as in Fig 1B and Fig 5B and 5F. Dotted lines in panels (E,I) as in Fig 5E and 5I. Main parameter values: in all panels, *D* = 0.01 s^-1^ and *D*_*ca*_ = 15 s^-1^ with no auxin-induced synthesis of carriers (*θ*_*I*_ = *θ*_*P*_ = *0* μM), and *E* = 105 μM s^-1^ for A-D panels, while *I* = 100 μM s^-1^ for E-G panels. Other parameter values are the same as in Fig 1.
(TIF)

S15 Fig Changes of auxin concentration in the apoplast or the cytoplasm are not required for changes in the periodicity of the pattern. (A) Inverse value of the number of cells between auxin maxima. (B) Average levels of auxin in the cytosol (top, blue boxplot) and in the apoplast (bottom, green boxplot) as a function of the apoplastic diffusion coefficient (*D*). Results from numerical simulation of the model dynamics are shown by boxplots. Each boxplot depicts the results for 30 simulations with different initial auxin distributions (Materials and Methods) on a ring of 60 cells and 60 apoplastic compartments. Depicted boxplot components are the same as in Fig 1B. Thin solid lines in (A) are obtained from linear stability analysis on a ring of 60 (black) and 1200 (blue) cells, while vertical line indicates the critical apoplastic diffusion value below which the pattern cannot emerge, derived from linear stability analysis. Dotted lines in (B) panels as in Fig 5 (E and I). *I* = 0.1 μM s^-1^ and other parameter values are the same as in Fig 1.
(TIF)

S16 Fig Localization patterns of the auxin influx carrier proteins in the Arabidopsis shoot inflorescence stem in long day conditions. AUX1/LAX-VENUS reporters show localization in procambial, protoxylem and phloem cell files in the vascular bundles of Arabidopsis shoot stems. (A-C) *ProAUX1*:*AUX1*::*VENUS* fluorescence is present in procambial and protoxylem cell files. (D-F) *ProLAX1*:*LAX1*::*VENUS* fluorescence is present in procambial and protoxylem cells. (G-I) *ProLAX2*:*LAX2*::*VENUS* fluorescence is present in procambial and protoxylem cells. (J-L) *ProLAX3*:*LAX3*::*VENUS* fluorescence is present in the phloem cell files. Left panels are transmission channels of the corresponding confocal image in the adjacent middle panel. Blue autofluorescence highlights xylem cells and interfascicular fibers. Pink arrowheads indicate protoxylem cells within the VB. White arrows indicate undifferentiated procambial cells between phloem and xylem cells. Phloem cells are indicated by green arrowhead. All plants were grown for 5 weeks in long day conditions. VENUS fluorescence images were acquired in hand-made sections from the Z1 zone of the stem. Scale bars: 100 μm.
(TIF)

S1 Text Model formulation, linear stability analysis, analytical expressions for pattern formation and dependence of the periodicity of the pattern and the average concentration of auxin on the amount of influx carriers. (PDF)

S1 Table Table of parameters values ranges related to auxin transport. (PDF)

S1 Video Simulation results show that influx carriers modulate auxin pattern periodicity. Simulation results of Fig 1A (in a different axis scale) showing the emergence of the auxin pattern for higher (left, *I* = 100 μM s^-1^) and lower (right, *I* = 0.001 μM s^-1^) influx carriers levels along a ring of vascular tissue composed of 60 cells surrounded by the apoplast. Cytosolic (blue) and apoplastic (green) auxin concentrations are shown. The red circular line represents the ring of cells in the tissue. Parameter values as in Fig 1A.
(AVI)

S1 Code Mathematica code for simulating auxin transport in a provascular tissue ring. Mathematica notebook that performs the numerical integration of the model Eqs S7 and S8 along a line of 60 cells and apoplastic compartments.
(NB) Click here for additional data file.
