# Correction: Auxin Influx Carriers Control Vascular Patterning and Xylem Differentiation in *Arabidopsis thaliana*

**DOI:** 10.1371/journal.pgen.1005296

**Published:** 2015-06-17

**Authors:** The PLOS Genetics Staff

## Notice of Republication

This article was republished on May 15, 2015, to correct Supporting Information files: S1, S5, S6, S8, S9, S10, S11 and S16 Figs, which were incorrectly published with a pink background. The publisher apologizes for the errors. Please download these files again to view the correct versions. The originally published, uncorrected files and the republished, corrected files are provided here for reference.

## Supporting Information

S1 File Originally published, uncorrected supporting information files. (ZIP)

S2 File Republished corrected supporting information files (ZIP)
