# Supplementary figures and images for: Auxin Influx Carriers Control Vascular Patterning and Xylem Differentiation in Arabidopsis thaliana
Source: PLoS Genet. 2015 Apr 29;11(4):e1005183. doi: 10.1371/journal.pgen.1005183 (PMC4414528; doi:10.1371/journal.pgen.1005183)

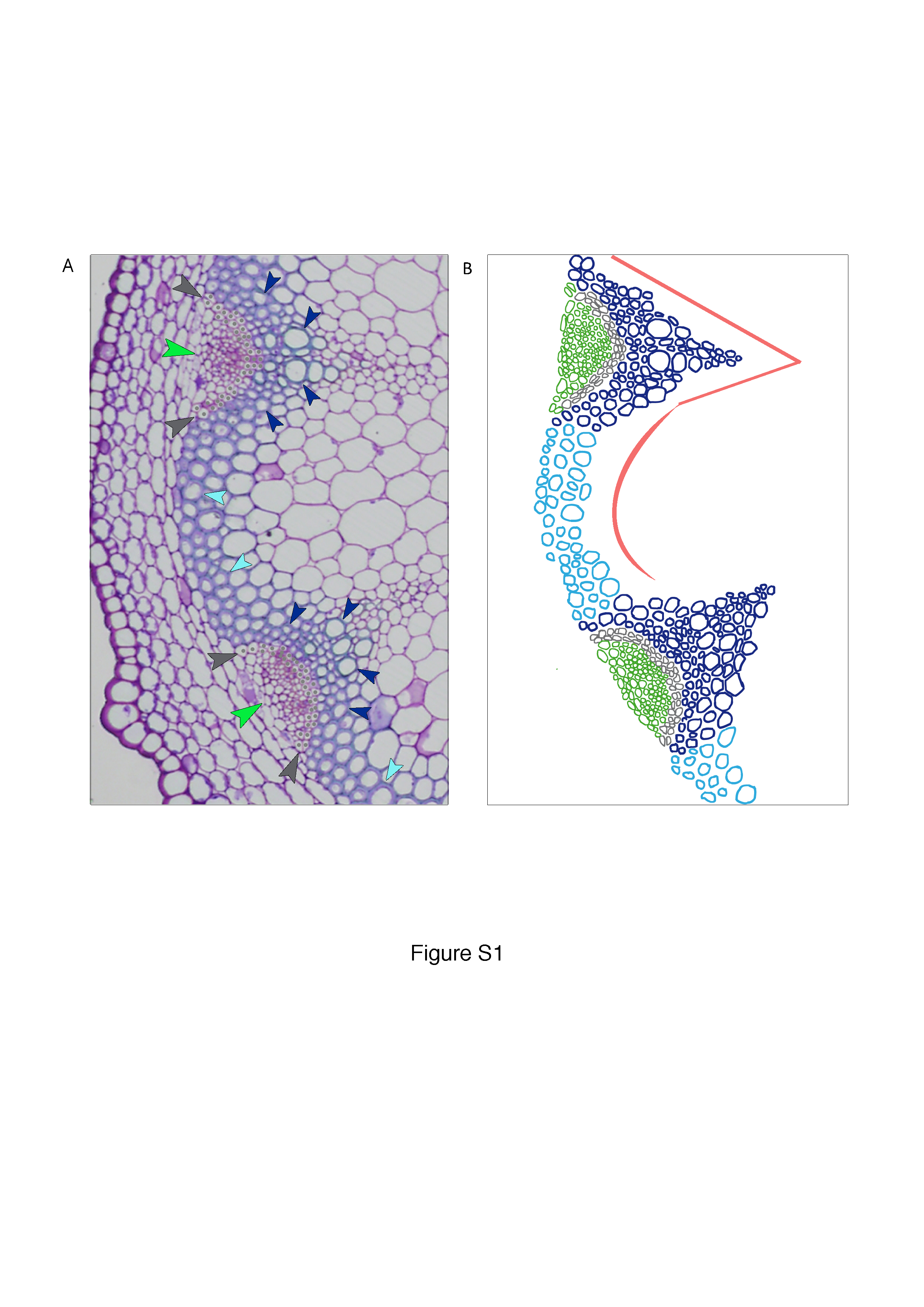

Supplement: S1 Fig — (A) Magnification of a shoot basal cross section for a 5-week-old Arabidopsis WT plant. Grey arrowheads indicate the beginning and the end of the procambial cells layers within a VB. Procambial cells are depicted in grey. Green arrowheads indicate phloem cells. Dark blue arrowheads show the xylem cells in the VB. Light blue arrowheads show the IF cells (B) Cartoon of the WT plant represented in (A) where procambial cells are depicted in grey, phloem cells are depicted in green, VB xylem cells in dark blue and IF cells in light blue. Red line indicates the length of one vascular unit formed by one VB and their immediate IF cells. (TIF) [file pgen.1005183.s001.tif]

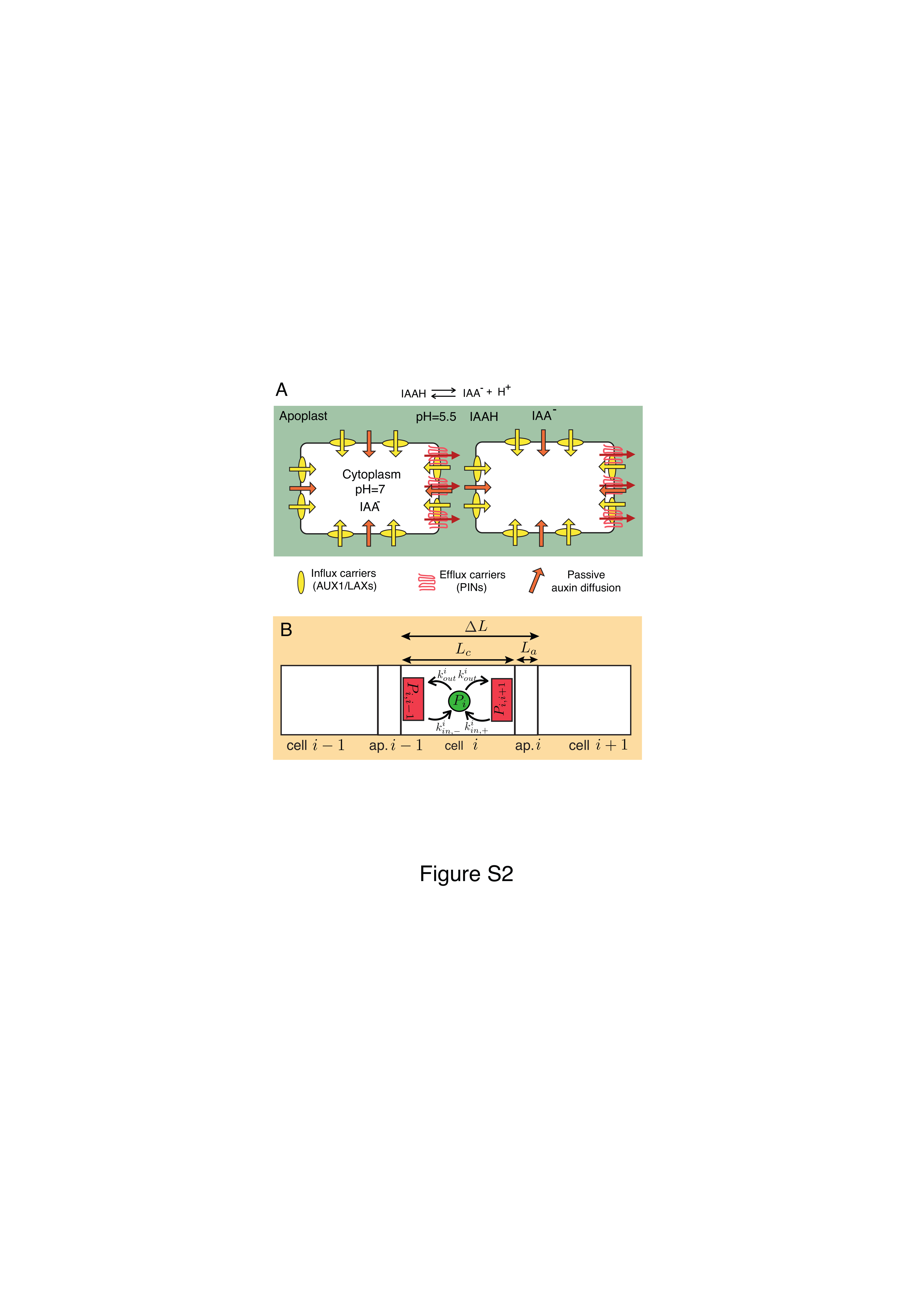

Supplement: S2 Fig — (A) Chemiosmotic model for auxin transport. Auxin can be in its protonated or anionic form, (IAAH and IAA-, respectively). Red arrows represent the auxin flux driven by PIN efflux carriers, which are asymmetrically localized on the membrane; yellow arrows represent the auxin flux driven by AUX1/LAX influx carriers. Orange arrows denote passive entrance of auxin into the cell. Being auxin a weak acid, once it enters the cells, where the pH is less acidic than in the apoplast, it gets deprotonated and, consequently, trapped inside. Therefore, auxin can only exit via the action of efflux carriers, such as PINs, which have a polarized localization on the membrane, conferring directionality to auxin transport. (B) Modeling scheme illustrating the cellular ("cell") and apoplastic ("ap.") spaces, and the cycling of the efflux carriers within cells. The labeling (i) of cells and apoplasts used in the mathematical equations is also indicated. We model the apoplast as a compartment between cells, and we set effective auxin apoplastic diffusion between the two apoplasts that are adjacent to a cell (e.g. the apoplasts adjacent to cell i are apoplasts i-1 and i; extracellular auxin can diffuse then from apoplast i to apoplast i-1 and vice versa). Efflux carriers are asymmetrically distributed in the cell membrane since their cycling rates to the different membrane segments in a cell are also asymmetric. Influx carriers are symmetrically distributed throughout the cell membrane. (TIF) [file pgen.1005183.s002.tif]

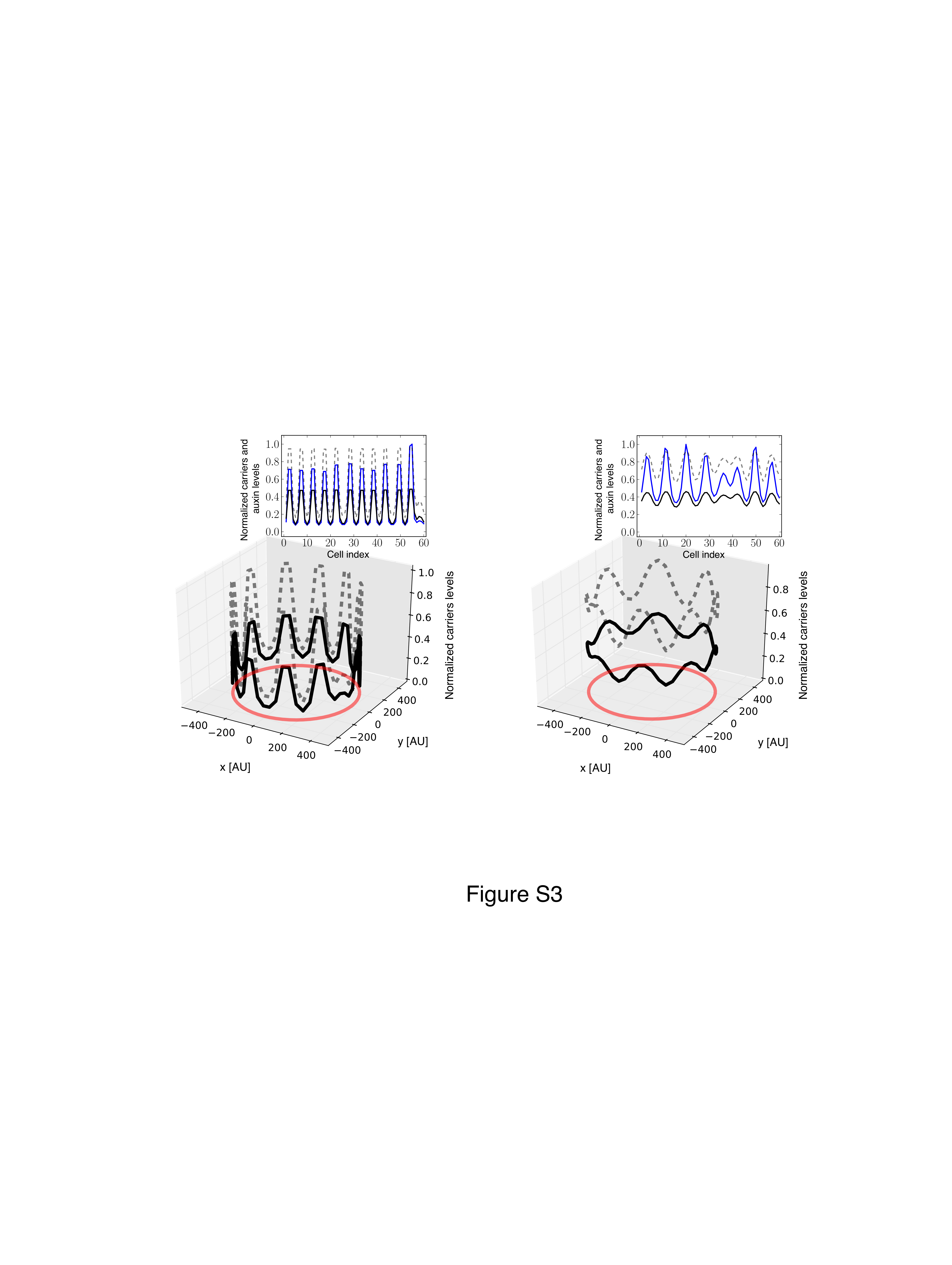

Supplement: S3 Fig — For the parameter values of Fig 1A with higher (left, I = 100 μM s-1) and lower (right, I = 0.001 μM s-1) influx carriers levels, we show the distribution of influx (solid black line) and efflux (dashed gray line) carriers together with cytosolic auxin (blue line). The levels of carriers is normalized to 1/2 for the influx and 1 for the efflux, and corresponds to IT(Ai)=12AiθI+Ai and PT(Ai)=AiθP+Ai respectively (see S1 Text). Cytosolic auxin has been normalized to 1. (TIF) [file pgen.1005183.s003.tif]

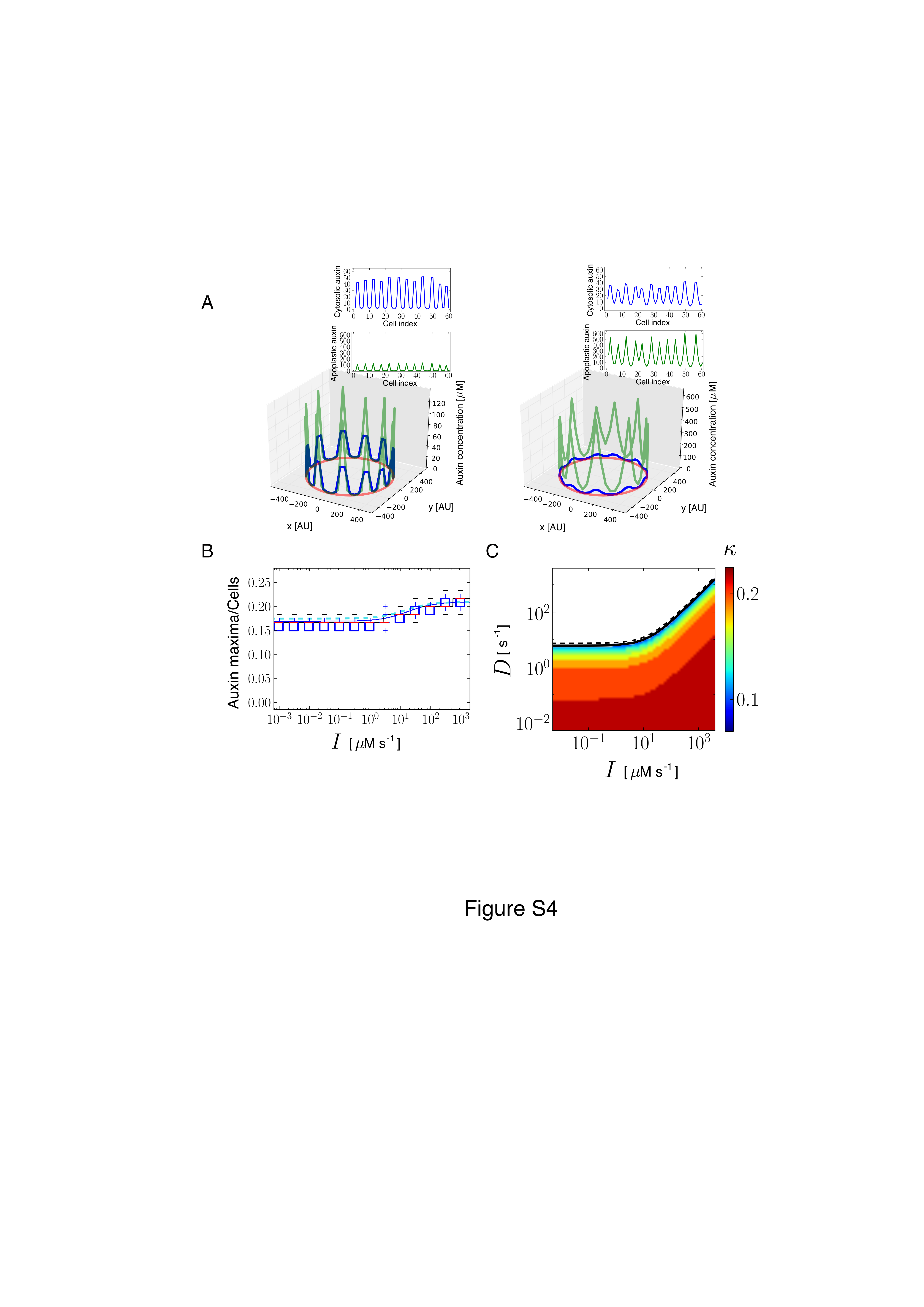

Supplement: S4 Fig — The results correspond to a scenario with constant total amount of carriers per cell (no auxin-induced synthesis of carriers, θ I = θ P = 0 μM). Panels A-C as in Fig 1. (A) Snapshots of simulation results showing periodic distribution of auxin inside and outside cells for higher (left, I = 100 μM s-1) and lower (right, I = 0.001 μM s-1) influx carriers levels along a ring of vascular tissue composed of 60 cells surrounded by the apoplast. Cytosolic (blue) and apoplastic (green) auxin concentrations at time t = 17.5 are shown. The red circular line represents the ring of cells in the tissue. Insets depict the same results projected into a 2D plane. Space is represented in arbitrary units [AU]. (B) Simulation (boxplot) and theoretical estimation (κ, depicted by solid lines) results of the inverse value of the number of cells between cytosolic auxin maxima at different influx levels (I) for D = 2 s-1. Each boxplot depicts the results for 30 simulations with different initial auxin distributions (Materials and Methods). Simulations were done for rings of 60 cells. Depicted boxplot components are the same as in Fig 1B. Crosses represent outliers. The theoretical estimation is performed through linear stability analysis for a ring of 60 and 1200 cells (black and blue solid lines, respectively). The dashed light blue line is obtained from the analytical expression in S1 Text (Eqs S32-S33). (C) Phase diagram obtained from theoretical linear stability analysis on a ring of 60 cells in the parameter space of influx parameter (I) and apoplastic diffusion parameter (D). The solid line divides the space in two regions, as in Fig 1C. Above the solid line the homogeneous state is linearly stable and no periodic pattern can be formed from small perturbations of it. Below the solid line, the homogeneous state is linearly unstable and a periodic pattern can arise from it. The dashed black line is obtained from the analytical expression in S1 Text (Eq S34). The color scale shows the t [file pgen.1005183.s004.tif]

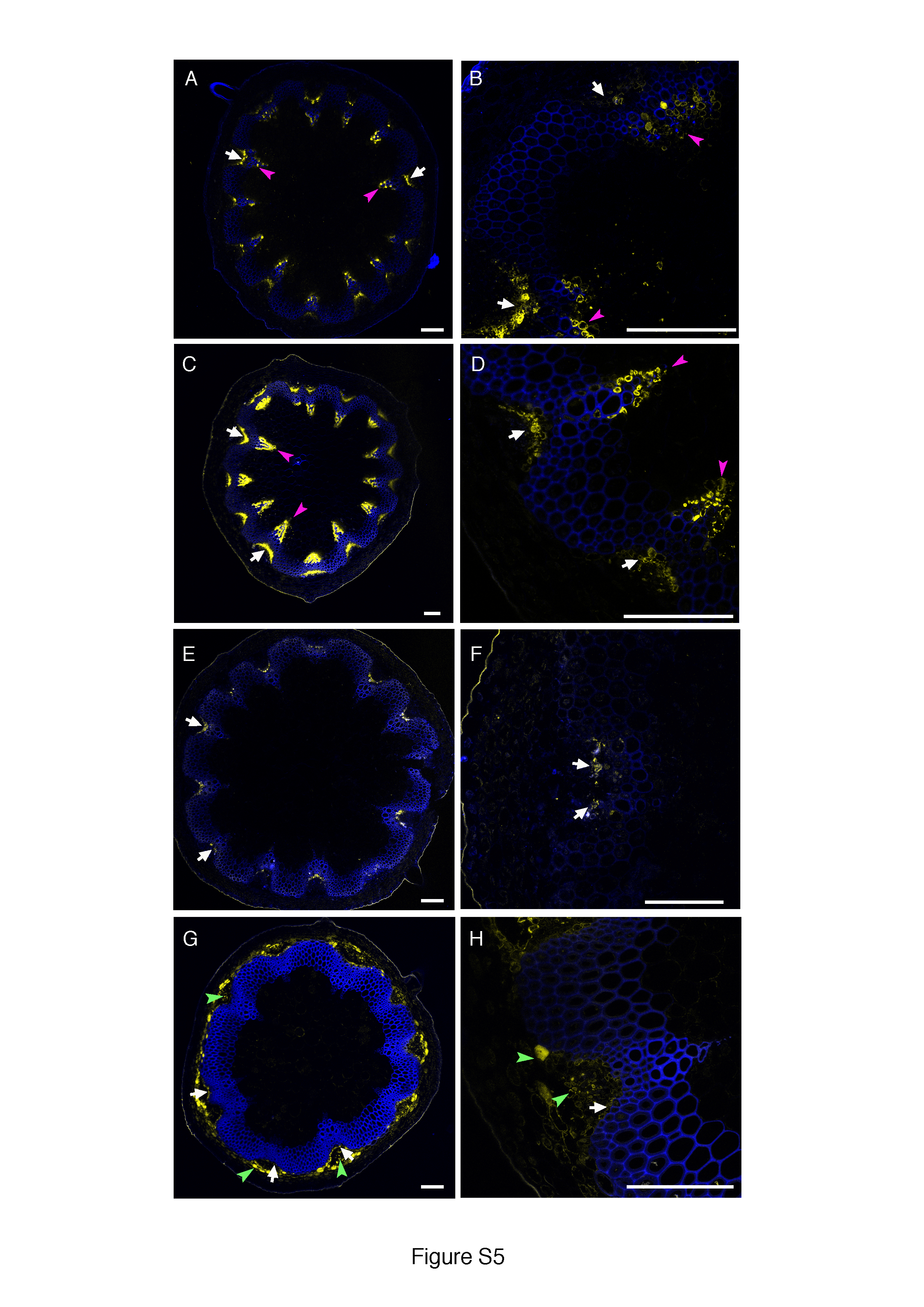

Supplement: S5 Fig — AUX1/LAX-VENUS reporters show localization in procambial, protoxylem and phloem cell files in the vascular bundles of Arabidopsis shoot stems. (A,B) ProAUX1:AUX1::VENUS fluorescence is present in procambial and protoxylem cell files. (C,D) ProLAX1:LAX1::VENUS fluorescence is present in procambial and protoxylem cells. (E,F) ProLAX2:LAX2::VENUS fluorescence is present in procambial cells. (G,H) ProLAX3:LAX3::VENUS fluorescence is present in procambial and in the phloem cell files. Blue autofluorescence highlights xylem cells and interfasciular fibers. Pink arrowheads indicate protoxylem cells within the VB. White arrows indicate undifferentiated procambial cells between phloem and xylem cells. Phloem cells are indicated by green arrowheads. All plants were grown for 7–11 weeks in short day conditions. Images were collected from cross sections at the basal part of the shoot inflorescence stem. Scale bars: 100 μm. (TIF) [file pgen.1005183.s005.tif]

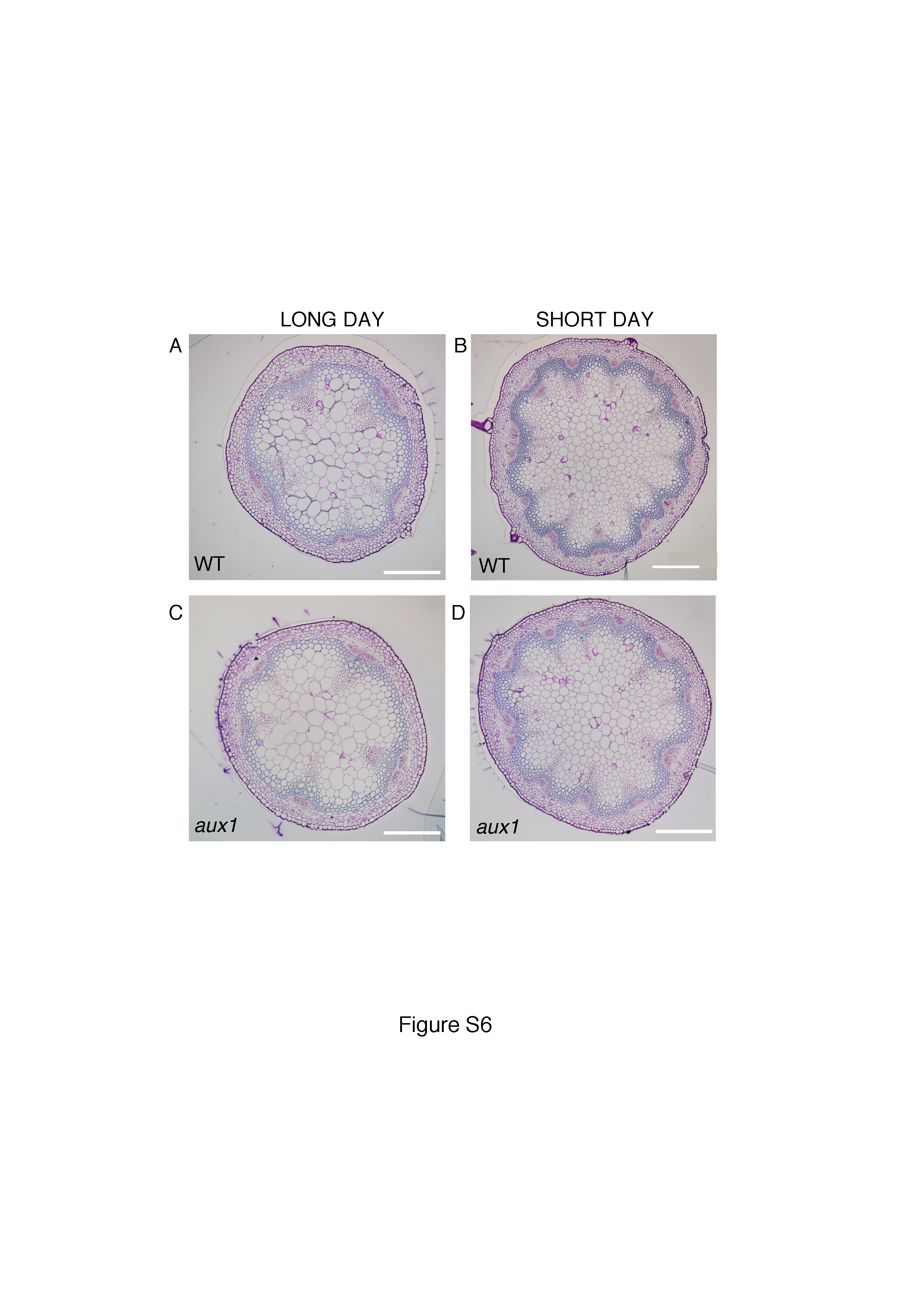

Supplement: S6 Fig — (A,C) Shoot inflorescence stems for WT 5-weeks-old plant (A) and aux1 mutant 5-week-old plant (C), grown in long day conditions (B,D) Shoot inflorescence stems for WT 14-weeks-old plant (B) and aux1 mutant 14-week-old plant (D), grown in short day conditions. Scale bars: 250 μm. p-values of the VB numbers of WT versus aux1 mutants are 0.56 in short day conditions (n = 12 for WT and n = 12 for aux1 mutants) and 1.0 in long day conditions (n = 6 for WT and n = 6 for aux1 mutants), what shows no statistical difference. (TIF) [file pgen.1005183.s006.tif]

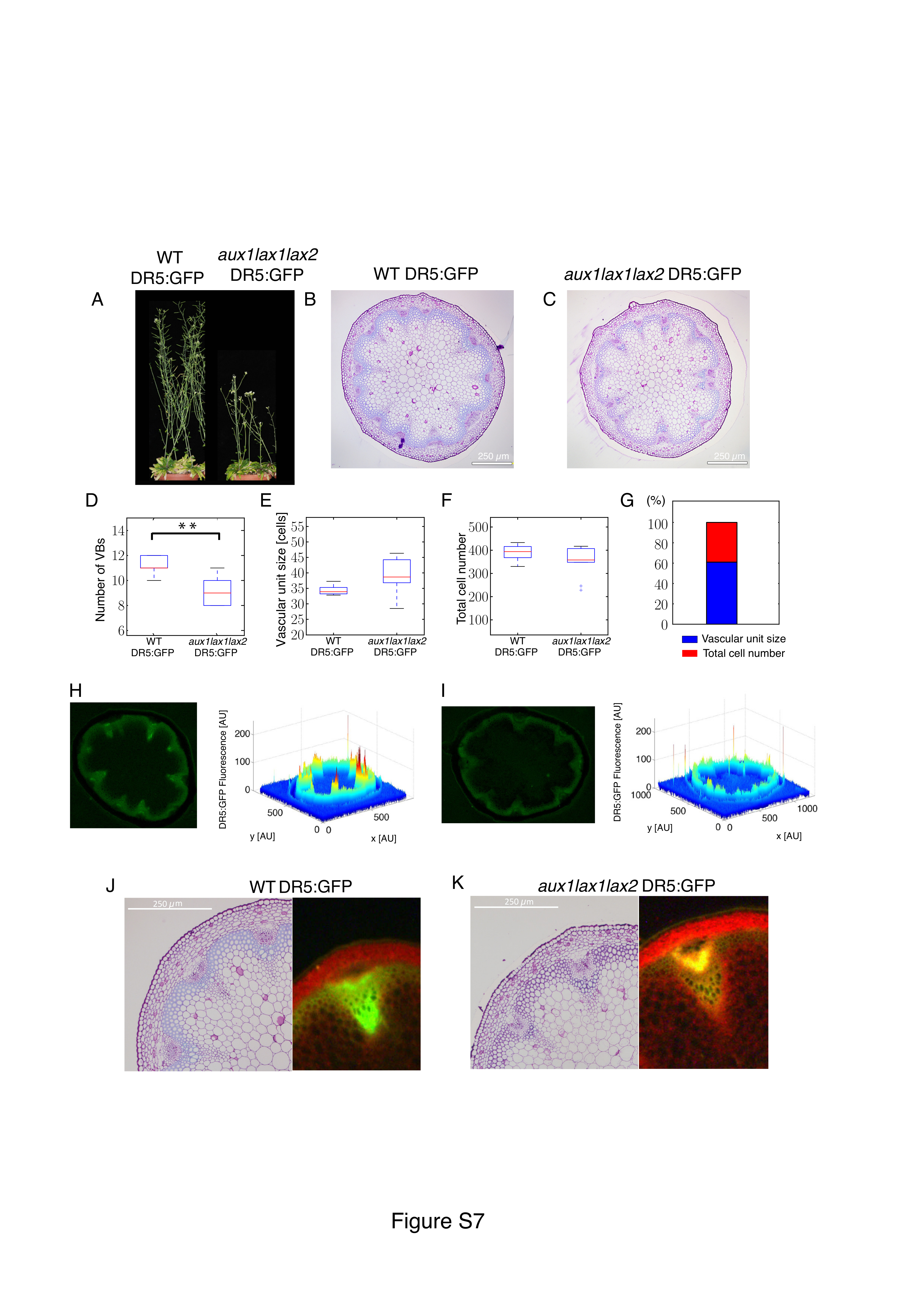

Supplement: S7 Fig — (A) WT DR5:GFP 14-weeks-old plant (left) and aux1lax1lax2 DR5:GFP triple mutant 14-week-old plant (right), grown in short day conditions. (B) Basal shoot cross section of DR5:GFP in Col-0 WT background. (C) Basal shoot cross section of DR5:GFP in aux1lax1lax2 triple mutant background. (D-F) Boxplots of VB number (D), vascular unit (cells/VB) (E) and total cell number (F) for WT DR5:GFP and aux1lax1lax2 DR5:GFP mutant. For the total cell number quantification along the shoot stem section, the ring of cells formed by the interfascicular fiber cells and the procambial cells within the vascular bundle were taken into account. (G) Percentage of contribution of VB spacing and total cell number on the change in VB number in the aux1lax1lax2 DR5:GFP mutant. The VB spacing (p-value = 0.057) and the total cell number (p-value = 0.124) show the same trends as in the quadruple, but they are not statistically significantly altered. This suggests that despite neither of these two trends is statistically significant on its own, together they drive the significant change in VB number (p-value = 0.001). Moreover, the contribution of each trend to the reduction in VB number in the triple mutants is as marked as in the quadruple mutants (VB spacing can explain 61% of the change in VB number). (H, I) Cross section GFP fluorescence of (H) WT DR5:GFP and (I) aux1lax1lax2 DR5:GFP plants. (Right panels) 3D density plots showing the GFP intensities in GFP fluorescence of (H) WT DR5:GFP and (I) aux1lax1lax2 DR5:GFP plants. For facilitating the comparison between right panels in H and I, both 3D plots have been colored according the fluorescence levels in arbitrary units, following the same color scale. (J,K) Left panels show VB magnification of a shoot basal cross section for WT DR5:GFP (J) and aux1lax1lax2 DR5:GFP mutant (K). (J, K) Right panels show VB magnification GFP fluorescence of (J) WT DR5:GFP and (K) aux1lax1lax2 DR5:GFP plants. All plants were grown under short day conditions. Pan [file pgen.1005183.s007.tif]

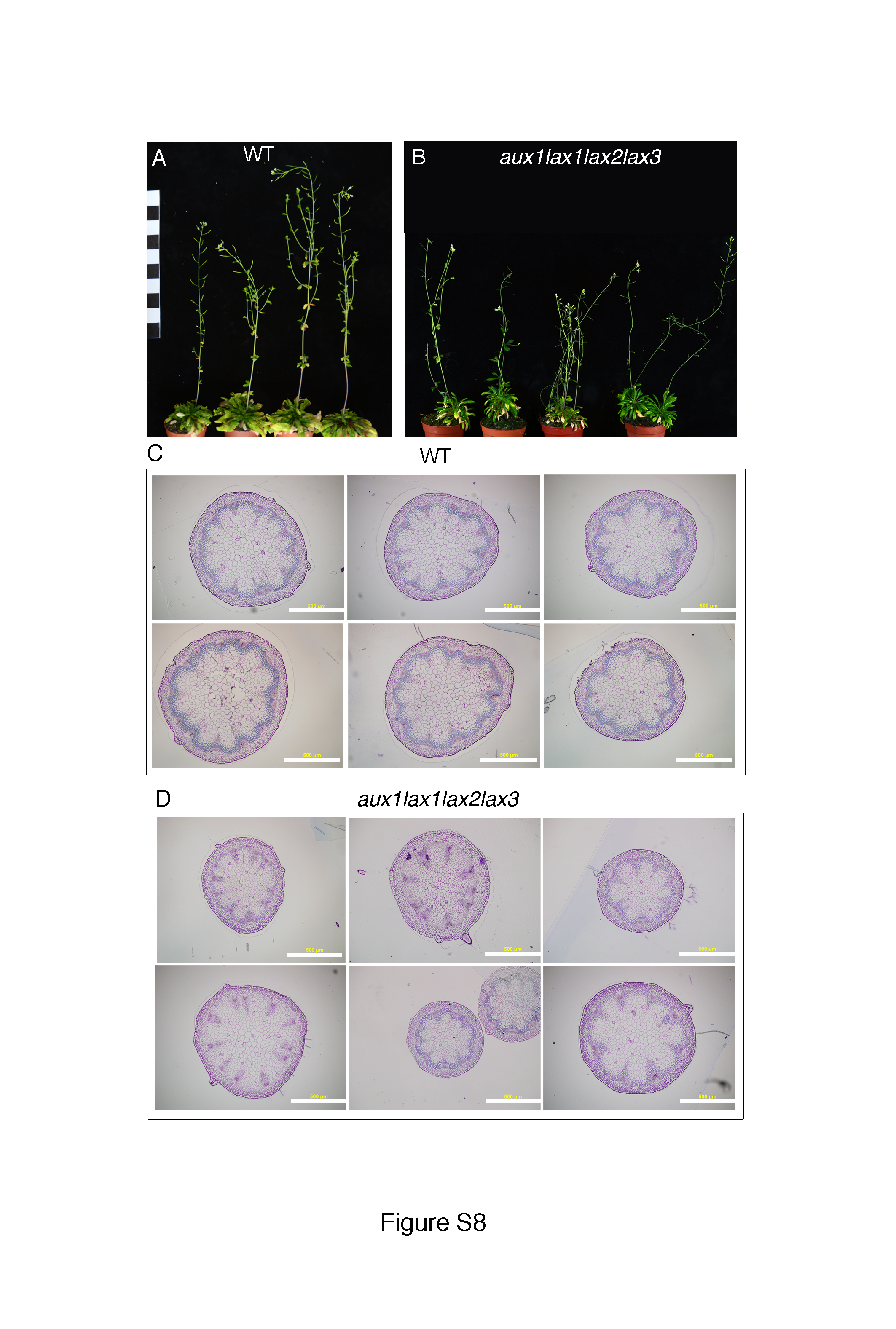

Supplement: S8 Fig — (A) WT 14-weeks-old plants. (B) aux1lax1lax2lax3 mutant 14-weeks-old plants. Note that aux1lax1lax2lax3 quadruple mutants adult plants display shorter and more diverse stems than WT plants. (C) Basal shoot cross section of six independent WT plants. (D) Basal shoot cross section of six independent aux1lax1lax2lax3 quadruple mutant plants. All the plants were grown in short day conditions. (TIF) [file pgen.1005183.s008.tif]

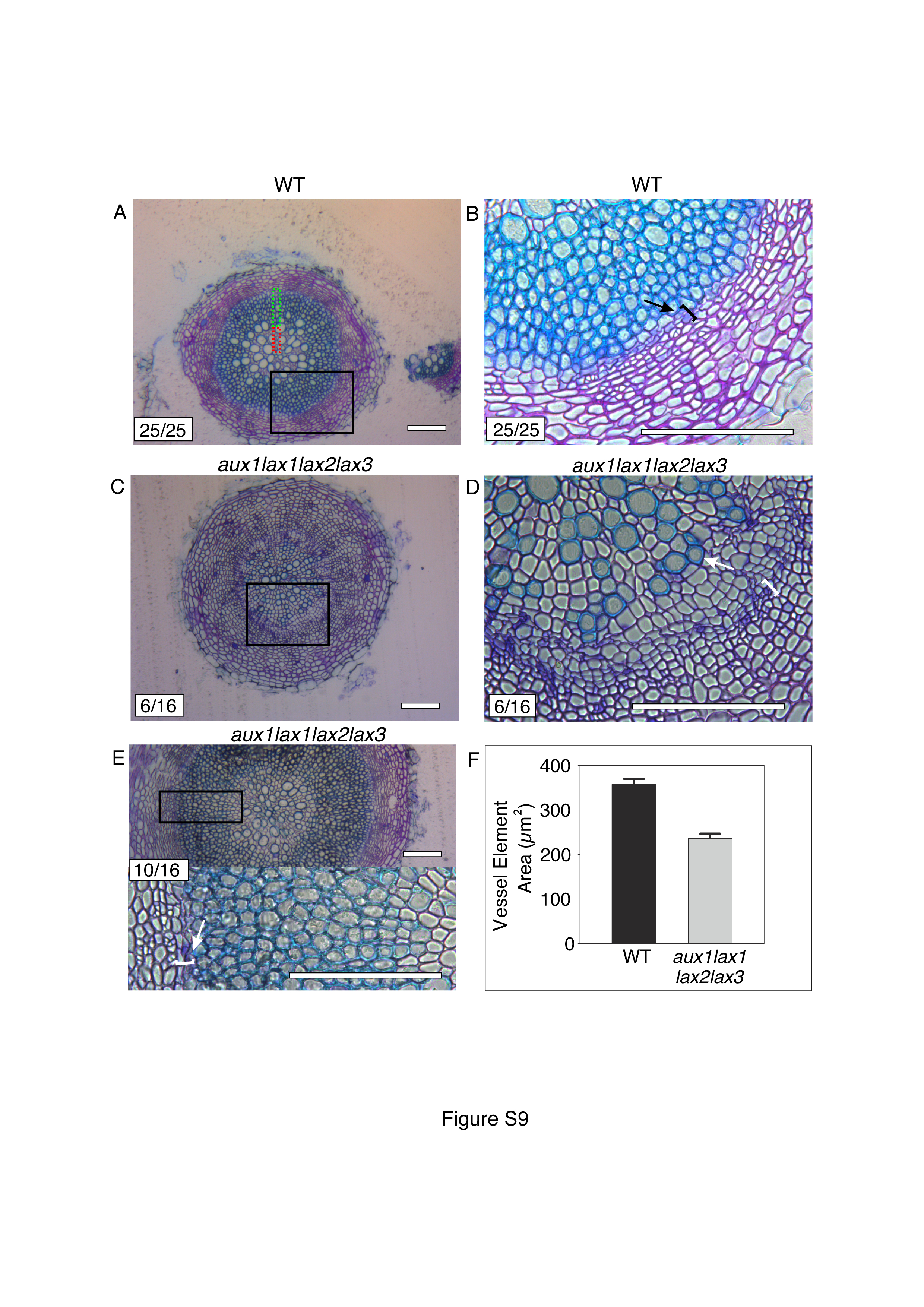

Supplement: S9 Fig — (A-E) Root cross sections for WT (A-B) and aux1lax1lax2lax3 quadruple mutant (C-E). The quadruple mutant showed either defects in vessel differentiation (C, D) or a phenotype that resembled the WT (E). The frequencies of the observed phenotypes are indicated in the images (number of roots showing the phenotype/number of roots analyzed). Arrows: most recent vessel element. Square brackets: cambial region. Red rectangle: primary phase of secondary xylem development. Green rectangle: secondary phase of secondary xylem development. (F) Vessel element area in WT (left column; n = 559 from 25 plants) and aux1lax1lax2lax3 quadruple mutant (right column; n = 640 from 18 plants) measured in the primary phase of secondary xylem development. p-value ≤ 0.001, Mann-Whitney test. Data represent the average ± 95% CI. Scale bars: 100 μm. (TIF) [file pgen.1005183.s009.tif]

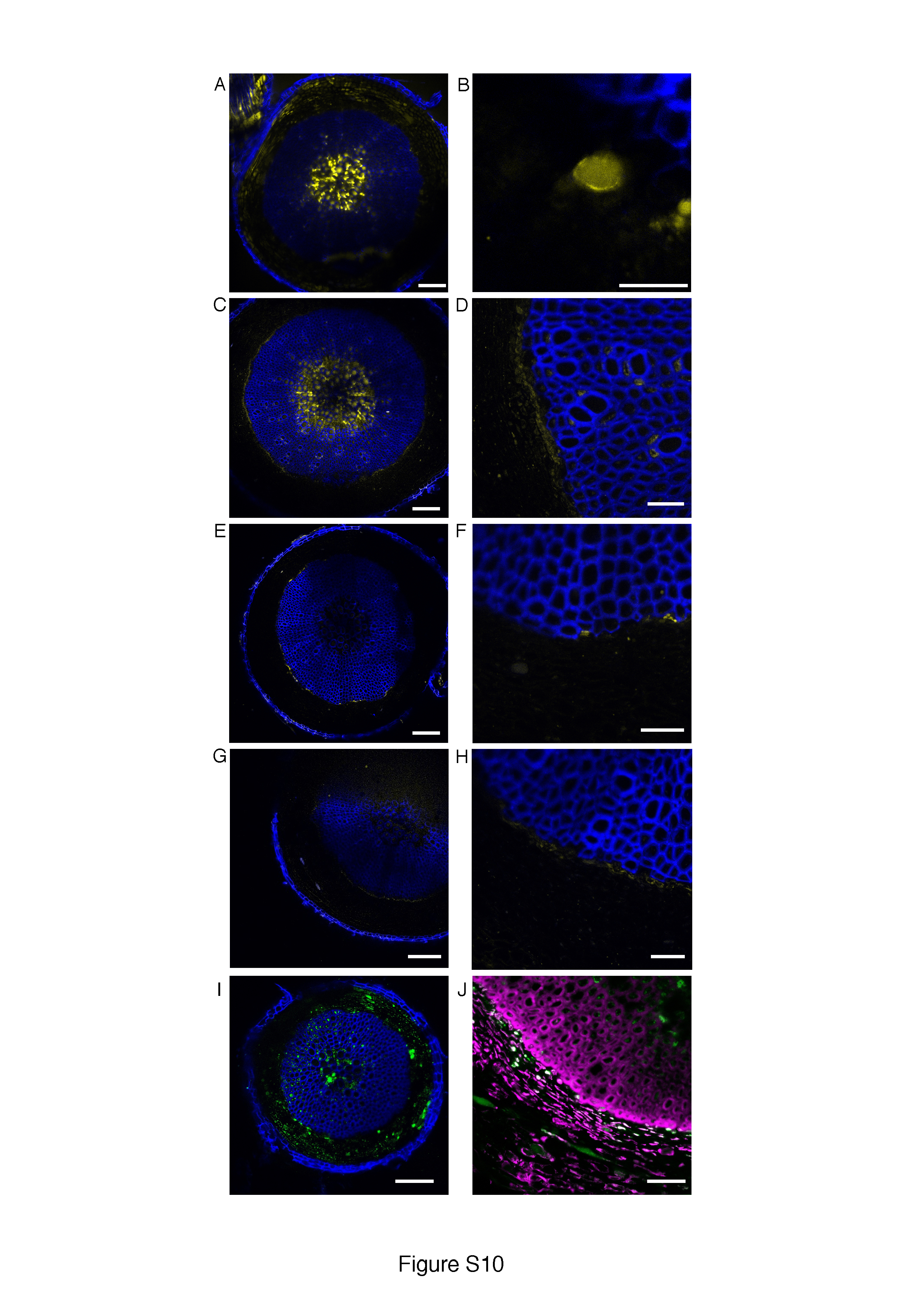

Supplement: S10 Fig — The AUX1/LAX reporter lines proAUX1:AUX1-VENUS (A,B), proLAX1:LAX1-VENUS (C,D), proLAX2:LAX2-VENUS (E,F), proLAX3:LAX3-VENUS (G,H) are localized to cambium and differentiating xylem in roots. (I,J) WT DR5::GFP plants show expression in differentiating xylem of the root cambium. The expression is not periodic, in agreement with the absence of periodicity of the vascular pattern in the root. The plants were grown for 5 weeks in long-day conditions Scale bars: 100 μm (A,C,E,G,I) or 25μm (B,D,F,H,J). (TIF) [file pgen.1005183.s010.tif]

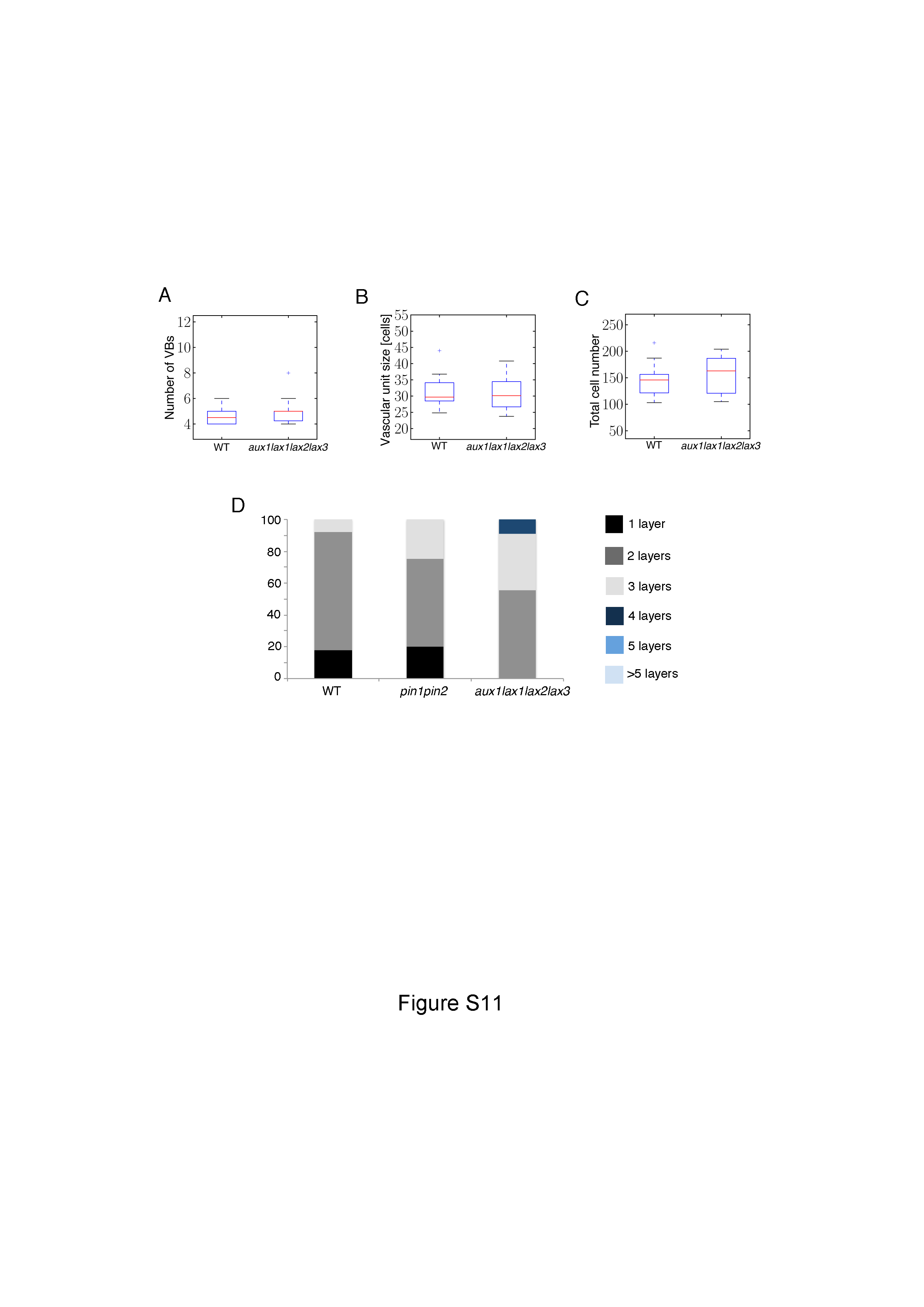

Supplement: S11 Fig — (A-C) Boxplots of VB number (A), vascular unit size (B), and total cell number across the provascular ring (C) for WT (n = 18) and aux1lax1lax2lax3 (n = 15) mutant vascular rings in long day conditions. No significant statistical differences are found (all p-values obtained are larger than 0.04). (D) Frequency distribution of the number of undifferentiated cell layers in long day conditions for WT (n = 53 VBs), aux1lax1lax2lax3 (n = 67 VBs) and pin1pin2 (n = 40 VBs). aux1lax1lax2lax3 mutant in long day shows increased number of undifferentiated cell layers, whereas the pin1pin2 mutant is similar to WT. The phenotype of aux1lax1lax2lax3 is milder than in short day conditions. (TIF) [file pgen.1005183.s011.tif]

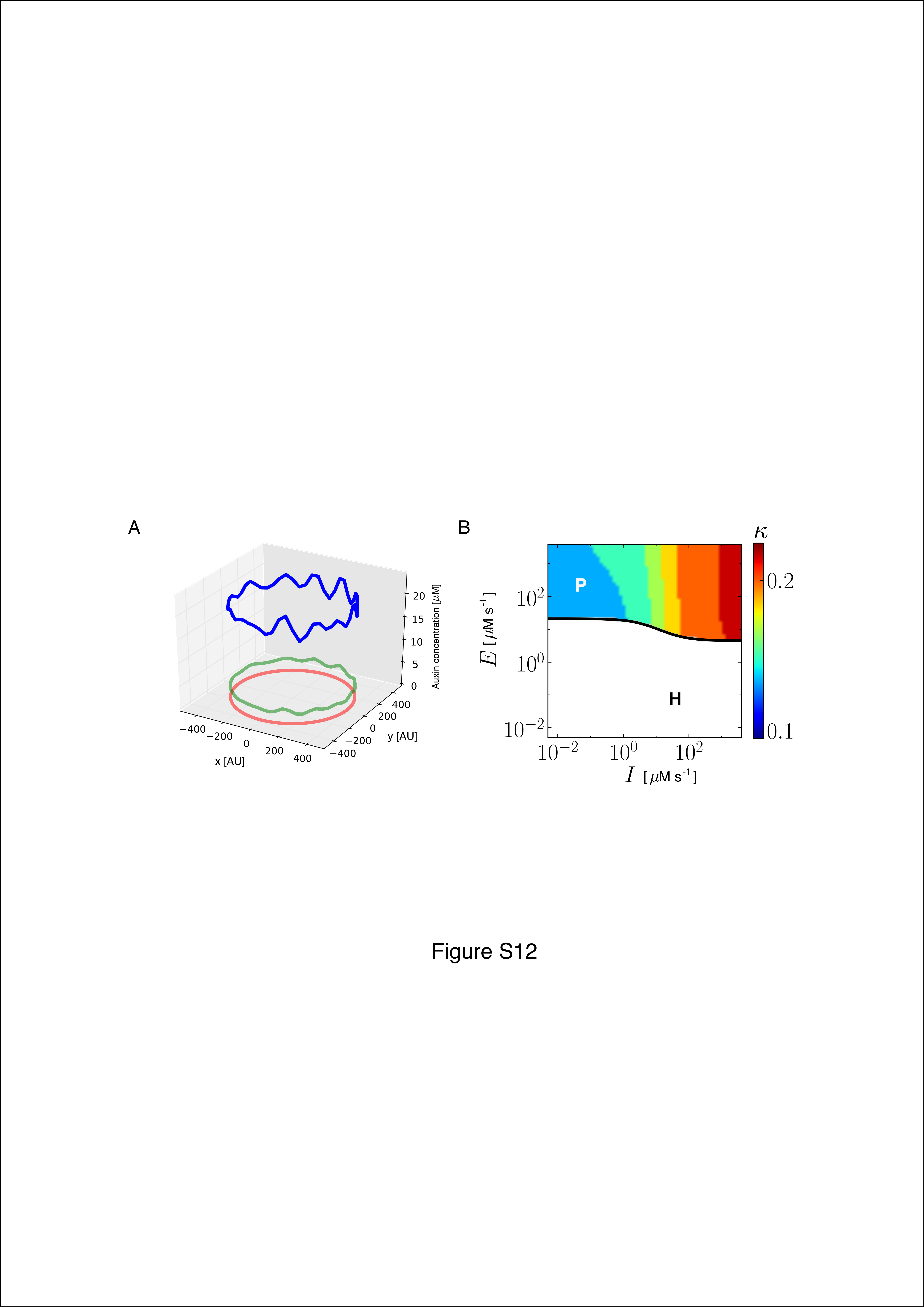

Supplement: S12 Fig — (A) Snapshot of simulation results showing altered distribution of cytosolic (blue) and apoplastic (green) auxin along a ring of cells at time t = 17.5 as in Fig 1 but for reduced amount of efflux carriers (E = 10 μM s-1). (B) Phase diagram obtained from theoretical linear stability analysis on a ring of 60 cells on the parameter space of influx (I) and efflux (E) carriers levels. The solid line divides the space in two regions (Material and Methods): in the H region (white, below the solid line) the homogeneous state is linearly stable and no periodic pattern can be formed from small perturbations of it. In the P region (colored, above the solid line) the homogeneous state is linearly unstable and a periodic pattern can arise from it. According to this phase diagram, efflux but not influx carriers are essential to drive a pattern. The color scale shows the theoretical estimation of the inverse value of the number of cells between cytosolic auxin maxima (κ). The number of cells changes as the influx carriers I is increased and it is almost unmodified when the efflux carriers E change. Other parameter values are the same as in Fig 1. (TIF) [file pgen.1005183.s012.tif]

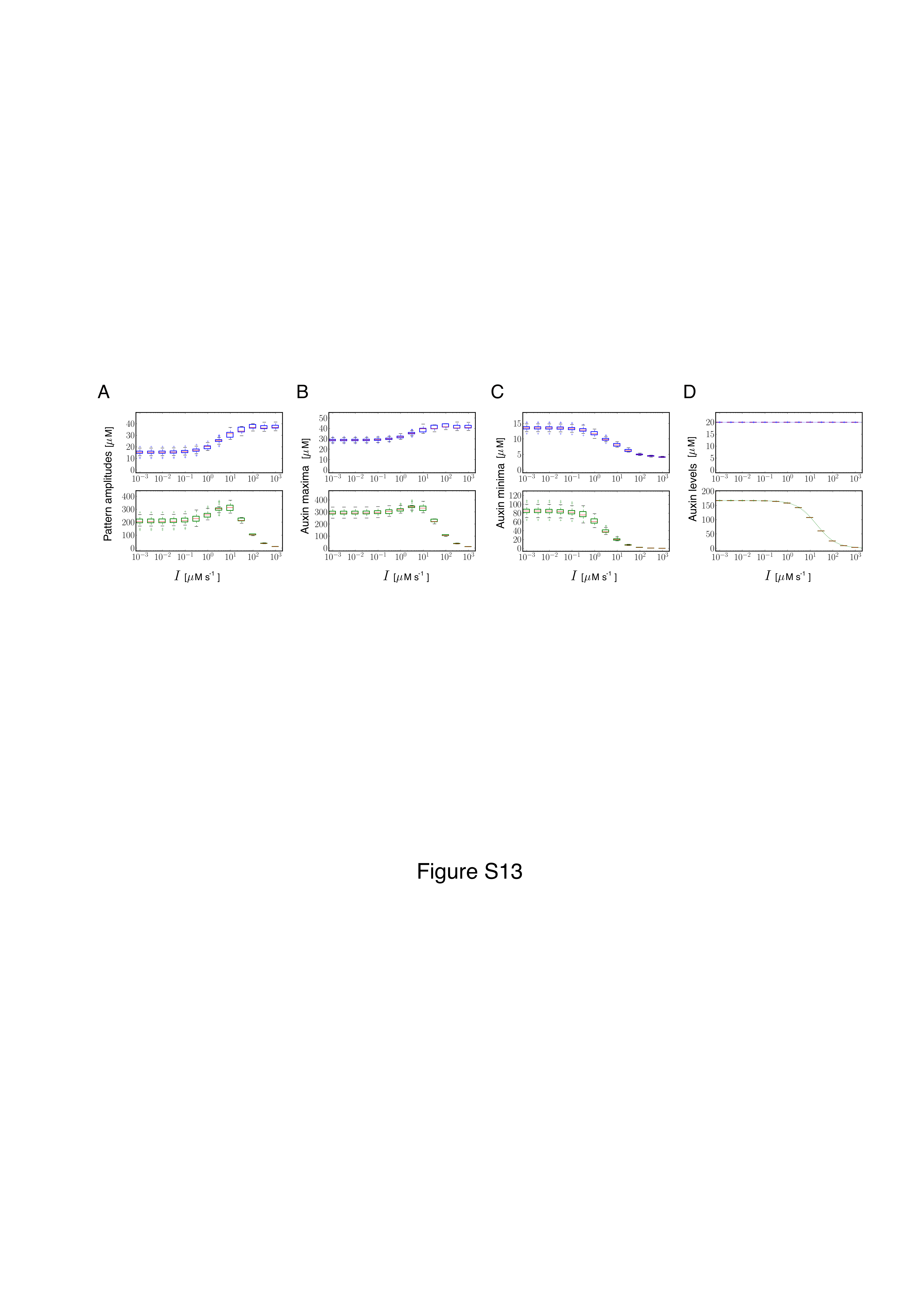

Supplement: S13 Fig — Boxplots extracted from simulation results of Fig 1 that evaluate auxin concentration in the cytosol (top panels, blue boxplots) and in the apoplast (bottom panels, green boxplots) showing the amplitude of the pattern of auxin (A), the averaged auxin maxima (B) and minima (C) levels and the averaged auxin values along the vascular ring (D) as a function of the influx carriers I. The pattern amplitude is computed as the difference from the average auxin concentration at maximums (B) and the average auxin concentration at minimums (C) in each simulated ring of 60 cells and apoplast compartments. The values represented in the boxplots correspond to the averages performed within a ring. Dotted lines in panel (D) correspond to the theoretical auxin homogeneous steady states given by Eqs S9 and S35. Each boxplot depicts the results for 30 simulations with different initial auxin distributions (Material and Methods). Details of the depicted boxplot components can be found in Fig 1B. Crosses represent outliers. Simulations were done until time t = 17.5. Other parameter values are the same as in Fig 1. (TIF) [file pgen.1005183.s013.tif]

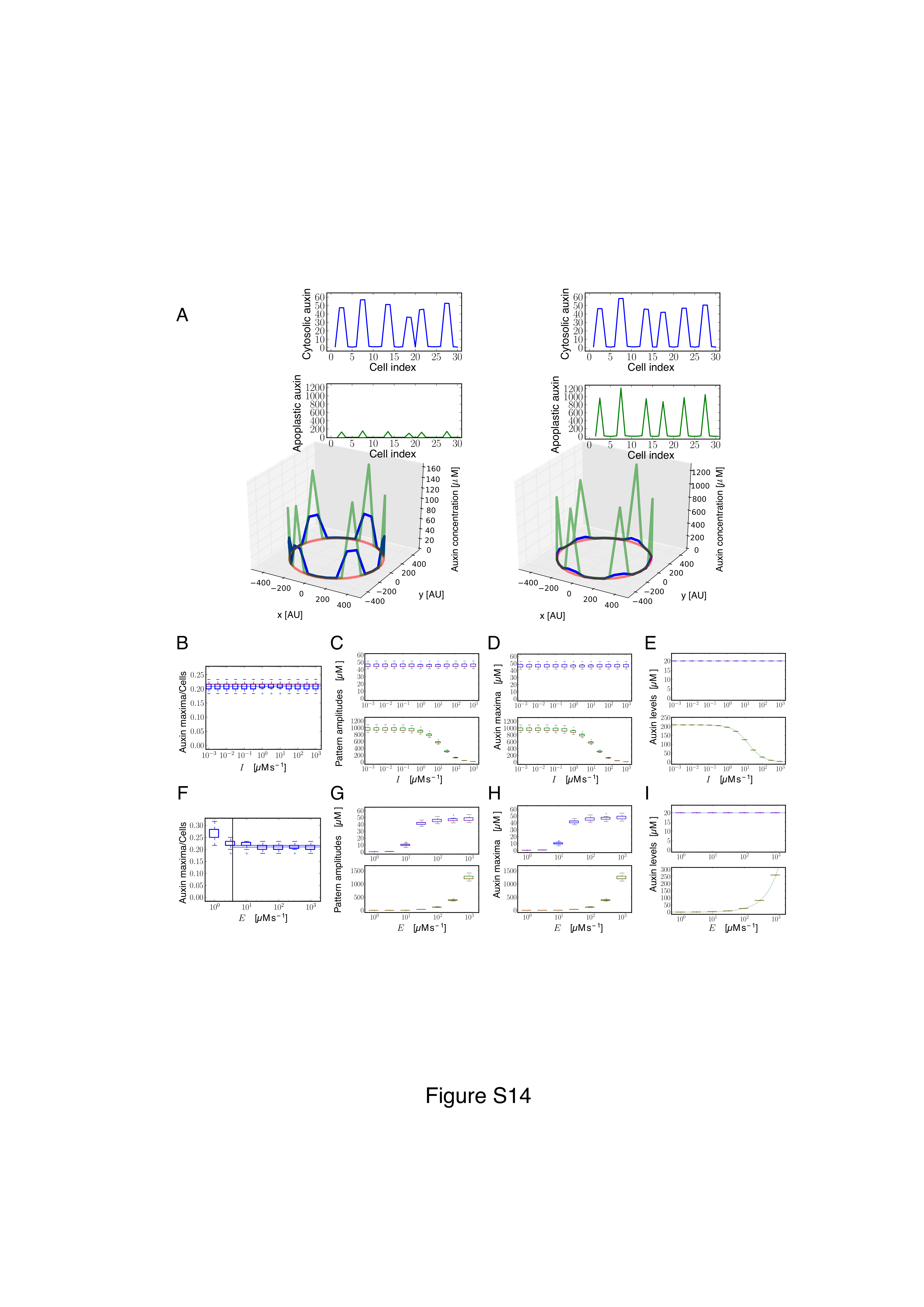

Supplement: S14 Fig — Modeling results for a scenario with non auxin-induced carriers and low apoplastic diffusion. (A) Snapshots of simulation results showing periodic distribution of auxin inside and outside cells for higher (left, I = 100 μM s-1) and lower (right, I = 0.01 μM s-1) influx carriers levels along a ring of vascular tissue composed of 30 cells surrounded by the apoplast. Cytosolic (blue) and aploplastic (green) auxin concentrations at time t = 17.5 are shown. The red circular line represents the ring of cells in the tissue. Insets depict the same results projected into a 2D plane. Space is represented in arbitrary units [AU]. The number of auxin maxima is the same in both cases. (B-E, F-I) Simulation results showing the number of cytosolic auxin maxima over the total number of cells (B,F), the amplitude of the pattern of auxin (C,G), the averaged auxin maxima levels (D,H) and the averaged auxin values along the vascular ring (E,I) in the cytosol (top panels, blue boxplots) and in the apoplast (bottom panels, green boxplots) as a function of the influx carriers I (B-E) and the efflux carriers E (F-I). Each boxplot depicts the results for 30 simulations with different initial auxin distributions (Methods). Simulations in B-I were done for rings of 60 cells until time t = 17.5. Depicted boxplot components are the same as in Fig 1B. Crosses represent outliers. Other details of panels (B, F) are the same as in Fig 1B and Fig 5B and 5F. Dotted lines in panels (E,I) as in Fig 5E and 5I. Main parameter values: in all panels, D = 0.01 s-1 and D ca = 15 s-1 with no auxin-induced synthesis of carriers (θ I = θ P = 0 μM), and E = 105 μM s-1 for A-D panels, while I = 100 μM s-1 for E-G panels. Other parameter values are the same as in Fig 1. (TIF) [file pgen.1005183.s014.tif]

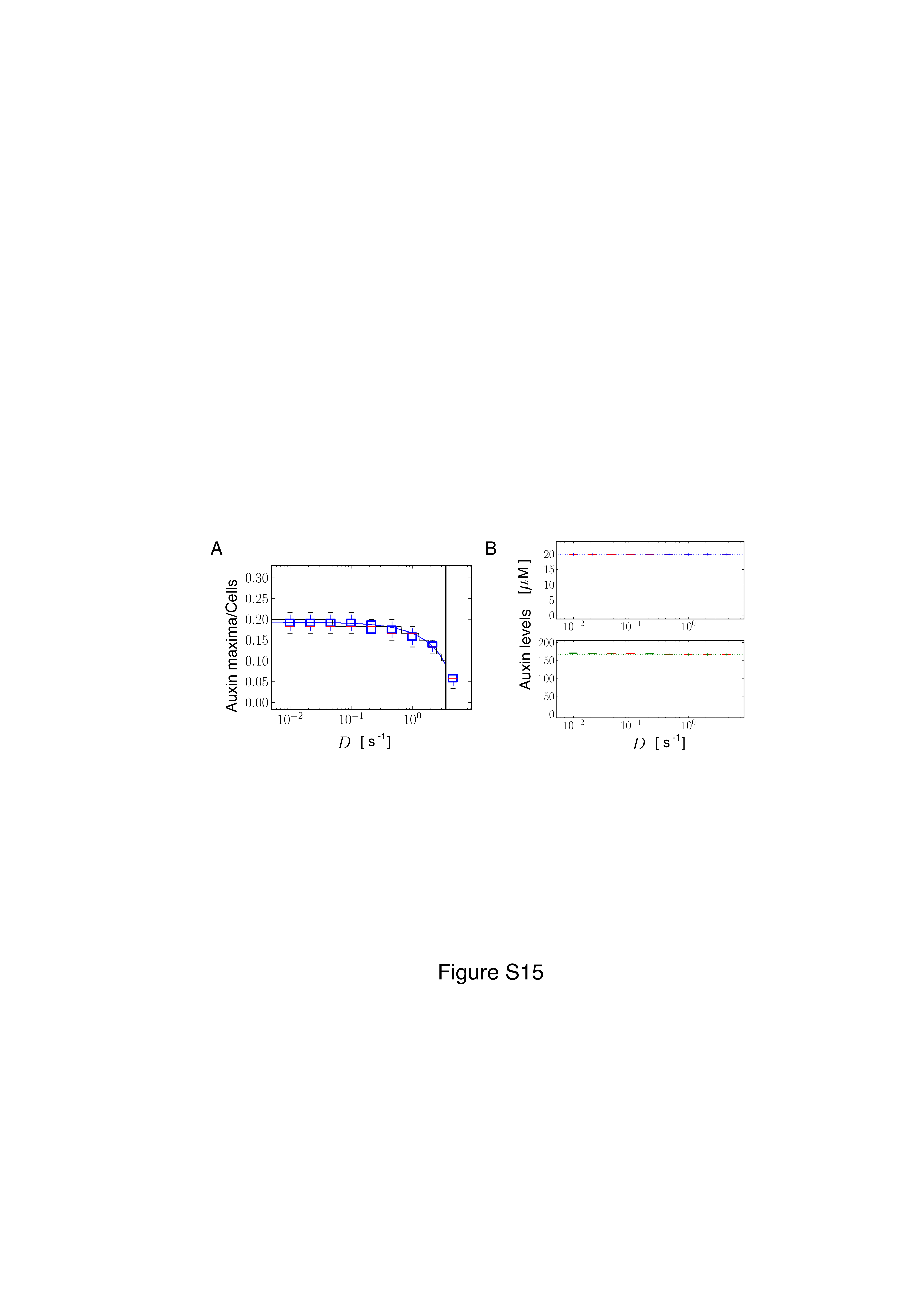

Supplement: S15 Fig — (A) Inverse value of the number of cells between auxin maxima. (B) Average levels of auxin in the cytosol (top, blue boxplot) and in the apoplast (bottom, green boxplot) as a function of the apoplastic diffusion coefficient (D). Results from numerical simulation of the model dynamics are shown by boxplots. Each boxplot depicts the results for 30 simulations with different initial auxin distributions (Materials and Methods) on a ring of 60 cells and 60 apoplastic compartments. Depicted boxplot components are the same as in Fig 1B. Thin solid lines in (A) are obtained from linear stability analysis on a ring of 60 (black) and 1200 (blue) cells, while vertical line indicates the critical apoplastic diffusion value below which the pattern cannot emerge, derived from linear stability analysis. Dotted lines in (B) panels as in Fig 5 (E and I). I = 0.1 μM s-1 and other parameter values are the same as in Fig 1. (TIF) [file pgen.1005183.s015.tif]

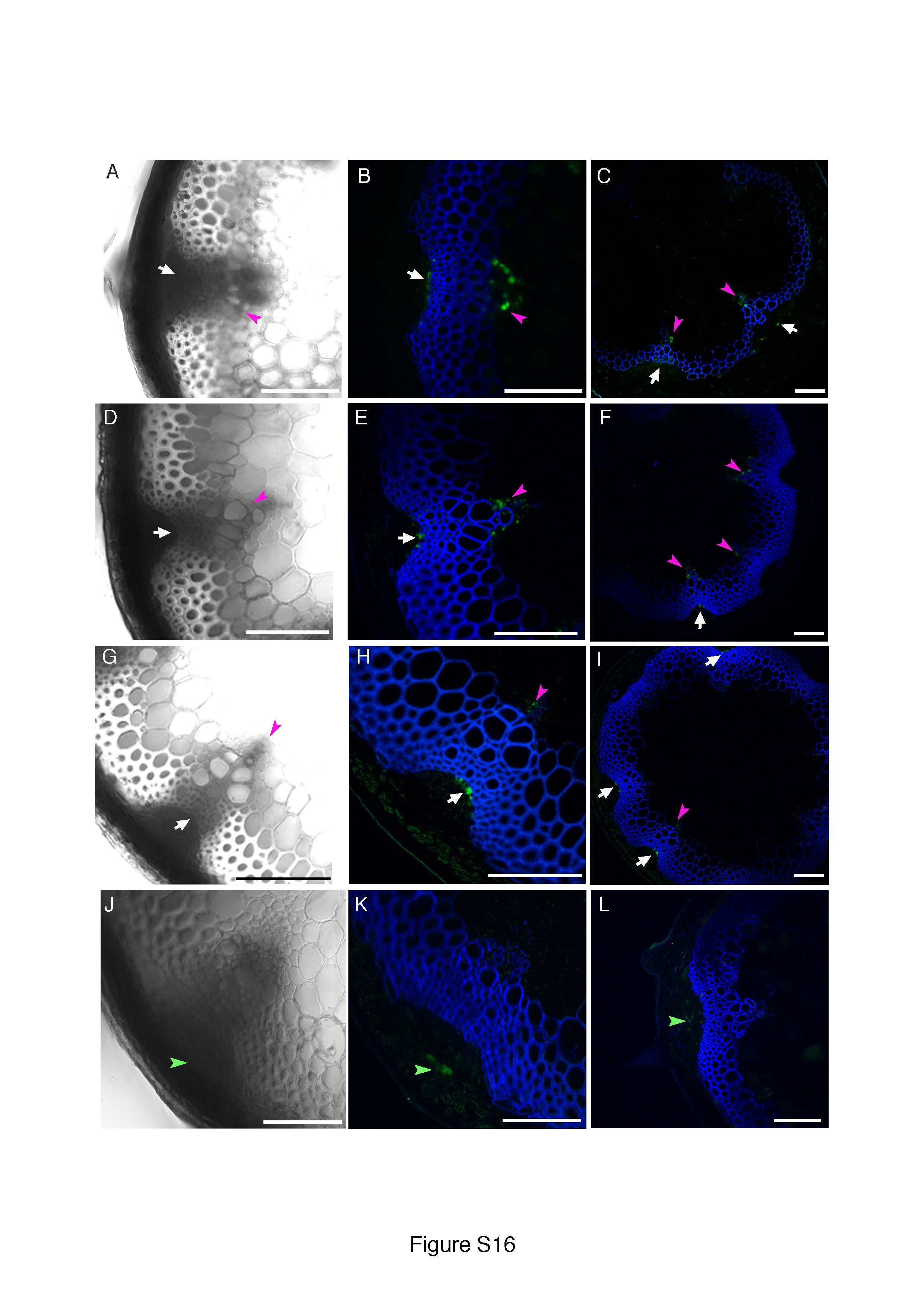

Supplement: S16 Fig — AUX1/LAX-VENUS reporters show localization in procambial, protoxylem and phloem cell files in the vascular bundles of Arabidopsis shoot stems. (A-C) ProAUX1:AUX1::VENUS fluorescence is present in procambial and protoxylem cell files. (D-F) ProLAX1:LAX1::VENUS fluorescence is present in procambial and protoxylem cells. (G-I) ProLAX2:LAX2::VENUS fluorescence is present in procambial and protoxylem cells. (J-L) ProLAX3:LAX3::VENUS fluorescence is present in the phloem cell files. Left panels are transmission channels of the corresponding confocal image in the adjacent middle panel. Blue autofluorescence highlights xylem cells and interfascicular fibers. Pink arrowheads indicate protoxylem cells within the VB. White arrows indicate undifferentiated procambial cells between phloem and xylem cells. Phloem cells are indicated by green arrowhead. All plants were grown for 5 weeks in long day conditions. VENUS fluorescence images were acquired in hand-made sections from the Z1 zone of the stem. Scale bars: 100 μm. (TIF) [file pgen.1005183.s016.tif]
