# Supplementary material for: Auxin Influx Carriers Control Vascular Patterning and Xylem Differentiation in Arabidopsis thaliana
Source: PLoS Genet. 2015 Apr 29;11(4):e1005183. doi: 10.1371/journal.pgen.1005183 (PMC4414528; doi:10.1371/journal.pgen.1005183)
Supplement: S1 Text — (PDF) [file pgen.1005183.s017.pdf]

S1 Text. Auxin influx carriers control vascular patterning and xylem differentiation in *Arabidopsis thaliana*

Norma Fàbregas[1,†], Pau Formosa-Jordan[2,†,#1], Ana Confraria[1,†,#2], Riccardo Siligato[3,4], Jose M. Alonso[5], Ranjan Swarup[6], Malcolm J. Bennett[6], Ari Pekka Mähönen[3,4], Ana I. Caño-Delgado[1,&,*] and Marta Ibañes[2,&,*].

1 Department of Molecular Genetics. Centre for Research in Agricultural Genomics (CRAG) CSIC-IRTA-UAB-UB. Barcelona E-08193, Spain.
2 Department of Structure and Constituents of Matter, Faculty of Physics, University of Barcelona, Barcelona E-08028, Spain.
3 Institute of Biotechnology, University of Helsinki, Helsinki, FIN-00014, Finland.
4 Department of Biosciences, University of Helsinki, Helsinki, FIN-00014, Finland.
5 Department of Plant and Microbial Biology, North Carolina State University, Raleigh, North Carolina 27695, USA.
6 School of Biosciences and Centre for Plant Integrative Biology, University of Nottingham, LE12 5RD Nottingham, United Kingdom.
#1 Current Address: Sainsbury Laboratory, University of Cambridge, Bateman Street, Cambridge, CB2 1LR, United Kingdom.
#2 Current Address: Instituto Gulbenkian de Ciência, 2780-156 Oeiras, Portugal.
∗ E-mails: Corresponding authors marta.ibanes@ub.edu (MI) and ana.cano@cragenomica.es (AICD).
† These authors contributed equally to this work.
& These authors also contributed equally to this work.

Model formulation

In this section, we derive the model equations for auxin transport we used in this work. Auxin transport involves three parts: the transport along the apoplast, the transport across the cell membrane and the transport within a cell. For the transport along the apoplast, we considered passive diffusion and modeled it as in [1] by using their same tissue geometry and by setting cells and apoplasts as single space points (see cell and apoplast array layout in S2B Fig). For the auxin transport across the cell membrane we adopted the model by [2] (originally by [3–6]). One advantage of this model is that it provides a quite realistic approach of the chemiosmotic model of auxin transport, while being still analytically treatable. Regarding the transport inside cells, we simplified it by considering it to be instantaneous. This is a simplification that takes passive diffusion inside cells to be much faster than in the apoplast [7]. Therefore, in our simplified model, once auxin has entered into the cell it can immediately exit to the apoplast by any cell side. The model parameters are related to physicochemical magnitudes that have been measured or can generally be estimated (see S1 Table for parameter estimations, extracted from [3–11]). The chosen 1D layout also facilitates the analytical treatment, and enables a more direct focus on understanding the role of influx in the auxin maxima formation.

As pointed out in Materials and Methods, the dimensional dynamical equations in time τ for the auxin concentration in cell i and in apoplast i, which we denote as A_i and a_i respectively, read

$$\frac{\mathrm{d}A_i}{\mathrm{d}\tau} = -\sum_{j\in n(i)} W_{ij}J_{ij} - \nu_c A_i + \sigma_c \tag{S1}$$

$$\frac{\mathrm{d}a_i}{\mathrm{d}\tau} = \sum_{j\in N(i)} W_{ji}\frac{V_{cell}}{V_{ap}}J_{ji} + D_w\nabla_i^2 a_i\ , \tag{S2}$$

respectively, where J_{ij} is the auxin flux from the cell i to the apoplast j, σ_c and ν_c are the production and degradation rates of auxin within the cell i and D_w is the effective diffusion coefficient of auxin at the apoplast. ∇_i^2 is the discrete Laplacian in a one-dimensional lattice such that $\nabla_i^2 a_i = (a_{i+1} + a_{i-1} - 2a_i)/(\Delta L)^2$, being ΔL the distance between two apoplast units. This Laplacian is the approximated form arising for the simplified tissue array we used in which apoplasts and cells are modeled as single space points adjacent to each other (S2B Fig). The summations in Eqs S1 and S2 are referred to the apoplasts neighboring cell i ($n(i) = i-1, i$) and to the cells neighboring the apoplast i ($N(i) = i, i+1$) (S2B Fig). As defined in [2], W_{ij} is the ratio between the area of the cell membrane i facing apoplast j and the volume of the cell i, while V_{cell} and V_{ap} refer to the cell and apoplast volume, which we assumed to be the same for all cells and apoplasts. L_c is the length of cells and L_a is that of the apoplast in between two cells (S2B Fig). As a result, we have $W_{ij} = 1/L_c$, $W_{ji}\frac{V_{cell}}{V_{ap}} = 1/L_a$ and $\Delta L = L_a + L_c$.

The flux J_{ij} term takes into account the chemiosmotic model. According to it, the protonated auxin (aH) can passively cross the cell membrane, either from the cytoplasm to the apoplast or viceversa. In contrast, the anionic auxin (a^-) can only cross the cell membrane by active transport mediated by influx and efflux carriers. J_{ij} reads [2]

$$J_{ij} = p_{aH}(f^c_{aH}A_i - f^w_{aH}a_j) + p_{pin}W_{ij}P_{ij}N(\phi)y(f^c_{a^-}A_i) - p_{aux}W_{ij}I_{ij}N(\phi)y(f^w_{a^-}a_j)\ , \tag{S3}$$

which is fully described in [2]. For completeness, we detail and describe herein each term. The first term corresponds to the passive transport of protonated auxin across the cell membrane, whereas the second and third terms model the active transport of anionic auxin across the cell membrane through efflux and influx carriers respectively. Accordingly, p_{aH}, p_{pin} and p_{aux} are the permeabilities for protonated auxin passive transport, and for anionic active efflux and influx transport respectively. P_{ij} and I_{ij} are the efflux and influx carriers density in the i cell membrane facing the j apoplast. Since the active transport occurs across a membrane potential V, $N(\phi = z|V|/RT) = \phi e^{\phi}/(e^{\phi} - 1)$ sets the electrochemical factor [3], being z the valence of the ion (herein z=1), R the gas constant, F is Faraday's constant and T is the temperature (herein we take standard temperature conditions, T=298.15 K). $y(x)$ is a function that accounts for the active transport of auxin given a certain amount of carriers. Notice that the active transport in J_{ij} is simplified, based on the standard membrane potential values assigned to plant cells (S1 Table). Specifically, we considered that the active transport from the cytoplasm to the apoplast is only mediated by efflux carriers, whereas the inverse active transport, from the apoplast to the cytoplasm, is only mediated by influx carriers.

The above flux J_{ij} assumes as well that the fraction of anionic and protonated auxin over total auxin concentration inside cells and in the apoplast is constant over time, but distinct in these two compartments: f^c_{aH}, f^w_{aH}, $f^c_{a^-}$ and $f^w_{a^-}$ are the ratios over total auxin of protonated auxin (aH) and anionic auxin (a^-), respectively, at the apoplast (w, or cell wall) and cytosol (c) (S1 Table). These differences rely on the distinct pH condition at the cytoplasm and at the apoplast. Since $f^c_{aH} << f^c_{a^-}$ (S1 Table), auxin exits the cytoplasm mainly through active transport, whereas it can enter into cells either passively or actively ($f^w_{aH} \approx f^w_{a^-}/2$, S1 Table).

Notice that in Eq S3, passive transport across the cell membrane is considered to be linear, non-saturated, whereas an arbitrary auxin-dependent non-dimensional function $y(x)$ for the active transport, the same for both active and influx transport for simplicity, is considered. Despite several results presented

below are obtained for such an arbitrary function y, all our results in figures correspond to the case of linear non-saturated active transport with $y(x) = k_{a^-}x$, being k_{a^-} a constant having units of μM^{-1}. We used linear fluxes for active influx and efflux transport $y(x) = k_{a^-}x$ for simplicity and because our focus of attention is the dynamics at early times during which the linear non-saturated regime dominates. In addition, linear fluxes $y(x) = k_{a^-}x$ have been previously shown to be sufficient to drive periodic patterning if proper nonlinearities in the polarization of efflux carriers ($f(x_i) = x_i^h$) are taken into account [1,3].

The active transport across the cell membrane depends on the densities of efflux and influx carriers, P_{ij} and I_{ij}. Following [2], we considered them to be in equilibrium. These equilibrium values result from carriers production and degradation at the cytosol and from cycling between the cytosol and the cell membrane. Based on experimental evidences [12–14], the production of both efflux and influx is dependent on cytoplasmic auxin concentration in the cell. In addition, the cycling of efflux carriers is also considered to be dependent on cytoplasmic auxin concentrations in adjacent cells [2,3,15]. This last condition defines how efflux carriers become polarized and is crucial for periodic patterning to arise in this model. Taking into account all these dynamics and imposing the equilibrium condition, Sahlin et al. have shown that the efflux and influx densities read [2]

$$P_{ii} = \frac{P_0 P_T(A_i)}{W_{ii}} \frac{f(A_{i+1})}{1 + f(A_{i+1}) + f(A_{i-1})} \tag{S4}$$

$$P_{ii-1} = \frac{P_0 P_T(A_i)}{W_{ii-1}} \frac{f(A_{i-1})}{1 + f(A_{i+1}) + f(A_{i-1})} \tag{S5}$$

and

$$I_{ij} = \frac{I_0 I_T(A_i)}{W_{ij}} \frac{k_{exo}/k_{endo}}{1 + d k_{exo}/k_{endo}} \ , \tag{S6}$$

being $P_0 P_T(A_i)$ and $I_0 I_T(A_i)$ the total concentration of efflux and influx carriers in cell i, which depend on auxin concentration in such cell; P_0 and I_0 are dimensional characteristic concentrations of efflux and influx carriers, whereas $P_T(A_i)$ and $I_T(A_i)$ are the total non-dimensional, normalized to P_0 and I_0, concentrations; d is the number of adjacent cells ($d = 2$ in this circular, one-dimensional with periodic boundary conditions, array of cells and apoplasts) and $f(x_i)$ stands for the ratio between the auxin-dependent exocytosis ($f_{exo}(x_i)$) and endocytosis ($f_{endo}(x_i)$) rates of the efflux carriers, $f(x_i) = \frac{f_{exo}(x_i)}{f_{endo}(x_i)}$ which depend on auxin; k_{exo} and k_{endo} are the constant, auxin-independent, rates for influx carriers exocytosis and endocytosis. Hereafter we define $K_a \equiv \frac{k_{exo}/k_{endo}}{1+2k_{exo}/k_{endo}}$. All cells were assumed to have the same identical rates and auxin-dependent functions.

We defined the non-dimensional time $t = \nu_c \tau$. The dynamics in this non-dimensional time read:

$$\begin{aligned}\frac{\mathrm{d}A_i}{\mathrm{d}t} &= -\epsilon \tilde{D}_{ca} \left(z f^c_{aH} A_i - f^w_{aH} \sum_{j \in n(i)} a_j \right) - \epsilon \tilde{E} \frac{y(f^c_{a^-} A_i) P_T(A_i) \sum_{k \in nn(i)} f(A_k)}{1 + \sum_{k \in nn(i)} f(A_k)} + \\ &\quad + \epsilon \tilde{I} I_T(A_i) \sum_{j \in n(i)} y(f^w_{a^-} a_j) + \sigma - A_i \end{aligned} \tag{S7}$$

$$\begin{aligned}\frac{\mathrm{d}a_i}{\mathrm{d}t} &= \tilde{D}_{ca} \left(\left(\sum_{j \in N(i)} f^c_{aH} A_j \right) - z f^w_{aH} a_i \right) + \tilde{E} \sum_{j \in N(i)} \frac{P_T(A_j) y(f^c_{a^-} A_j) f(A_{j^\dagger})}{1 + \sum_{k \in nn(j)} f(A_k)} - \\ &\quad - \tilde{I} y(f^w_{a^-} a_i) \sum_{j \in N(i)} I_T(A_j) + \tilde{D} \widetilde{\nabla^2}_i a_i \ , \end{aligned} \tag{S8}$$

where $\widetilde{\nabla^2}_i a_i = \sum_{k \in nn(j)} (a_k - a_i)$, $k \in nn(i)$ refers to cells that are nearest neighbors from cell i, and $j^\dagger$ refers to the other cell that neighbors the apoplast (when $j = i$ then $j^\dagger = i + 1$ and when $j = i + 1$ then

$j^\dagger = i$). The parameters in these dynamics are defined as $\sigma = \sigma_c/\nu_c$, $\epsilon = L_a/L_c$, $\tilde{D} = D_w/\nu_c \Delta L^2$ with $\Delta L = (L_a + L_c)$, $\tilde{D}_{ca} = D_{ca}/\nu_c$ with $D_{ca} = p_{aH}/L_a$, $\tilde{E} = E/\nu_c$ with $E = p_{pin} P_0 N(\phi)/L_a$, and $\tilde{I} = I/\nu_c$ with $I = p_{aux} I_0 K_a N(\phi)/L_a$. While during the analytic derivations we are using parameters $\tilde{E}$, $\tilde{I}$, $\tilde{D}_{ca}$ and $\tilde{D}$ for simplicity, note that for referring to results and in the different figures we will use parameters E, I and D_{ca} and D, being $D = D_w/\Delta L^2$.

We numerically integrated these dynamics in non-dimensional units of time with active linear auxin fluxes $y(x_i) = k_{a^-} x_i$, non-linear auxin-dependent polarization of efflux carriers $f(x_i) = x_i^h$, and auxin-induced saturating synthesis of carriers $P_T(x) = \frac{x}{x+\theta_P}$ and $I_T(x) = \frac{1}{2}\frac{x}{x+\theta_I}$. In these simulations, we have considered that all the efflux carriers are in the membrane and not in the cytosol, so that $1 + \sum_{k\in nn(j)} f(A_k) \approx \sum_{k\in nn(j)} f(A_k)$ (see below). Parameter values used are shown in S1 Table. Additional parameter values used, unless otherwise stated, are $\theta_P = \theta_I = 10\ \mu M$, $k_{a^-} = 1\ \mu M^{-1}$, $h = 2$, $E = 105\ \mu M\ \mathrm{s}^{-1}$, $D_{ca} = 15\ \mathrm{s}^{-1}$, $D = 2\ \mathrm{s}^{-1}$ and $\epsilon = 0.05$.

Linear stability analysis (LSA)

In this section we study the pattern formation capabilities of our system through a linear stability analysis (LSA) over the homogeneous precursor state of the dynamical system described by Eqs S7 and S8. Details about this method can be found elsewhere (see for instance [2, 3, 16]). Our analysis is generic for any functions f, y, P_T and I_T.

We set A_0 and a_0 as the concentrations of auxin at the cytosol and at the apoplast respectively in the homogeneous precursor fixed point, which is defined by $\frac{\mathrm{d}A_i}{\mathrm{d}t} = \frac{\mathrm{d}A_j}{\mathrm{d}t} = \frac{\mathrm{d}A_0}{\mathrm{d}t} = 0$, $\frac{\mathrm{d}a_i}{\mathrm{d}t} = \frac{\mathrm{d}a_j}{\mathrm{d}t} = \frac{\mathrm{d}a_0}{\mathrm{d}t}\ \forall\ i, j$. For Eqs S7 and S8, the homogeneous fixed point verifies:

$$A_0 = \frac{\sigma_c}{\nu_c} \tag{S9}$$

$$0 = \tilde{D}_{ca} \left(f_{aH}^c A_0 - f_{aH}^w a_0 \right) + \tilde{E} \frac{P_T(A_0) y(f_{a^-}^c A_0) f(A_0)}{1 + d f(A_0)} - \tilde{I} y(f_{a^-}^w a_0) I_T(A_0)\ . \tag{S10}$$

LSA evaluates the dynamics of small perturbations $\hat{A}_i$ and $\hat{a}_i$ applied on the homogeneous state ($|\hat{A}_i| << A_0$ and $|\hat{a}_i| << a_0$). By introducing $A_i = A_0 + \hat{A}_i$ and $a_i = a_0 + \hat{a}_i$ into Eqs S7 and S8 and doing a Taylor development until first order around the homogeneous fixed point, we obtain the linearized system for the dynamics of these perturbations, which reads ($'$ stands for the first derivative with respect to the auxin variable):

$$\frac{\mathrm{d}\hat{A}_i}{\mathrm{d}t} = -(\epsilon C_1 + 1)\hat{A}_i - \epsilon C_2 \sum_{k\in nn(i)} \hat{A}_k + \epsilon C_3 \sum_{j\in n(i)} \hat{a}_j \tag{S11}$$

$$\frac{\mathrm{d}\hat{a}_i}{\mathrm{d}t} = C_4 \sum_{j\in N(i)} \hat{A}_j - C_5 \sum_{j\in N(i)} \sum_{k\in nn(j)} \hat{A}_k - C_6 \hat{a}_i + \tilde{D} \sum_{j\in nn(i)} \hat{a}_j\ . \tag{S12}$$

where

$$\begin{aligned}
C_1 &= d\tilde{E}b_0 + df^c_{aH}\tilde{D}_{ca} - d\tilde{I}I'_T(A_0)y(f^w_{a^-}a_0) && \text{(S13)}\\
C_2 &= \tilde{E}b_1(b_2 - db_3) && \text{(S14)}\\
C_3 &= \tilde{I}I_T(A_0)f^w_{a^-}y'(f^w_{a^-}a_0) + f^w_{aH}\tilde{D}_{ca} && \text{(S15)}\\
C_4 &= f^c_{aH}\tilde{D}_{ca} + \tilde{E}(b_0 + b_1b_2) - y(f^w_{a^-}a_0)\tilde{I}I'_T(A_0) && \text{(S16)}\\
C_5 &= \tilde{E}b_1b_3 && \text{(S17)}\\
C_6 &= d(f^w_{a^-}y'(f^w_{a^-}a_0)\tilde{I}I_T(A_0) + \tilde{D}_{ca}f^w_{aH} + \tilde{D})\,, && \text{(S18)}
\end{aligned}$$

and

$$\begin{aligned}
b_0 &= \frac{f(A_0)}{1+df(A_0)}\left((f^c_{a^-}y'(f^c_{a^-}A_0)P_T(A_0) + y(f^c_{a^-}A_0)P'_T(A_0)\right) && \text{(S19)}\\
b_1 &= \frac{y(f^c_{a^-}A_0)P_T(A_0)}{(1+df(A_0))^2} && \text{(S20)}\\
b_2 &= f'(A_0)(1+df(A_0)) && \text{(S21)}\\
b_3 &= f(A_0)f'(A_0)\,. && \text{(S22)}
\end{aligned}$$

The small perturbations $\hat{A}_j$ and $\hat{a}_j$ in a system of N cells can be written in Fourier series as

$$\hat{A}_j = \sum_{\bar{q}} \mu_{\bar{q}} e^{2\pi i \bar{q} j}, \quad \hat{a}_j = \sum_{\bar{q}} \rho_{\bar{q}} e^{2\pi i \bar{q} j}\ , \tag{S23}$$

being $\bar{q} = q/N$ for the wavenumber $q = \{1, ..., N-1, N\}$ and then $\bar{q} \in [1/N, 1]$. The inverse transforms read

$$\mu_{\bar{p}} = \frac{1}{N}\sum_{j=1}^{N} \hat{A}_j e^{-2\pi i \bar{p} j}, \quad \rho_{\bar{p}} = \frac{1}{N}\sum_{j=1}^{N} \hat{a}_j e^{-2\pi i \bar{p} j}\ , \tag{S24}$$

and $\mu_{\bar{p}}$ and $\rho_{\bar{p}}$ correspond to the amplitudes of perturbations with spatial periodicity $\bar{p}$ in the cytosol and the apoplast, respectively.

By introducing the transforms set by Eqs S23 into the linearized system (Eqs S11 and S12) we get

$$\frac{\mathrm{d}}{\mathrm{d}t}\begin{pmatrix} \mu_{\bar{q}} \\ \rho_{\bar{q}} \end{pmatrix} = M \begin{pmatrix} \mu_{\bar{q}} \\ \rho_{\bar{q}} \end{pmatrix}\ ,$$

where M is a matrix that reads

$$\begin{pmatrix} -\psi_0 & \psi_1 \\ \psi_2 & \psi_3 \end{pmatrix}\ ,$$

being

$$\begin{aligned}
\psi_0 &= \epsilon(C_1 + 2C_2\cos(2\pi\bar{q})) + 1 && \text{(S25)}\\
\psi_1 &= \epsilon C_3(1 + e^{-i2\pi\bar{q}}) && \text{(S26)}\\
\psi_2 &= C_4(1 + e^{i2\pi\bar{q}}) - C_5(1 + 2\cos(2\pi\bar{q}) + e^{i4\pi\bar{q}}) && \text{(S27)}\\
\psi_3 &= 2\tilde{D}\cos(2\pi\bar{q}) - C_6\,. && \text{(S28)}
\end{aligned}$$

Accordingly, by finding the eigenvalues of matrix M, the dynamics of the perturbations $\hat{A}_i$ and $\hat{a}_i$ become readily solved. The eigenvalues of the M matrix are

$$\alpha_{\bar{q}} = \frac{1}{2}\left(Tr_{\bar{q}} \pm \sqrt{Tr^2_{\bar{q}} - 4\,det_{\bar{q}}}\right)\ , \tag{S29}$$

where subscript $\bar{q}$ has been included to stress that there are two eigenvalues for each period $\bar{q}$, being $Tr_{\bar{q}}$ and $det_{\bar{q}}$ the trace and the determinant of the M matrix respectively, which read

$$Tr_{\bar{q}} = \psi_3 - \psi_0 \quad \text{(S30)}$$

$$det_{\bar{q}} = -(\psi_0\psi_3 + \psi_1\psi_2) \; . \quad \text{(S31)}$$

The homogeneous state is linearly unstable when $\alpha_{\bar{q}} > 0$ for at least one wavenumber mode $\bar{q}$. This sets the condition for pattern formation. By calculating the fastest growing mode (hereafter named κ), i.e. the $\bar{q}$ value (κ) that maximizes $\alpha_{\bar{q}}$, the condition for pattern formation (i.e. having the homogeneous state linearly destabilized) becomes $\alpha_\kappa > 0$. The boundary between regions where the homogeneous state is linearly stable and where it is not is computed by setting $\alpha_\kappa = 0$.

Linear stability analysis results presented in the manuscript correspond to the computation of the pattern formation condition numerically (i.e. $\alpha_{\bar{q}} > 0$) for at least one $\bar{q}$ value) for linear active auxin carriers-mediated transport $y(x) = k_{a^-}x$, nonlinear auxin-dependent polarization of efflux carriers $f(x) = x^h$, auxin-dependent saturating functions of carriers synthesis $P_T(x) = \frac{x}{x+\theta_P}$ and $I_T(x) = \frac{1}{2}\frac{x}{x+\theta_I}$, and all efflux carriers being at the membrane and not in the cytosol (i.e. $1 << df(A_0)$). The condition of all efflux carriers being located at the membrane makes, for instance, $C_2 = 0$, which simplifies the computations (for more details about this simplification see for instance [1,3]). The value of the fastest growing mode κ was also computed numerically and shown in the phase diagrams and in together with simulation results shown in boxplots. The exact boundary for pattern formation condition ($\alpha_\kappa = 0$) is plotted with a continuous line in parameter space shown in Figs 1C, S4C and S12B, and as a vertical solid line in the boxplots shown in Figs 5F, S14F and S15A.

We also analyzed numerically the simplified case in which the total amount of efflux and influx carriers is constant over time and the same for all cells and thus they do not depend on the amount of auxin ($P_T = 1$ and $I_T = 1/2$, by setting $\theta_P = \theta_I = 0$). Results are shown in S4 Fig. The same qualitative results as for auxin-dependent synthesis of carriers were obtained.

Analytical expressions for pattern formation

Analytical expressions for pattern formation can be obtained from the above Eqs S25-S31 when efflux carriers are just in the membrane (i.e. so that $1 + df(A_0) \approx df(A_0)$) and efflux and influx carriers concentrations are constant in the cell ($P_T = 1$ and $I_T = 1/2$, by setting $\theta_P = \theta_I = 0$). In this case, it can be shown that the trace $Tr_{\bar{q}}$ is negative for all parameter values ($Tr_{\bar{q}} < 0$). Since $\alpha_{\bar{q}}$ is given by Eq S29, and taking into account that $Tr_{\bar{q}} < 0$, the condition for pattern formation, i.e. $\alpha_{\bar{q}} > 0$ for at least one $\bar{q}$ value, simplifies to the condition $det_{\bar{q}} < 0$. Note that this is fulfilled for any monotonically increasing auxin flux set by $y(x)$ and any auxin-dependent polarization of efflux carriers function $f(x)$. We can then obtain an analytical expression for the fastest growing mode κ by imposing the condition of extreme $\frac{\partial \alpha_{\bar{q}}}{\partial \bar{q}} = 0$. When developed, this condition of extreme results into a very large expression of little practical use for analytical studies. Accordingly, we chose to write down an approximate expression for κ that was analytically treatable. The approximation was done as follows.

The approximation we performed is to focus on the wavenumber that minimizes $det_{\bar{q}} < 0$ (hereinafter named $\kappa^* \equiv \bar{q}*$), instead of focusing on the fastest growing mode κ (which is the one one maximizing $\alpha_{\bar{q}}$). It was assumed that $\kappa^* \approx \kappa$. This approximation is justified when $Tr_{\bar{q}} < 0$, since in this case the condition $\alpha_{\bar{q}}$ is satisfied if and only if $det_{\bar{q}} < 0$ as indicated above. The results obtained by this approximation can be compared to the exact, numerically computed, ones. This was done for $y(x) = k_{a^-}x$ and $f(x) = x^h$ and is presented in S4 Fig. For $y(x) = k_{a^-}x$ and $f(x) = x^h$, the derivation of $\frac{\partial det_{\bar{q}}}{\partial \bar{q}} = 0$ to obtain the analytical expression of κ^* yields

$$\kappa^* = \frac{1}{2\pi} arccos(\Omega^*) \; , \quad \text{(S32)}$$

being

$$\Omega^* = \frac{4D(\nu_c + \epsilon g_0) + \epsilon g_0 g_1}{2E\epsilon f^c_{a^-} k_{a^-} h g_1} \ , \tag{S33}$$

where $g_0 = \left(f^c_{a^-} k_{a^-} E + 2 f^c_{aH} D_{ca}\right)$ and $g_1 = \left(f^w_{a^-} k_{a^-} I + 2 f^w_{aH} D_{ca}\right)$. This analytical expression is represented as a dashed line in S4B Fig, and shows a very good agreement with the exact, numerically computed, fastest growing mode κ (solid lines). This shows that the assumption $\kappa^* \approx \kappa$ is correct and hence the analytical expressions set by Eqs S32-S33 can be used to extract information about the exact fastest growing mode κ.

By using that $\kappa^* \approx \kappa$, we can define an approximate condition for pattern formation being $det_{\bar{\kappa}^*} < 0$. Notice that the exact condition for pattern formation is $det_{\bar{\kappa}} < 0$. By introducing Eqs S32 and S33 into $det_{\bar{\kappa}^*} < 0$, we obtained the following approximate analytical condition for pattern formation:

$$D \ < \ \frac{1}{4g_2}\left(\epsilon g_1 \left(E f^c_{a^-} k_{a^-} (2h-1) - 2D_{ca} f^c_{aH}\right) + g_1 \sqrt{2^3 \nu_c E \epsilon f^c_{a^-} k_{a^-} h}\right) \ , \tag{S34}$$

where $g_2 = (\nu_c + \epsilon g_0)$. The boundary of this condition (i.e. the equality) is plotted by a dashed line in S4 Fig. Comparison with the exact numerically computed condition (solid line) indicates it is a good approximate condition.

These analytical expressions are also plotted in Fig 1B,C. They are in qualitative agreement with the exacts results, numerically computed, of LSA corresponding to the same situation but with auxin-induced synthesis of carriers (i.e. $P_T(x) = \frac{x}{x+\theta_P}$, $I_T(x) = \frac{1}{2}\frac{x}{x+\theta_I}$, $y(x) = k_{a^-} x$, $f(x) = x^h$ and $1 + df(A_0) \approx df(A_0)$). Therefore, the analytical approximations obtained here are also useful expressions to understand the results arising in the model when the concentration of influx and efflux carriers is not constant but depends on auxin.

Dependence of the periodicity of the pattern and the average concentration of auxin on the amount of influx carriers

Numerical analysis of the dynamics shows that the average concentration of auxin at the cytoplasm and at the apoplast in a periodic pattern depends on influx carriers in a similar way as the homogeneous state of auxin at the cytoplasm and at the apoplast, respectively, does (Figs 5, S13, S14 and S15). This average concentration corresponds to the spatial mean of auxin concentration over all cells (for cytosolic auxin) and over all apoplasts (for apoplastic auxin). In addition, the concentration of apoplastic (but not cytosolic) auxin at minima and at maxima of the pattern depends similarly on the amount of influx carriers as the average apoplastic auxin concentration does (Figs 5, S13 and S14). To have a first approximation of how average auxin concentrations change with the amount of influx carriers, we analyzed the homogeneous steady state of the dynamics, which is given by Eqs S9 and S10. From Eq S9 we see that cytoplasmic auxin on the homogenous state does not depend on the influx carriers $\tilde{I}$ nor on efflux carriers $\tilde{E}$. From Eq S10 we calculated the auxin concentration at the apoplast when the active fluxes are linear, i.e. $y(x) = k_{a^-} x$. This yields:

$$a_0 = A_0 \frac{\left(D_{ca} f^c_{aH} + E\frac{P_T(A_0) f^c_{a^-} k_{a^-} f(A_0)}{1+df(A_0)}\right)}{\left(D_{ca} f^w_{aH} + I f^w_{a^-} k_{a^-} I_T(A_0)\right)} \ . \tag{S35}$$

By using the values of S1 Table (and $d = 2$ and $k^-_a = 1$), we can obtain $a_0 \approx 3A_0 \frac{EP_T(A_0)f(A_0)/(1+2f(A_0))}{D_{ca}+2II_T(A_0)}$. These expressions show that the apoplastic auxin concentration in the homogeneous state depends oppositely on influx and efflux carriers. In particular, the concentration of auxin at the apoplast in the homogeneous state increases with efflux carriers or when the amount of influx carriers decreases.

According to Eqs S32 and S33, the wavenumber and therefore the periodicity of the pattern depends on the amount of influx carriers, changing when $I \approx D_{ca}$. These analytical expressions and their representation predict that influx carriers promote more auxin maxima, specially at low D_{ca} rates and at high apoplastic diffusion coefficient D. By using the values in S1 Table (and $h = 2$ and $k_a^- = 1$), Eq S33 becomes $\Omega^* \approx \frac{3D(1+\frac{E}{2})}{E(I+D_{ca})} + \frac{1}{4}$. Therefore, by comparing this expression with the approximate expression for homogeneous apoplastic auxin $a_0 \approx 3A_0 \frac{EP_T(A_0)f(A_0)/(1+2f(A_0))}{D_{ca}+2II_T(A_0)}$, we see that the wavenumber is less sensitive to the influx carriers than the homogenous, and thereby average, apoplastic concentration (i.e., $\Omega*$ has a term 1/4 that is independent of influx carriers). In other words, the effect of influx carriers on the average apoplastic auxin concentration is more pervasive than on the periodicity of the pattern. The periodicity of the pattern becomes almost insensitive to the effect of influx carriers (i.e., Ω^* and therefore κ^* become independent of I) when the apoplastic diffusion is strongly diminished ($D << (I + D_{ca})$, yielding $\Omega^* \approx \frac{1}{4}$ when having $E \approx 105\ \mu M$ s^{-1}) or when the passive transport across the cell membrane increases ($D_{ca} >> I$, yielding $\Omega^* \approx 3D(1 + E/2)/ED_{ca} + 1/4$, which is $\Omega^* \approx 1/4$ for $D = 2\ s^{-1}$, $E = 105$ μM s^{-1} and $D_{ca} = 50\ s^{-1}$). These two situations are consistent with the exact results of the model, as shown in Figs 1 and 5, respectively. In addition, we see that, in these regimes, the periodicity of the pattern is little sensitive to efflux carriers, which is a good approximation of the exact results of the model as shown in S12 Fig.

References

1. Ibañes M, Fàbregas N, Chory J, Caño-Delgado A (2009) Brassinosteroid signaling and auxin transport are required to establish the periodic pattern of arabidopsis shoot vascular bundles. P Natl Acad Sci USA 106: 13630.

2. Sahlin P, Söderberg B, Jönsson H (2009) Regulated transport as a mechanism for pattern generation: capabilities for phyllotaxis and beyond. J Theor Biol 258: 60–70.

3. Jönsson H, Heisler M, Shapiro B, Meyerowitz E, Mjolsness E (2006) An auxin-driven polarized transport model for phyllotaxis. P Natl Acad Sci USA 103: 1633.

4. Swarup R, Kramer E, Perry P, Knox K, Leyser H, et al. (2005) Root gravitropism requires lateral root cap and epidermal cells for transport and response to a mobile auxin signal. Nat Cell Biol 7: 1057–1065.

5. Heisler M, Jönsson H (2006) Modeling auxin transport and plant development. J Plant Growth Regul 25: 302–312.

6. Kramer E (2004) Pin and aux/lax proteins: their role in auxin accumulation. Trends Plant Sci .

7. Rutschow HL, Baskin TI, Kramer EM (2011) Regulation of solute flux through plasmodesmata in the root meristem. Plant Physiol 155: 1817–26.

8. Goldsmith M, Goldsmith T, Martin M (1981) Mathematical analysis of the chemiosmotic polar diffusion of auxin through plant tissues. P Natl Acad Sci USA 78: 976.

9. Kramer E, Frazer N, Baskin T (2007) Measurement of diffusion within the cell wall in living roots of arabidopsis thaliana. J Exp Bot 58: 3005-3015.

10. Alim K, Frey E (2010) Quantitative predictions on auxin-induced polar distribution of pin proteins during vein formation in leaves. Eur Phys J E 33: 165-173.

11. Kramer EM (2007) Computer models of auxin transport: a review and commentary. J Exp Bot 59: 45–53.

12. Heisler MG, Ohno C, Das P, Sieber P, Reddy GV, et al. (2005) Patterns of auxin transport and gene expression during primordium development revealed by live imaging of the arabidopsis inflorescence meristem. Curr Biol 15: 1899–911.

13. Swarup K, Benková E, Swarup R, Casimiro I, Péret B, et al. (2008) The auxin influx carrier lax3 promotes lateral root emergence. Nat Cell Biol 10: 946–54.

14. Péret B, Middleton AM, French AP, Larrieu A, Bishopp A, et al. (2013) Sequential induction of auxin efflux and influx carriers regulates lateral root emergence. Mol Syst Biol 9: 699.

15. Robert S, Kleine-Vehn J, Barbez E, Sauer M, Paciorek T, et al. (2010) Abp1 mediates auxin inhibition of clathrin-dependent endocytosis in arabidopsis. Cell 143: 111–21.

16. Cross M, Greenside H (2009) Pattern formation and dynamics in nonequilibrium systems. New York: Cambridge University Press.

17. Formosa-Jordan P (2013) Pattern formation through lateral inhibition mediated by Notch signaling. University of Barcelona, Barcelona.
