# Supplementary material for: Auxin Influx Carriers Control Vascular Patterning and Xylem Differentiation in Arabidopsis thaliana
Source: PLoS Genet. 2015 Apr 29;11(4):e1005183. doi: 10.1371/journal.pgen.1005183 (PMC4414528; doi:10.1371/journal.pgen.1005183)
Supplement: S1 Table — (PDF) [file pgen.1005183.s018.pdf]

S1 Table . Table of parameters values ranges related to auxin transport

Parameter (*)	Definition	Value	Reference
V	membrane potential	$-100mV$	[8] (Ex)
f^c_{aH}	fraction AH/A in cells	0.003	[8] (Ex)
f^w_{aH}	fraction AH/A in apoplast	0.334	[8] (Ex)
$f^c_{a^-}$	fraction A^-/A in cells	0.997	[8] (Ex)
$f^w_{a^-}$	fraction A^-/A in apoplast	0.666	[8] (Ex)
p_{aH}	membrane permeability AH	$3.3 \times 10^1\ \mu m s^{-1}$	[8] (Ex)
		$0.389 - 0.5\ \mu m s^{-1}$	[4] (Ex)
D_w	auxin diffusion in walls	$1.8 - 46\ \mu m^2 s^{-1}$	[9] (Ex)
σ_c	auxin production	$2\,\mu M\,s^{-1}$	– (**)
ν_c	auxin degradation	$0.1\,s^{-1}$	[3] (T)
p_{pin}	efflux carrier permeability	$0.0278 - 0.278\,\mu m\,s^{-1}$	[4] (T,Ex)
		$1.39\,\mu m\,s^{-1}$	[6] (T,Ex)
p_{aux}	influx carrier permeability	$0.03 - 0.56\,\mu m\,s^{-1}$	[4] (T,Ex)
		$0.56\,\mu m\,s^{-1}$	[6] (T,Ex)
L_a	apoplast width	$0.05\,\mu m$	[3], [11] (T)
L_c	typical cell length	$5\,\mu m$	– (***)
$\epsilon = L_a/L_c$	-	0.05	
$\Delta l = (L_a + L_c)$	characteristic lattice length	$1.05\,\mu m$	
$N(\phi)$	electrochemical factor for transport across a membrane	3.97	
$I = N(\phi) p_{aux} K_a I_0/L_a$	influx intensity	–	
$E = p_{pin} N(\phi) P_0/L_a$	efflux intensity	–	
$D_{ca} = p_{aH}/L_a$	diffusion rate of protonated auxin	$7.78 - 660\,s^{-1}$	
$D = D_w/\Delta l^2$	apoplastic diffusion rate	$1.6 - 41.7\,s^{-1}$	

(*) Above the horizontal line, parameters inferred from experimental measurements (Ex) or just used in previous models (T). Below, combined and effective parameter ranges and values for the complete model, obtained from the above parameters. In our simulations we used parameters within these ranges (see in Text S1).
(**) We do not know the auxin synthesis rate, but we take this approximate value so that it gives reasonable cytosolic concentrations.
(***) We use this value as an approximation, since we do not know the cell length when the auxin pattern starts forming.
