# Supplementary figures and images for: Correction: Auxin Influx Carriers Control Vascular Patterning and Xylem Differentiation in Arabidopsis thaliana
Source: PLoS Genet. 2015 Jun 17;11(6):e1005296. doi: 10.1371/journal.pgen.1005296 (PMC4470512; doi:10.1371/journal.pgen.1005296)

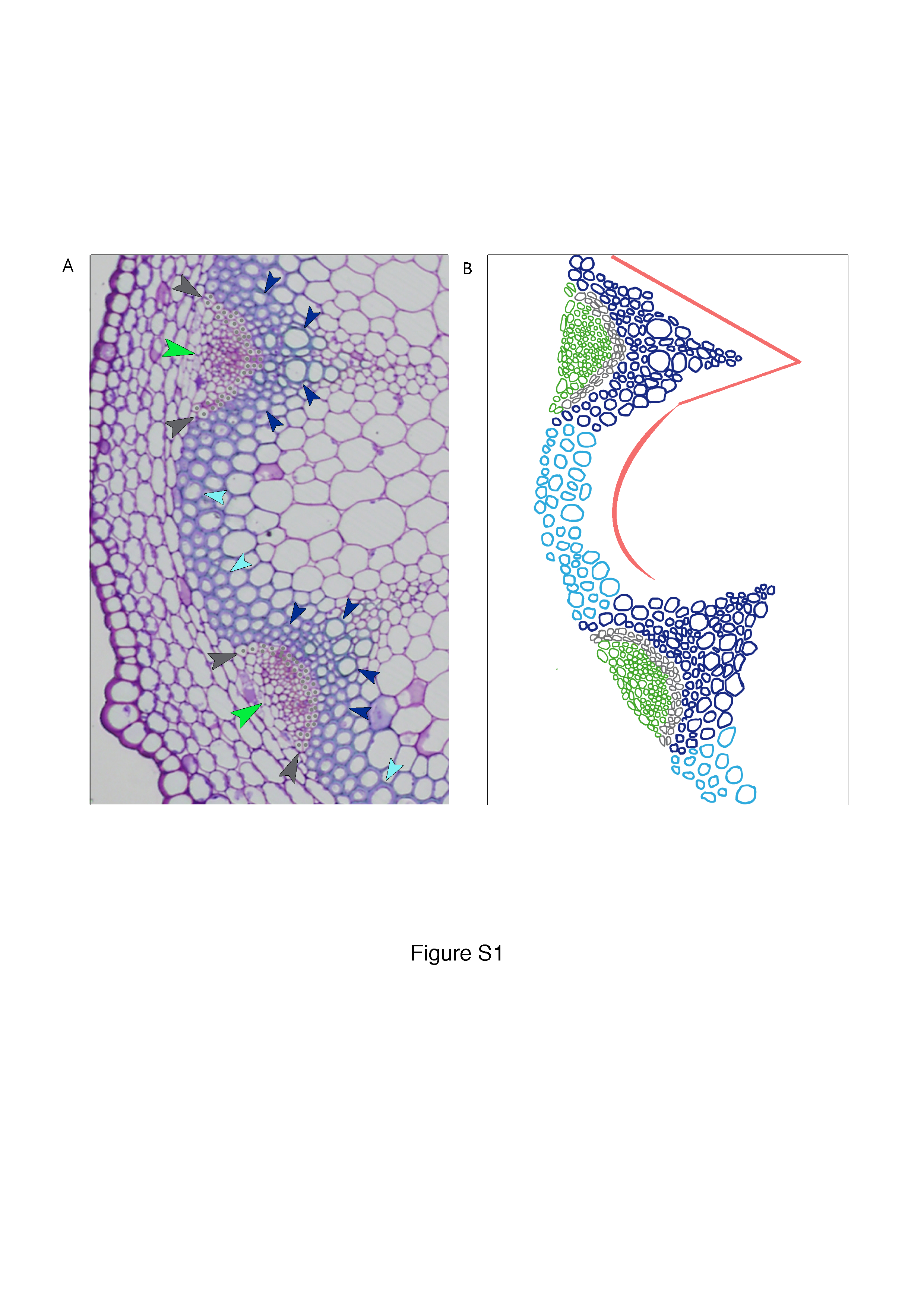

Supplement: S2 File — (ZIP) [file pgen.1005296.s002.zip › Figure S1_new!!.tiff]

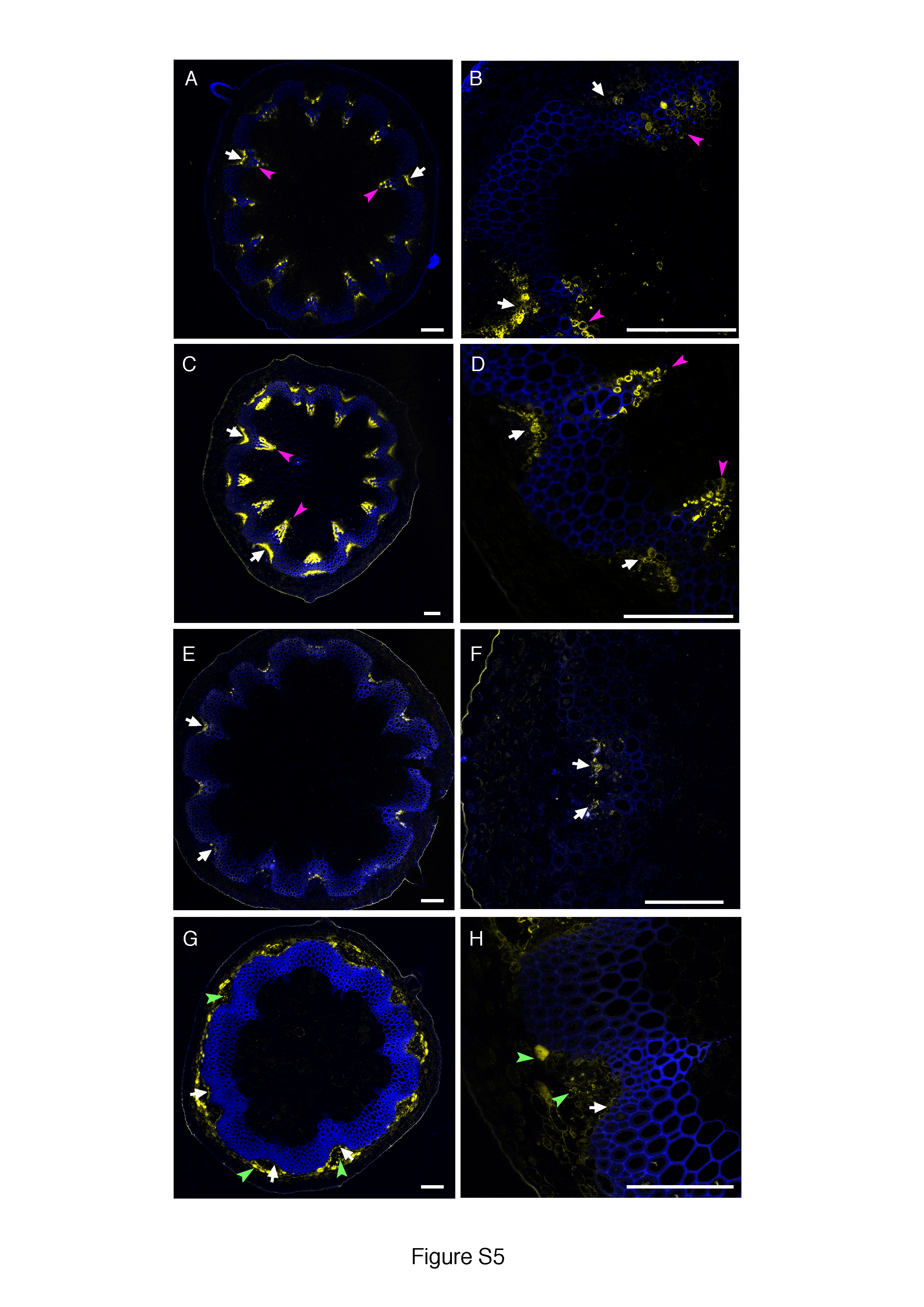

Supplement: S2 File — (ZIP) [file pgen.1005296.s002.zip › Figure S5_V5.tiff]

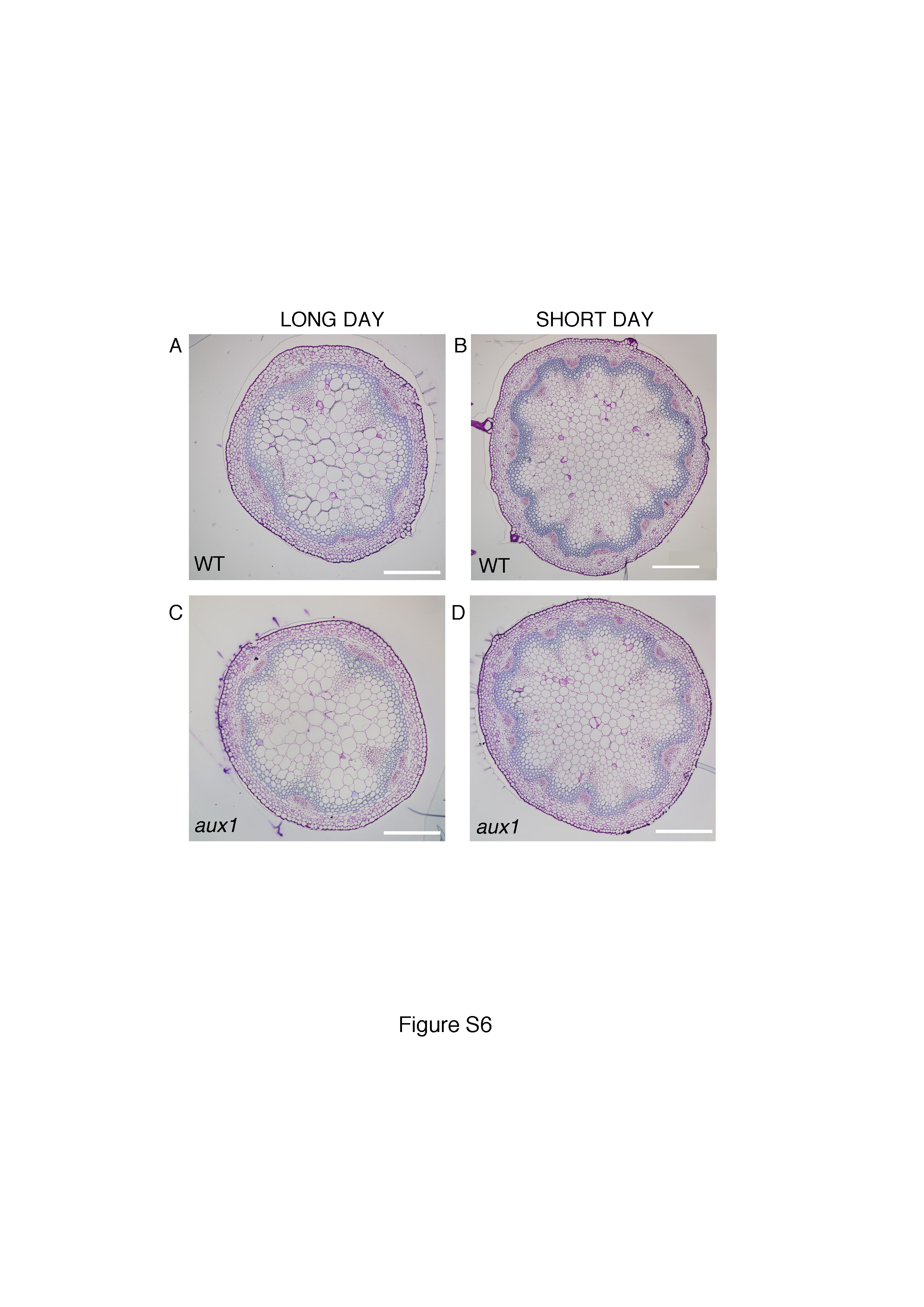

Supplement: S2 File — (ZIP) [file pgen.1005296.s002.zip › Figure S6_new!.tiff]

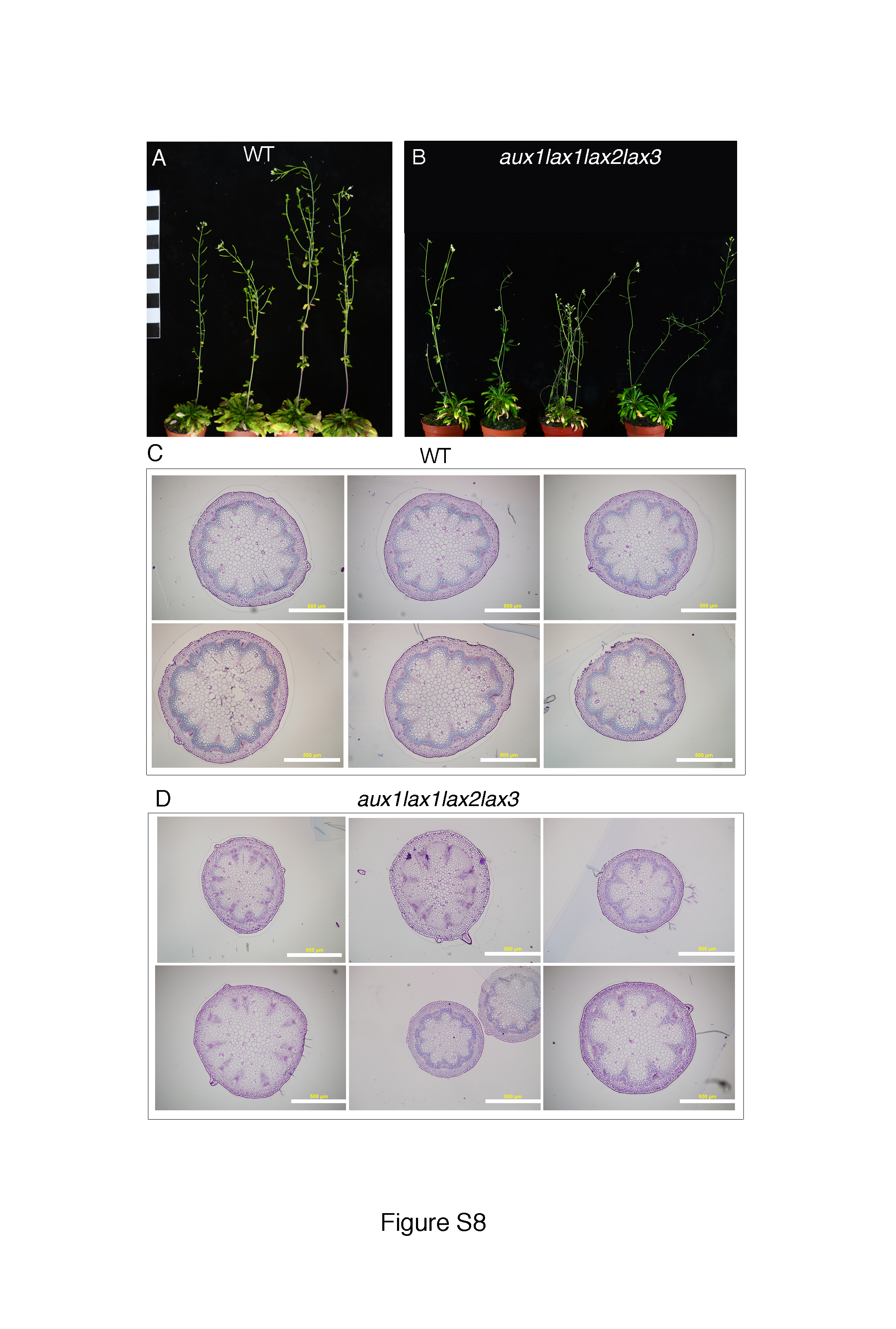

Supplement: S2 File — (ZIP) [file pgen.1005296.s002.zip › Figure S8_new.tiff]

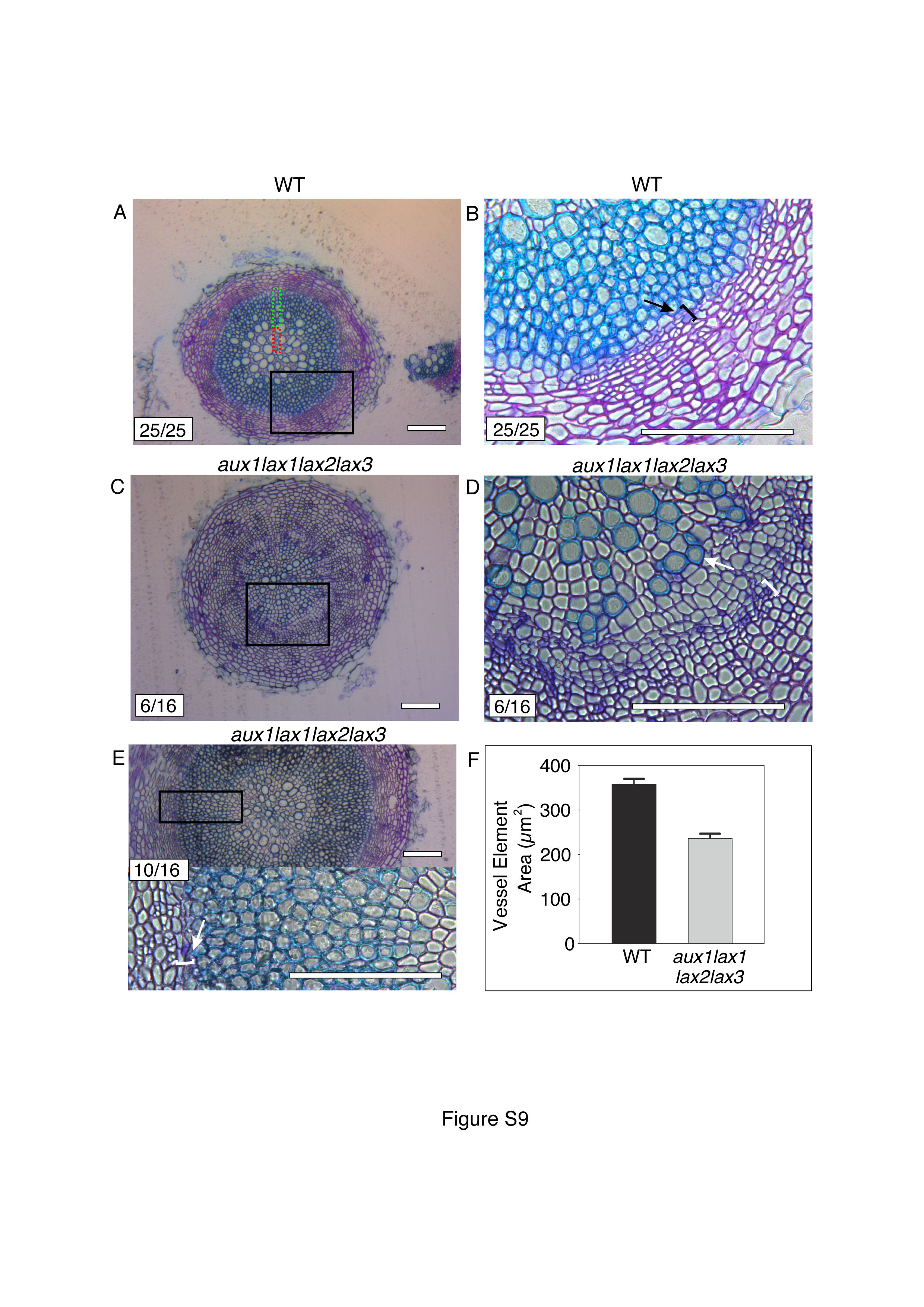

Supplement: S2 File — (ZIP) [file pgen.1005296.s002.zip › Figure S9_new.tiff]

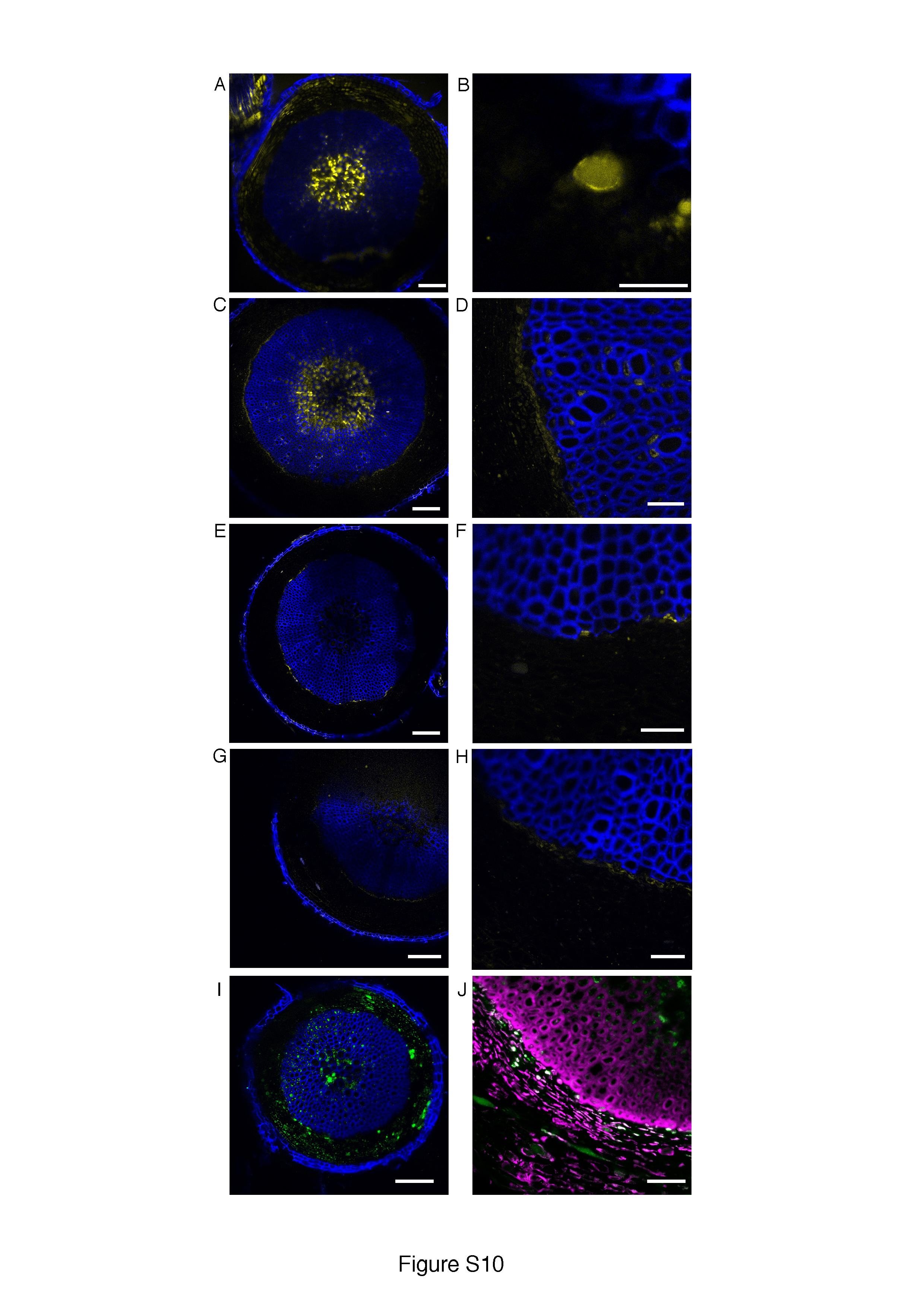

Supplement: S2 File — (ZIP) [file pgen.1005296.s002.zip › Figure S10_NEW!_V2.tiff]

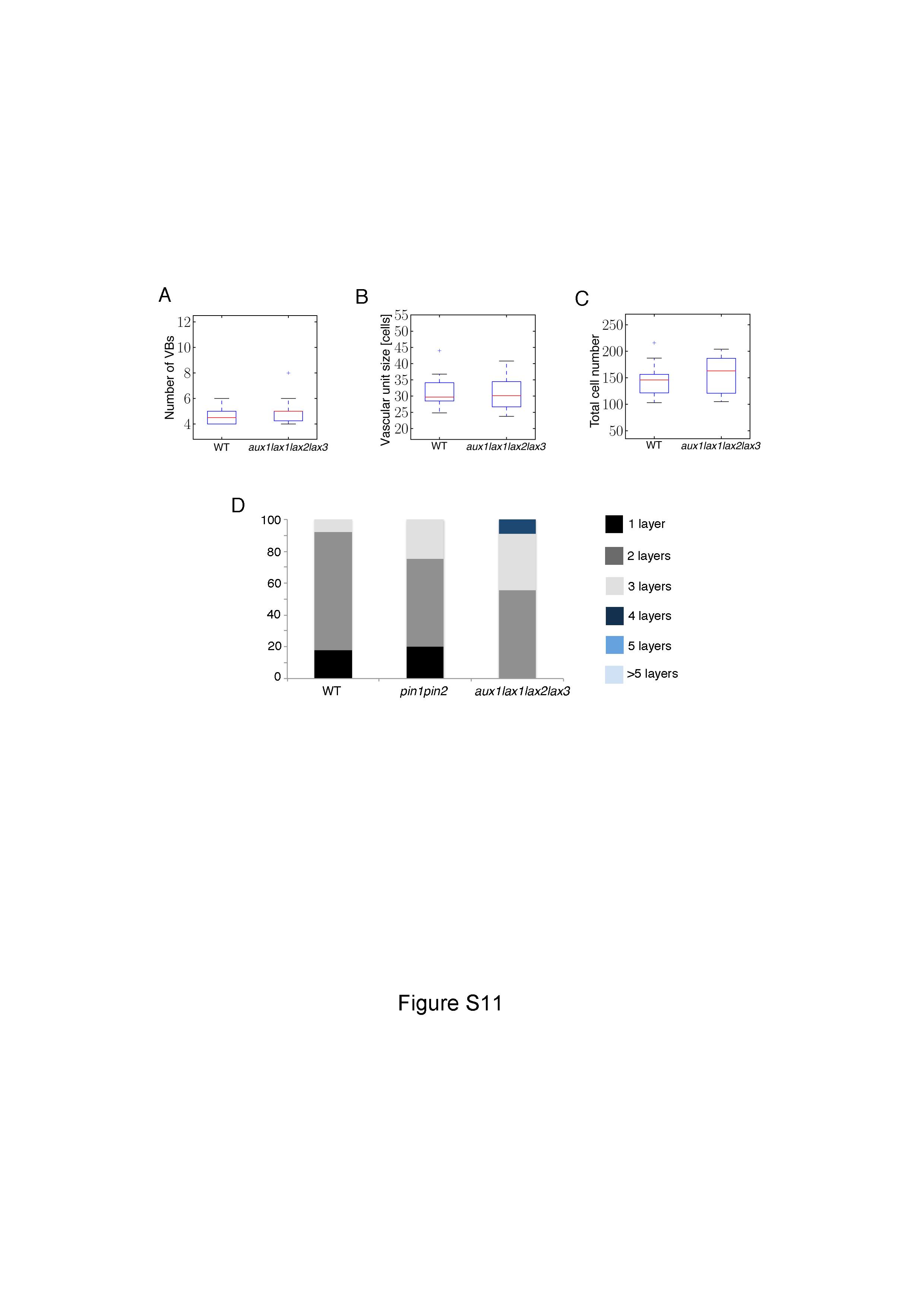

Supplement: S2 File — (ZIP) [file pgen.1005296.s002.zip › Figure S11.tiff]

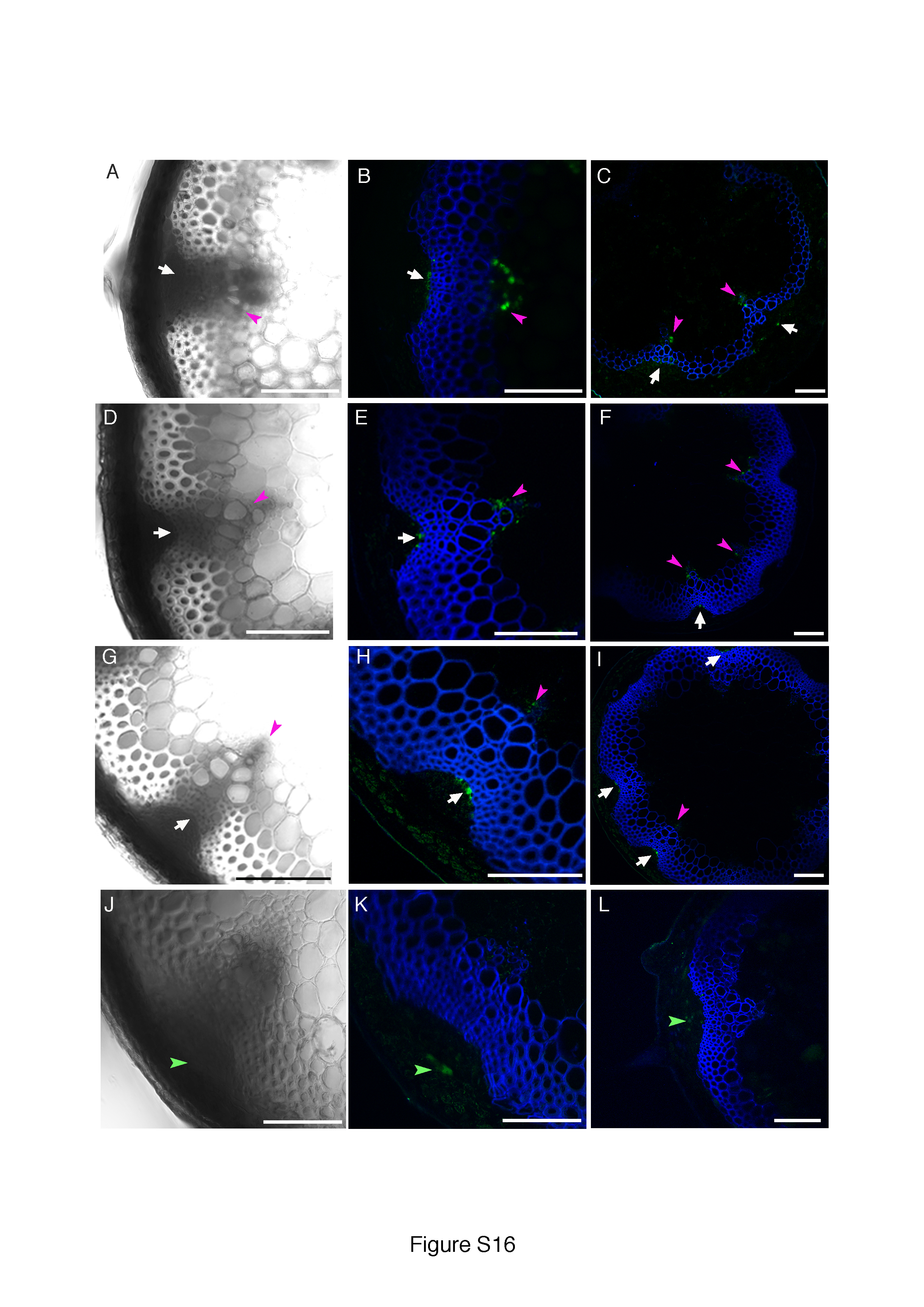

Supplement: S2 File — (ZIP) [file pgen.1005296.s002.zip › Figure S16.tiff]
